# Metabolic-pathway-based classification of tendinopathy reveals a precise therapeutic strategy targeting chondro-modulatory tenocytes

**DOI:** 10.1038/s41413-026-00574-w

**Published:** 2026-08-27

**Authors:** Junchao Luo, Zetao Wang, Ruifu Lin, Jiayun Huang, Chenqi Tang, Honglu Cai, Yang Fei, Dengfeng Ruan, Xinji Wang, Peiru Li, Menyun Liu, Ruojin Yan, Chunmei Fan, Canlong Wang, Cunqi Ye, Hongwei Ouyang, Zi Yin, Xiao Chen, Weiliang Shen

**Affiliations:** 1 Department of Sports Medicine & Orthopedic Surgery, the Second Affiliated Hospital, Zhejiang University School of Medicine, Hangzhou, China; 2 Institute of Sports Medicine, Zhejiang University, Hangzhou, China; 3 Orthopedics Research Institute, Zhejiang University, Hangzhou, China; 4 Key Laboratory of Motor System Disease Research and Precision Therapy of Zhejiang Province, Hangzhou, China; 5 Clinical Research Center of Motor System Disease of Zhejiang Province, Hangzhou, China; 6 Dr. Li Dak Sum-Yip Yio Chin Center for Stem Cells and Regenerative Medicine and Department of Sports Medicine & Orthopedic Surgery of The Second Affiliated Hospital, Zhejiang University School of Medicine, Hangzhou, China; 7 Binjiang Institute, Zhejiang University, Hangzhou, China; 8 Liangzhu Laboratory, Zhejiang University, Hangzhou, China; 9 Zhejiang Provincial Key Laboratory for Cancer transcriptional Cell Biology, Life Sciences Institute, Zhejiang University, Hangzhou, China; 10 Zhejiang University-University of Edinburgh Institute, Zhejiang University School of Medicine, Haining, China

**Keywords:** Metabolomics, Bone

## Abstract

Tendinopathy is a prevalent degenerative condition driven by complex metabolic dysregulation, yet its inherent metabolic heterogeneity remains poorly characterized, hindering targeted therapeutic development. To address this, we performed systematic multi-omics profiling of human rotator cuff tendinopathy (*n* = 192). Unsupervised clustering based on the activity of 149 metabolic pathways, validated by pseudotargeted metabolomics, revealed three distinct metabolic-pathway-based classifications (MPCs): MPC-G (glycolytic), with upregulated carbohydrate and glycan metabolism; MPC-F (fatty acid oxidative), exhibiting elevated fatty acid oxidation and amino acid metabolism; and MPC-I (imbalanced), showing dysregulated metabolite homeostasis alongside moderate glycolysis increase and fatty acid oxidation decrease. Single-cell RNA sequencing identified classification-specific pathogenic cell populations. Mechanistically, hypoxia-induced glycolytic reprogramming via HIF-1α led to lactate accumulation, which in turn stabilized HIF-1α, forming a feed-forward loop that promoted the formation of chondro-modulatory tenocytes and drove MPC-G pathogenesis. Genetic inhibition of HIF-1α and direct targeting of lactate both attenuated disease progression, validating the critical role of this axis. Notably, we identified and validated the clinical drug temsirolimus as an effective agent specifically against MPC-G in experimental models. Collectively, our study delineates the metabolic heterogeneity of tendinopathy and establishes a classification framework that enables precision medicine strategies tailored to distinct metabolic profiles.

**Figure:**
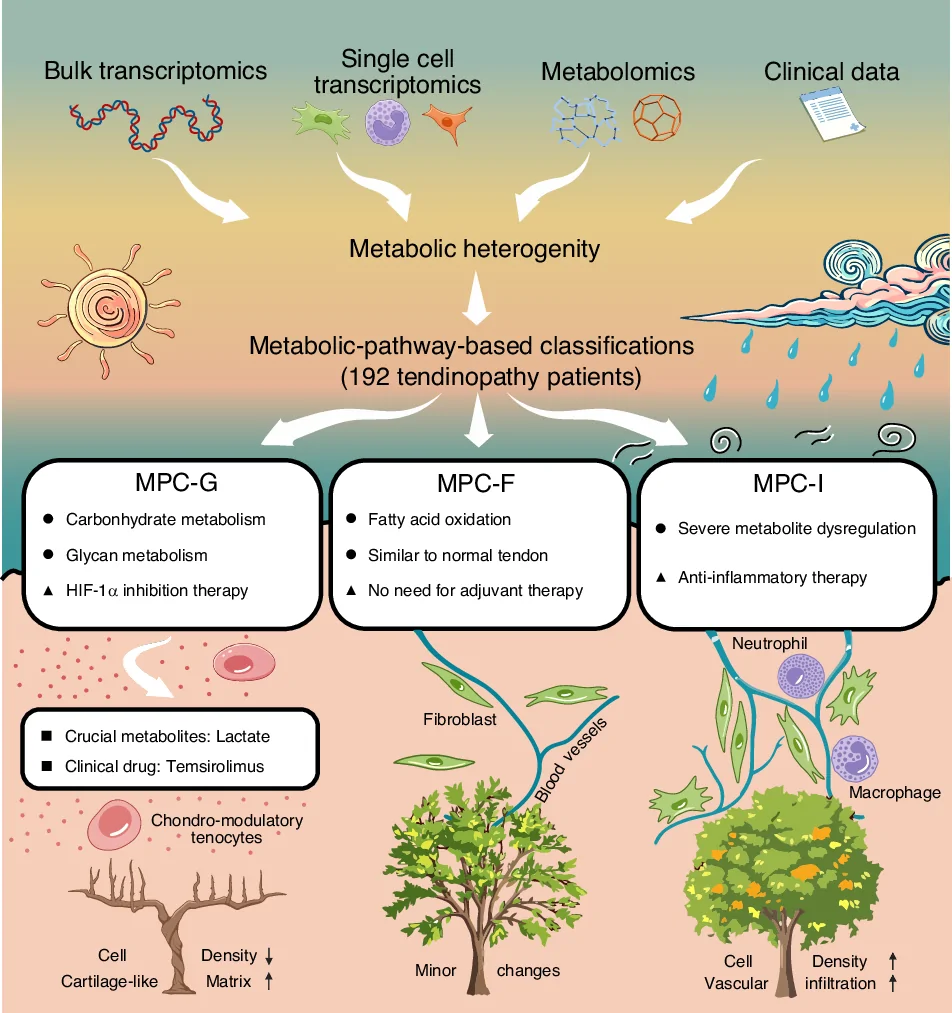

## Introduction

Metabolic disorders play an important role in the development of tendinopathy. Diabetes, hypercholesterolemia, obesity, and other metabolic diseases have been reported to significantly increase the risk of tendinopathy.^1–4^ Common pathogenic factors such as aging, overloading, and inflammation can also lead to changes in metabolic status.^5,6^ For example, age-related mitochondrial dysfunction can lead to insufficient cellular energy supply, while compressive loads can cause a significant enhancement of glycolysis.^7,8^ These metabolic alterations affect the functional status and fate of tendon cells as well as other types of cells in the tendon microenvironment, ultimately driving or delaying the course of tendinopathy. Revealing the metabolic landscape of tendinopathy is of great significance for explaining the occurrence and development of tendinopathy and exploring therapeutic targets.

Rotator cuff tendinopathy is the most common tendinopathy and the leading cause of shoulder pain.^9^ As the disease progresses, it eventually develops into a rotator cuff tear. Surgical repair is the most used treatment strategy. However, the outcome of surgery is highly heterogeneous, with retear failure rates ranging from 11% to 94%.^10^ The effectiveness of adjunctive therapies, such as glucocorticoid steroids and platelet-rich plasma local injections, are also highly heterogeneous across studies.^11–13^ In previous studies, we classified rotator cuff tendinopathy into two transcriptional subtypes with distinct clinical and genomic characteristics: inflammatory proliferative (I) and hypoxic atrophic (H), and developed clinically effective treatment strategies for subtype I.^14^ However, our understanding of the metabolic heterogeneity of tendinopathy is still lacking, which may limit the further development of precision diagnosis and treatment. In this study, we aim to comprehensively assess which metabolic pathways have altered the overall metabolic transcriptional profiles in tendinopathy. We identified three distinct metabolic classifications of tendinopathy based on 148 metabolic pathways: MPC-G, MPC-F and MPC-I. These three metabolic-pathway-based classifications (MPCs) have distinct metabolic gene expression, metabolite abundance, clinical features, histological features, pathogenesis and drug sensitivity.

## Results

### Multiple omics reveal metabolic dysregulation in rotator cuff tendinopathy

**Fig. 1:**
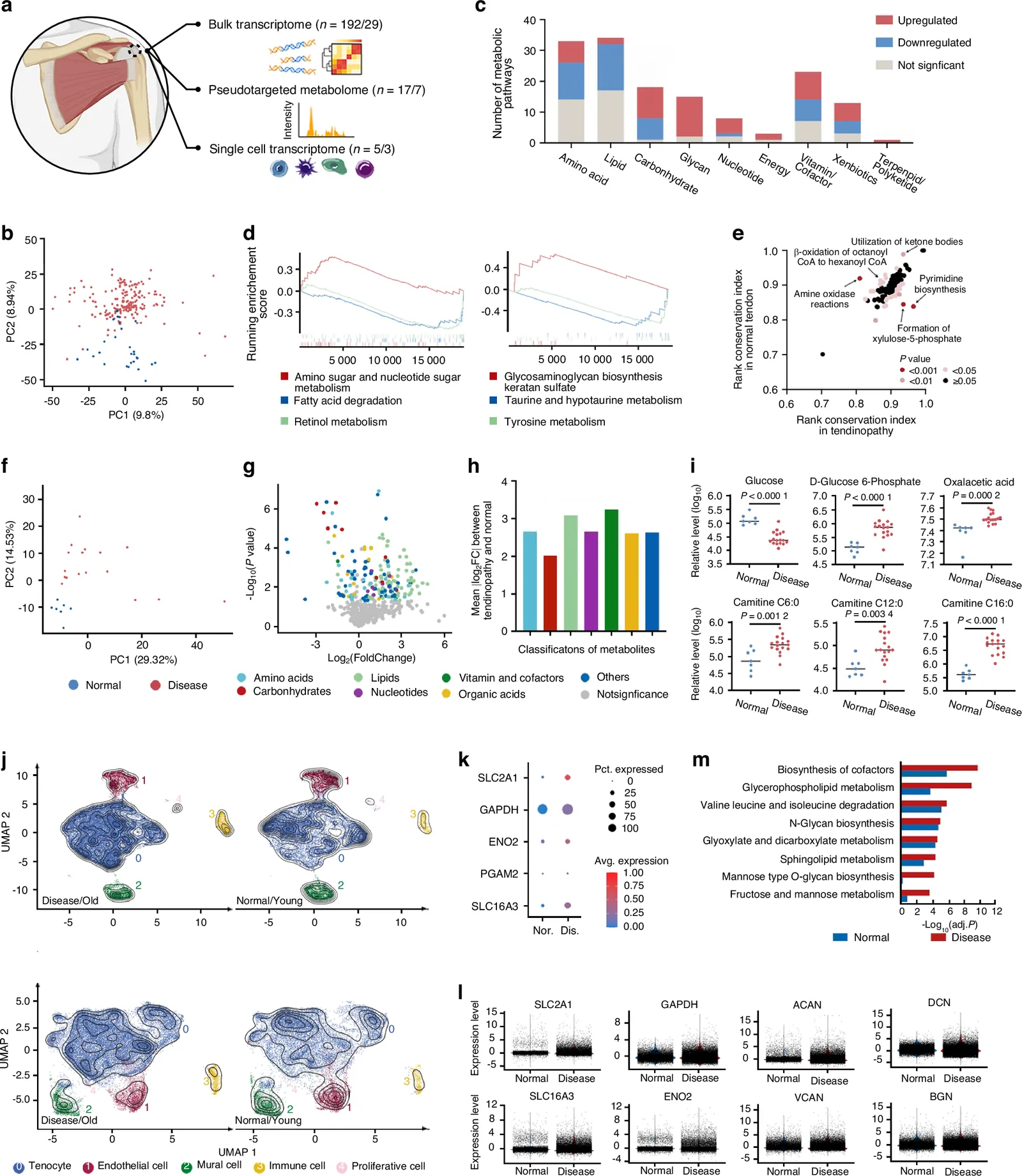
Metabolic dysregulation of rotator cuff tendinopathy. **a** Study design overview. Bulk RNA-seq (*n* = 192 *diseased*, *n* = 29 *normal*), LC-MS/MS (*n* = 17 *diseased*, *n* = 7 *normal*) and single-cell RNA-seq (*n* = 5 *diseased*, *n* = 3 *normal*) were performed. **b** PCA of metabolic gene expression in diseased and normal samples. **c** Number of metabolic pathways with significantly altered activity in diseased versus normal samples across nine categories. **d** Representative GSEA plots show significantly altered pathways. **e** Comparison of the RCI of metabolic pathways between tendinopathy and normal samples. **f** PCA of metabolite abundance in diseased and normal samples. **g** Volcano plot of 735 annotated metabolites. **h** Degree of overall metabolomic dysregulation in tendinopathy. **i** Relative intensity levels of differentially abundant metabolites in glycolysis (Up) and fatty acid oxidation (Down). **j** UMAP plot of five tendon cell types from five diseased and three normal tendons based on all gene (Up) or metabolic gene (Down) expression. **k**, **l** Average expression levels and violin plots of representative glycolysis and glycan genes in diseased versus normal tenocytes. **m** KEGG enrichment analysis of representative gene markers for diseased tissue-derived and normal tissue derived fibroblasts. The data were presented as the mean ± SD. Statistically significant differences are indicated by *P* < 0.05 between the indicated groups

To investigate metabolic dysregulation in rotator cuff tendinopathy, we performed bulk RNA-seq on diseased tendon samples from 192 patients undergoing rotator cuff repair and on normal hamstring tendons from 29 patients undergoing anterior cruciate ligament reconstruction (Table S1, Fig.S1). A subset of these (17 diseased, 7 normal) underwent LC-MS/MS analysis, and an additional independent set (5 diseased, 3 normal) was used for single-cell RNA-seq(Fig.1a). We extracted 80 metabolic pathways involving 1 707 human genes from the Kyoto Encyclopedia of Genes and Genomes (KEGG) database and supplemented 68 metabolic pathways involving 1 185 human genes from the Reactome database. 2 732 metabolic genes were obtained after eliminating duplicates (Table S2-3). Principal component analysis (PCA) revealed significant differences in metabolic gene expression between tendinopathy tendons and normal tendons (Fig. 1b). We used Gene Set Variation Analysis (GSVA) to calculate the enrichment scores of 148 metabolic pathways in each specimen and used limma package for difference analysis. The results showed that 10/17 carbohydrate metabolic pathways, 13/14 glycan metabolic pathways, and 9/20 vitamin/cofactor metabolic pathways exhibited relatively higher activity in diseased tendons, whereas 12/33 amino acid metabolic pathways and 15/35 lipid metabolic pathways were more active in normal tendons (Fig. 1c). Gene set enrichment analysis (GSEA) further revealed that diseased tendons exhibited higher activity in the amino sugar and nucleotide sugar metabolism pathway, as well as glycosaminoglycan biosynthesis keratan sulfate pathway, but lower activity in the fatty acid degradation pathway, tyrosine pathway, and taurine and hypotaurine pathway (Fig. 1d). Furthermore, we used differential rank conservation analysis to quantify conservation differences of metabolic pathways for diseased and normal specimens.^15^ Lipid utilization related pathways showed small rank conservation indexes, which means higher variability, in tendinopathy (*P* < 0.05) (Fig. 1e).

Mass spectrometry detection annotated 735 metabolites in these samples. We found a significant change of metabolite abundance in diseased tissues compared to normal tissues (Fig. 1f), especially vitamin/cofactor and lipids (Fig. 1g, h). The abundance of glucose in the diseased tissue was significantly reduced, while the downstream product was increased, suggesting an enhancement of glucose utilization (Fig. 1i). In addition, the accumulation of various fatty acylcarnitines appeared in the diseased tissues, suggesting that downstream fatty acid oxidation was blocked (Fig. 1i). These results were consistent with transcriptome findings that the pattern of energy metabolism in the disease tendon had shifted from predominantly utilizing fatty acids to predominantly utilizing glucose.

In single-cell transcriptome analysis, a total of 59 541 viable, single cells from both diseased and normal tendon samples were partitioned into subsets (Fig. S1A-B), including 21 734 cells (36.5%) from normal tendons and 37 807 cells (63.5%) from diseased tendons. The proportion of endothelial cells and mural cells was higher in diseased tendons (Fig. S1C). There was significant metabolic heterogeneity among different cell types or cells of the same type but derived from diseased and normal tissues, respectively (Fig. 1j; Fig. S1D). Glucose transporter, such as solute carrier family 2 member 1 (SLC2A1), lactate transporter, such as solute carrier family 16 member 3 (SLC16A3), as well as glycolytic-related enzymes, such as glyceraldehyde 3-phosphate dehydrogenase (GAPDH) and enolase 2 (ENO2) had a relatively high mRNA expression level and cell expressed proportion in diseased tenocytes. Besides, diseased tenocytes also highly expressed a variety of glycan genes(Fig. 1k, l) Enrichment analysis based on upregulated differential genes suggested that compared with normal tenocytes, upregulated differential genes of diseased tenocytes were more enriched in biosynthesis of cofactors, purine metabolism, amino sugar and nucleotide sugar metabolism, as well as fructose and mannose metabolism (Fig. 1m). Overall, the diseased tissue-derived tenocytes did show enhanced carbohydrate metabolism.

### Metabolic-pathway-based classification of rotator cuff tendinopathy

**Fig. 2:**
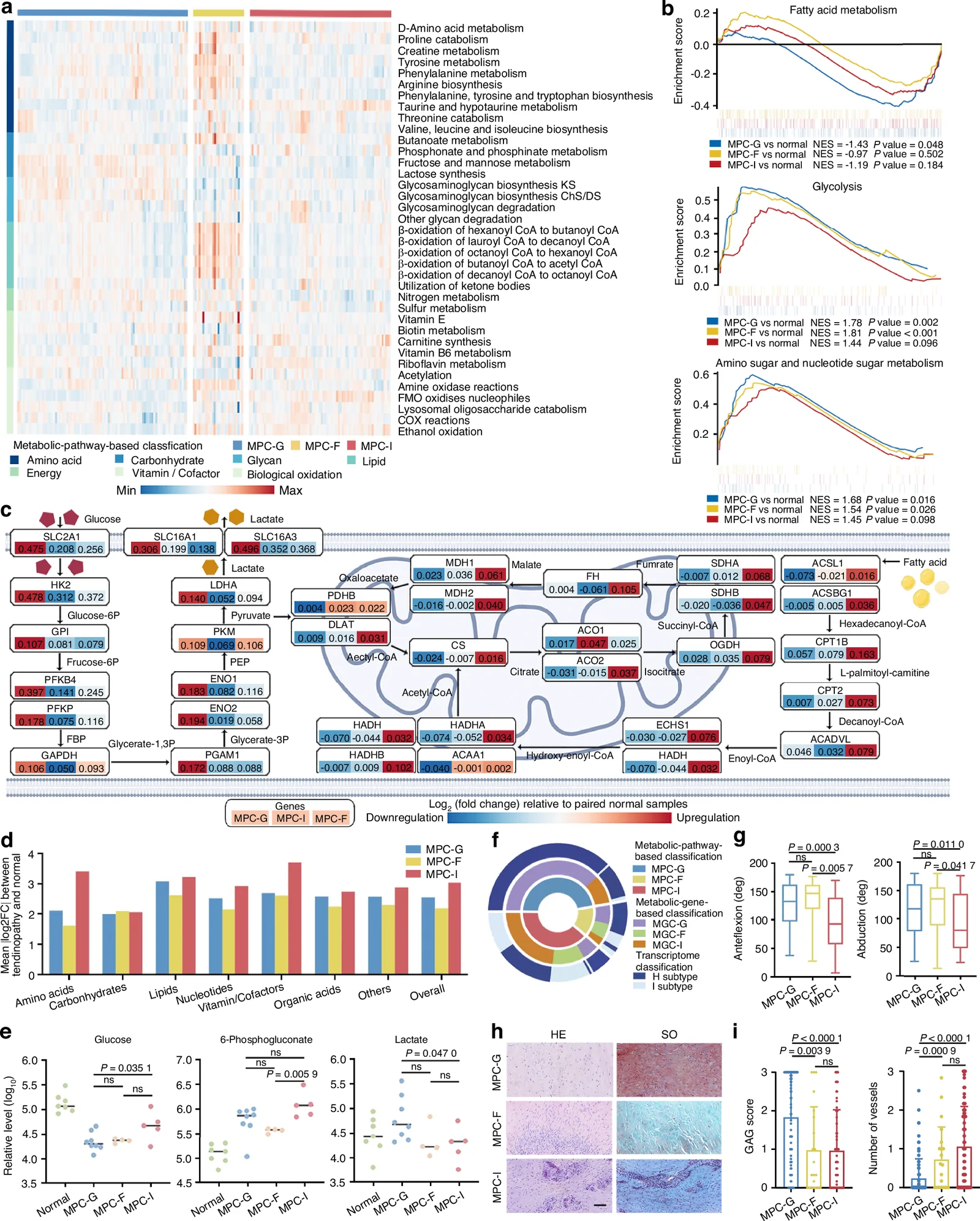
Metabolic-pathway-based classification of human tendinopathy. **a** Metabolic-pathway-based subseting results of tendinopathy samples (*n* = 192). Heatmap shows normalized enrichment scores of the three MPCs (top 25% pathways by standard error). **b** Representative GSEA plots of differentially enriched pathways among three MPCs and normal tissues. **c** Diagram of metabolic genes in glycolysis, TCA cycle, and taurine metabolism. Alteration scores of each gene are depicted as log ratios (fold-change, expressed as log_2_[ratio of average mRNA expression in each MPCs versus normal group]). **d** Degree of overall metabolomic dysregulation among three MPCs. **e** Relative intensity levels of metabolites in glucose utilization across three MPCs. **f** Overlap of MPCs with metabolic-gene-based classifications and published transcriptional subtypes. **g** Preoperative range of motion of shoulder joint. **h** Representative histological images of MPC-G, MPC-F and MPC-I. Scale bar, 100 μm. **i** GAG score and vascular density among three MPCs. The data were presented as the mean ± SD. Statistically significant differences are indicated by *P* < 0.05 between the indicated groups

To reveal the metabolic heterogeneity of rotator cuff tendinopathy, we first attempted to perform non-negative matrix factorization (NMF) consensus subset analysis on 192 tendinopathy samples based on metabolic genes. The cophenetic coefficient suggested that the subseting was the most stable when the samples were classified into 3 classifications (Fig. S3A-D). However, the consensus matrix indicated that there was still high variation within the subset (Fig. S3B). The enrichment score of pathways could reflect the activity of specific pathway.^16^ We found that subset analysis based on the enrichment scores of 148 metabolic pathways could stably classify the samples into 3 heterogeneous metabolic-pathway-based classifications (MPCs) (Fig. S3E-F; Fig. S4A; Fig. S6A-E). MPC-G (46.8% of all tendinopathy samples), designated the glycolytic classification, was characterized by the relative upregulation of carbohydrate and glycan metabolisms, including glycolysis, amino sugar, and nucleotide sugar metabolism, as well as glycosaminoglycan biosynthesis (Fig. 2a; Fig. S4B). This pattern was consistent with metabolic characteristics in hypoxic environment. MPC-F (14% of all tendinopathy samples), designated the fatty acid oxidation classification, was characterized by the high levels of fatty acid oxidation and amino acid metabolism, including proline, tyrosine, phenylalanine and arginine metabolism (Fig. 2a; Fig. S4B). MPC-I (39% of all tendinopathy samples), designated the imbalance classification, had levels of glycolysis and fatty acid oxidation between the other two classifications. This classification was characterized by the relative upregulation of taurine-related metabolism, arachidonic acid metabolism, folate biosynthesis, and riboflavin metabolism (Fig. 2a; Fig. S4B). Among them, MPC-F and normal tendons enjoyed similar metabolic patterns, including relative high levels of fatty acid oxidation and amino acid metabolism (Fig. 2b; Fig. S4C). Interestingly, MPC-F also showed metabolic features of hypoxic environments, such as increased glycolysis (Fig. 2b; Fig. S4C).

Next, we determined whether expression of metabolic enzymes and corresponding metabolite abundance are linked to the enrichment scores of metabolic pathways in different classifications (Fig. 2c). Tendinopathy within the MPC-G were enriched for expression of genes involved in glycolysis. These included Hexokinase 2 (HK), glucose-6-phosphate isomerase (GPI), phosphofructokinase (PFKP), Phosphoglycerate mutase (PGAM), enolase-1 (ENO1) and lactate dehydrogenase A (LDHA). We also noted that mRNA expression of the glucose transporter, SLC2A1 and the lactate transporter, solute carrier family 16 member 3 (SLC16A3), were upregulated in MPC-G. Immunohistochemistry confirmed that there were significant differences in the protein expression of SLC2A1, HK2 and LDHA among the three classifications (Fig. S7A-B). In contrast, the expression of metabolic enzymes in fatty acid oxidation and tricarboxylic acid cycle had higher levels in MPC-F. For example, MPC-F demonstrated higher expression of acyl-CoA synthetase bubblegum (ACSBG), acyl-CoA dehydrogenase very long chain (ACADVL), enoyl-CoA hydratase (ECHS), hydroxyacyl-CoA dehydrogenase (HADH) and other fatty acid-oxidization-related enzymes. The expression of carnitine palmitoyltransferase 1 (CPT1) and carnitine palmitoyltransferase 2 (CPT2) was also high. In tricarboxylic acid cycle, the expression of aconitase 2 (ACO2), oxoglutarate dehydrogenase (OGDH), succinate dehydrogenase (SDH), malate dehydrogenase (MDH) and citrate synthase (CS) increased. Pseudotargeted metabolomics validated the transcriptome-based classification, revealing significant differences in metabolite abundance among the three MPCs (Fig. S5A-C). MPC-F showed relatively mild metabolite dysregulation, while MPC-I showed relatively severe metabolite dysregulation, especially in the accumulation of amino acids and vitamins (Fig. 2d). In agreement with increased glycolytic gene expression, MPC-G demonstrated with lower levels of glucose, the substrate for glycolysis. This classification was also distinguished by the accumulation of intermediates in glycolysis, including glucose 6-phosphate and lactate (Fig. 2e). Furthermore, we observed the accumulation of various acyl carnitines in MPC-G, which is consistent with transcriptional downregulation of fatty acid oxidation pathways and may reflect impaired fatty acid oxidation (Fig. S5D). MPC-I had a significant accumulation of various vitamins, such as thiamine and riboflavin (Fig. S5E). Notably, MPC-I also showed the accumulation of various amino acids, which may be associated with higher levels of collagen turnover (Fig. S5F). Therefore, we demonstrated that metabolic enzyme expressions and metabolite abundances agreed well to differentiate the three MPCs.

In previous studies, we identified transcriptional classifications of rotator cuff tendinopathy based on gene expression.^14^ We determined the HI transcriptional subtypes for each sample and investigated their overlap with the MPCs (Fig. 2f). The metabolomic MPC-G almost overlapped with the transcriptional H subtype; MPC-I had the highest proportion of samples classified as transcriptional I subtype; and about two-thirds of the samples in MPC-F were classified as transcriptional H subtype.

Furthermore, the three MPCs demonstrated distinct clinical characteristics. The body mass index (BMI) showed significant differences among the three classifications in the general clinical characteristics, with the highest proportion of patients having a normal BMI observed in the MPC-F classification (Table S4). Tendons of MPC-G and MPC-F were predominantly white, while nearly half of MPC-I tendons appeared red. Histologically, MPC-G exhibited lower cell and blood vessel density with cartilage-like matrix accumulation, whereas MPC-I showed higher cell and blood vessel density. MPC-F displayed the mildest histological lesions (Fig. 2h, i; Fig. S5I). The red discolouration in MPC-I likely reflects increased vascularity and inflammatory infiltration, consistent with its higher vascular density. Conversely, the whitish appearance in MPC-G and MPC-F may be associated with reduced cellularity, matrix remodeling, and hypoxia-related degeneration. Similarly, the joint synovium was more frequently red in MPC-I and least often in MPC-F (Fig. S5G). Compared with other classifications, MPC-I had significantly lower range of activity in anteflexion, abduction and external rotation (Fig. 2g; Fig. S5H). In hematological parameters, MPC-F showed the highest albumin/globulin ratio and MPC-I the lowest (Fig. S8A). Given that liver function indicators, such as alanine aminotransferase, aspartate aminotransferase and bilirubin, did not differ significantly among the three MPCs, the reduced A/G ratio in MPC-I may reflect systemic metabolic alterations or nutritional status associated with this classification, though further studies are warranted. Additionally, MPC-I showed higher proportion of glycated albumin, while MPC-G showed longer thrombin time (Fig. S8B-C). Collectively, our results demonstrated that the metabolic heterogeneity of rotator cuff tendinopathy fell into three metabolic phenotypes. The transcriptional H subtype lacking effective treatment strategies almost overlapped with the metabolomic MPC-G. MPC-F exhibited relatively mild metabolic dysregulation and histological changes, suggesting a phenotype with the least severe pathological alterations.

### Hypoxia and inflammation control the metabolic fate of tendinopathy

**Fig. 3:**
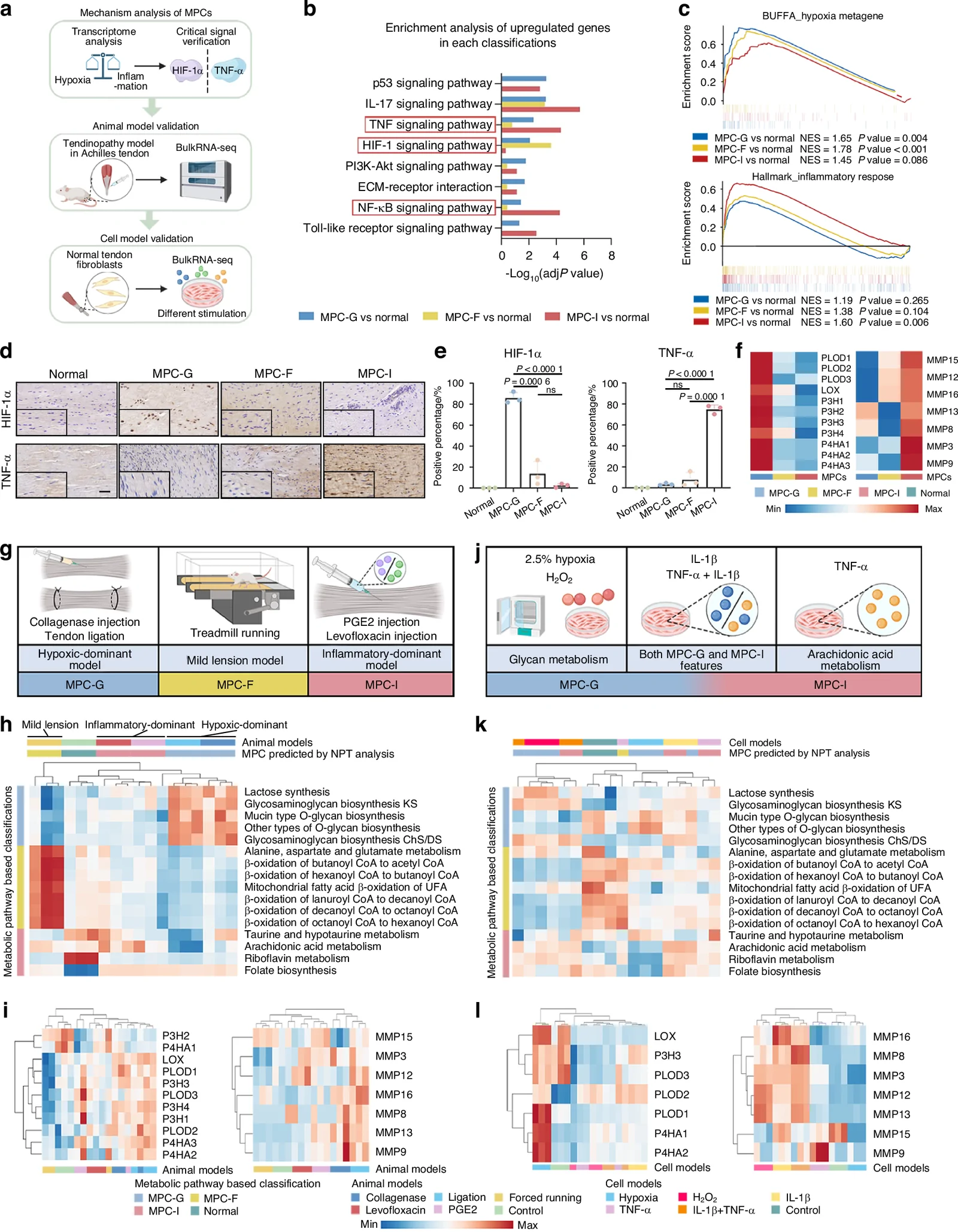
Hypoxia and inflammation control the metabolic fate of tendinopathy. **a** Schematic diagram of the workflow to resolve MPCs. **b** KEGG enrichment analysis for differentially expressed metabolic genes between MPCs and normal samples in human. **c** Representative GSEA plots of inflammation and hypoxia pathways among three MPCs and normal samples in human. **d-e** Immunohistochemistry of *HIF-1α* and *TNF-α* and semi-quantitative analysis in human MPCs. Scale bar, 50 μm (*n* = 3) **f** Expression changes of collagen-modifying and matrix-degrading enzymes among three MPCs. Alteration scores are log_2_(fold-change vs normal). **g** Illustration of inflammatory-dominant, hypoxic-dominant, and mild lesion animal models. **h** NTP analysis predicting the correspondence between five animal models and MPCs (*n* = 3) **i** Expression of collagen-modifying enzymes and matrix metal-degrading enzymes in different animal models. **j** Illustration of the construction of cell models induced by 2.5% hypoxia, H_2_O_2_, IL-1β, TNF-α and IL-1β + TNF-α. **k** NTP analysis predicting the correspondence between five cell models and MPCs (*n* = 3) **l** Expression of collagen-modifying enzymes and matrix metal-degrading enzymes in different cell models. The data were presented as the mean ± SD. Statistically significant differences are indicated by *P* < 0.05 between the indicated groups

We further investigated the characteristic signaling pathway of each MPC to clarify their mechanisms (Fig. 3a). We extracted the differentially expressed genes that are increased in each MPC compared to normal tendons. As shown in Fig. 3b, the genes with increased expression in MPC-I and MPC-G, especially MPC-I, were significantly enriched in inflammation-related pathways, such as the tumor necrosis factor (TNF) signaling pathway and the NF-kappaB (NF-kB) signaling pathway, while the genes in MPC-G and MPC-F were significantly enriched in the HIF-1α signaling pathway. GSEA confirmed the association of MPC-G/F with hypoxia and MPC-I with inflammation (Fig. 3c). Immunohistochemistry validated higher protein levels of HIF-1α in MPC-G and TNF-α in MPC-I (Fig. 3d, e). Correspondingly, mRNA expression of hypoxia-driven collagen-modifying enzymes was elevated in MPC-G, while inflammation-driven matrix-degrading enzymes were elevated in MPC-I (Fig. 3f). Previous studies have reported that increased expression of collagen-modifying enzymes, such as prolyl 4-hydroxylase subunit alpha1 (P4HA1) and procollagen-lysine,2-oxoglutarate 5-dioxygenase 2 (PLOD2), inhibited the degradation of cartilage-like matrix.^17^ Given that MPC-F exhibited the mildest metabolic dysregulation among the three subtypes, we focused our mechanistic hypothesis on the other two classifications: we propose that MPC-I tendinopathy is primarily driven by inflammation, whereas MPC-G arises from a combination of hypoxia and inflammation. To test this, we leveraged our prior classification of tendinopathy animal models^18^ to establish rat models simulating distinct etiologies: inflammatory-dominant (levofloxacin/PGE2 injection), hypoxic-dominant (collagenase injection/tendon ligation), and mild lesion (forced running) (Fig. 3g; Fig S9A). These models recapitulated expected cytokine and hypoxia pathway activation (Fig. S9B, E). Using Nearest Template Prediction (NTP) analysis based on MPC-defining pathways, we found that inflammatory models matched the MPC-I metabolic profile, hypoxic models matched MPC-G, and the mild model matched MPC-F (Fig. 3h; Fig S9F). These models also showed histological features consistent with their corresponding MPC. Inflammatory models showed increased cell and vascular density, while hypoxic models showed rounded cell morphology and enhanced cartilage-like matrix accumulation (Fig. S9C, D). In addition, mRNA expression of collagen-modifying enzymes was upregulated in hypoxic models, while mRNA expression of matrix metalloproteinases (MMPs) was upregulated in both hypoxic models and inflammatory models (Fig. 3i).

In human tendon fibroblasts, we assessed the effects of key stimuli, including hypoxia (2.5% O_2_), H_2_O_2_, interleukin (IL) -1β and TNF-α (Fig. 3j; Fig. S10A). Proliferation responses varied by stimulus (Fig. S10B-D). Pathway analysis showed IL-1β/TNF-α/IL-1β + TNF-α activated NF-kB, H_2_O_2_ activated both NF-kB and HIF-1α, and hypoxia activated only HIF-1α (Fig. S10E-G). GSVA analysis further detailed these effects: hypoxia significantly activated angiogenesis pathways, while IL-1β, H_2_O_2_, and IL-1β + TNF-α potently activated chondrogenic and osteogenic differentiation pathways (Fig. S11D-F). Despite its minimal effect on overall chondrogenic pathway activation in the GSVA, hypoxia specifically upregulated SRY-box transcription factor 9 (SOX9), a transcription factor associated with chondrogenesis whose expression is elevated in pathological conditions (Fig. S11A). Additionally, hypoxia and H_2_O_2_ both increased expression of collagen-modifying enzymes (Fig. S11A-C). NTP prediction classified H_2_O_2_ and hypoxia-treated cells as MPC-G, TNF-α-treated as MPC-I, and IL-1β/ IL-1β + TNF-α treatments as mixed MPC-G/I (Fig. 3k). All treatments downregulated fatty acid oxidation (Fig. 3k; Fig S10H). Glycolysis was significantly activated by IL-1β and hypoxia (Fig. S10I). The representative metabolic pathways of MPC-I, including taurine and hypotaurine metabolism, arachidonic acid metabolism, folate biosynthesis, and riboflavin metabolism, were upregulated by oxidative stress and inflammatory stimulation, but downregulated by hypoxia (Fig. 3k). Some representative pathways of MPC-G, such as glycosaminoglycan synthesis, were upregulated by IL-1β, H_2_O_2_, and IL-1β + TNF-α, while the other representative pathways of MPC-G, such as polysaccharide synthesis were upregulated by hypoxia (Fig. 3k). These results supported our hypothesis that MPC-I arised primarily from inflammation, whereas MPC-G resulted from a combination of inflammation and hypoxia. Collectively, our study revealed how hypoxia and inflammation determined the metabolic fate and histological phenotype of tendinopathy. TNF-α and IL-1β may be the key signals inducing MPC-I tendinopathy, while hypoxia and IL-1β may be the key signals inducing MPC-G tendinopathy. Both hypoxia and IL-1β significantly induced glycolytic reprogramming of fibroblasts. IL-1β stimulation and H_2_O_2_-mediated oxidative stress activated pathways associated with chondrogenic differentiation. However, these stimuli also induced the expression of multiple extracellular matrix-degrading enzymes. This may be the reason why MPC-I did not develop cartilage-like matrix accumulation. Hypoxia enhanced the ability of cartilage-like matrix to resist degradation by inducing the expression of collagen-modifying enzymes.

### Hypoxia drives chondro-modulatory tenocytes formation to mediate MPC-G

**Fig. 4:**
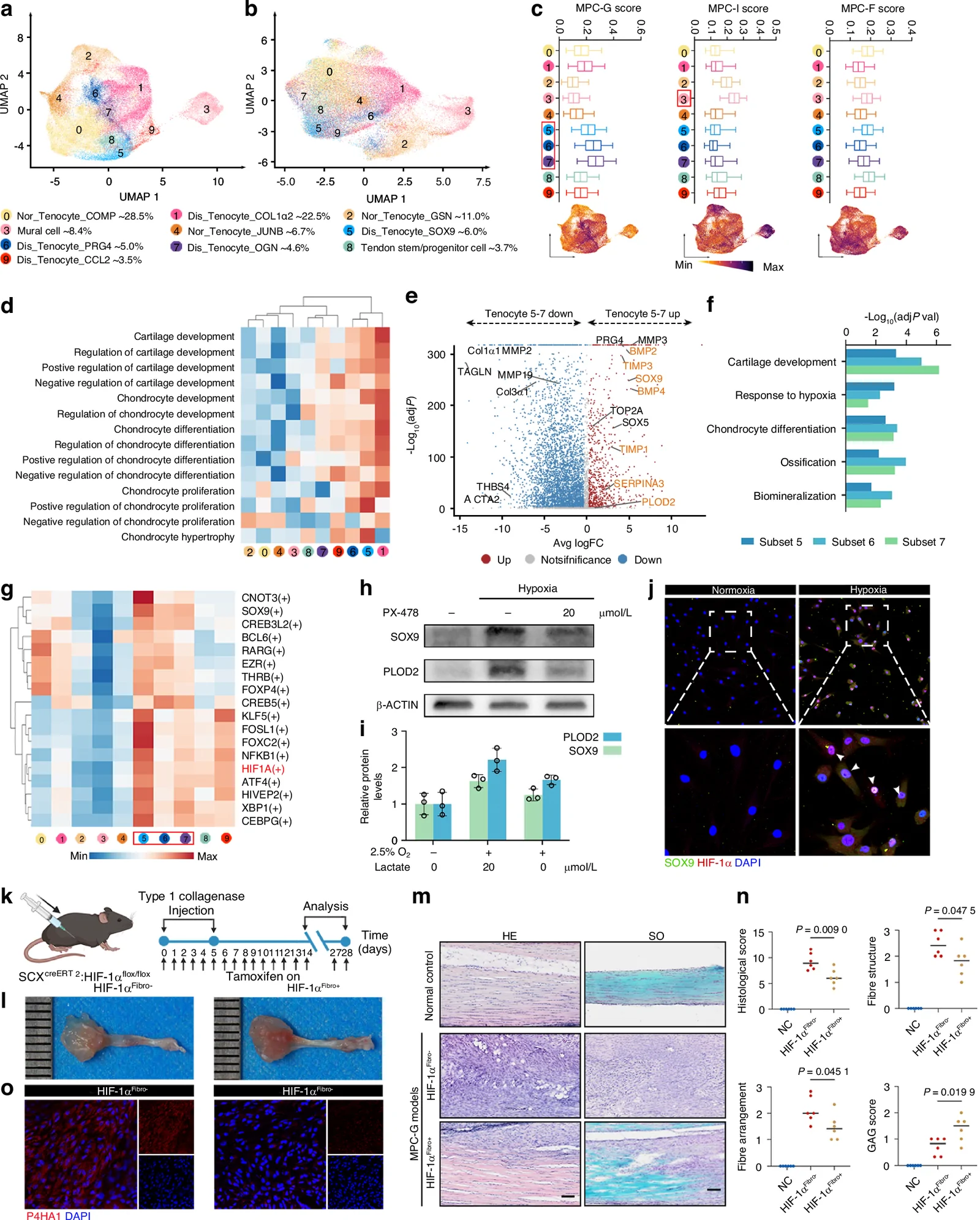
HIF-1α drives chondro-modulatory tenocyte formation in MPC-G tendinopathy. **a**, **b** UMAP plots of 8 stromal cell subsets based on all genes (Right) or metabolic genes (Left). **c** MPC-G, MPC-F, and MPC-I scores per subset using the Ucell function (top 25% differential metabolic genes). **d** GSVA of chondrification-related GO pathways in chondro-modulatory tenocytes (subsets 5, 6, 7) **e** Volcano plot displaying differentially expressed genes between chondro-modulatory tenocytes and other subsets. **f** KEGG enrichment analysis revealed enrichment of marker genes from chondro-modulatory tenocytes in chondrogenesis, osteogenesis, and hypoxia-related pathways. (*P* < 0.05, logFC > 0.25) **g** SCENIC analysis identifying *HIF-1α* and *SOX9* as key regulators. **h–l** Western blot and semi-quantitative analysis of *SOX9* and *PLOD2* under normoxia, hypoxia, or hypoxia with PX-478. **j** Immunofluorescence of *HIF-1α* and *SOX9* co-localization under hypoxia. Scale bar, 50 μm. **k** Schematic of *HIF-1α* knockdown experiment. **l** Representative images of Achilles tendon in MPC-G model from HIF-1α^fibro+^ and HIF-1α^fibro-^ mice. **m** Representative H&E and S.O. staining in MPC-G model from HIF-1α^fibro+^ and HIF-1α^fibro-^ mice. Scale bar, 100 μm. **n** Immunofluorescence showing the effect of *HIF-1α* knockout on the *P4HA1* expressionin. Scale bar, 50 μm. **o** Histological total score, fiber structure, fiber arrangement, and GAG score. The data were presented as the mean ± SD. Statistically significant differences are indicated by *P* < 0.05 between the indicated groups

To identify pathogenic cell populations, we analyzed stromal cells (tenocytes and mural cells) from single-cell data, resolving 10 distinct subsets (Fig. 4a; Fig S12A-D). Several subsets were enriched in diseased tendons, including those expressing COL1α2 (subset 1), SOX9 (subset 5), PRG4 (subset 6), OGN (subset 7), and CCL2 (subset 9) (Fig. S12D). Significant metabolic heterogeneity existed among these fibroblast subsets (Fig. S12C). By scoring cells based on MPC-specific metabolic gene signatures, we found subsets 5, 6, and 7 had high MPC-G scores, while subset 3 had a high MPC-I score, indicating they are likely pathogenic for their respective classifications (Fig. 4c). In contrast, MPC-F scores were similar across all subsets, implying that this classification may represents a transitional state during the shift of tendon tissue from normal to pathological conditions. Using bulk transcriptome data, we developed a random forest classifier to predict the subtypes of individual single-cell patients (Fig. S13B). The results confirmed that subsets 5, 6, and 7 were predominantly enriched in MPC-G, while subset 3 was enriched in MPC-I (Fig. S13C). Considering the high overlap of transcriptional H subtypes and metabolic MPC-G, and the lack of effective treatments, we focused on MPC-G pathogenic cells (subsets 5, 6, 7). Given the strong overlap between transcriptional H subtypes and the metabolic MPC-G signature, along with the lack of effective treatments, we focused on MPC-G pathogenic cells (subsets 5, 6, 7). As shown in Fig. S13B, cells from subsets 5, 6, and 7 derived from MPC-G patients exhibited significantly higher MPC-G scores compared to those from patients with other metabolic pathway subtypes, further supporting the pathogenic role of this cell population in MPC-G tendinopathy. These cells exhibited enhanced carbohydrate metabolism, particularly glycolysis, consistent with the MPC-G phenotype. They also showed high expression of chondrogenesis-related genes (e.g., SOX5/9, BMP2/4) and genes involved in inhibiting cartilage-like matrix degradation (e.g., TIMP1/3, PLOD2), defining them as chondro-modulatory tenocytes. (Fig. 4e). These cells showed strong enrichment for chondrogenic biological processes, and their markers were enriched in chondrogenic, osteogenic, and hypoxia pathways (Fig. 4d, f; Fig. S13D). Immunofluorescence confirmed that tenocytes in MPC-G samples expressed elevated levels of genes associated with cartilage-like matrix synthesis, including collagen type 2α1 (Col2α1) and SOX9, as well as collagen-modifying enzymes associated with hindering cartilage-like matrix degradation, including P4HA1 and PLOD2 (Fig. S13F-G). CytoTRACE is commonly used to evaluate the proliferative and differentiative capacity of cells. The results showed that MPC-G associated subsets 5 and 7 not only exhibited a strong capacity to promote cartilage-like matrix accumulation but also demonstrated enhanced proliferative potential, suggesting their critical role in the acquisition of cartilage-like features in tendon tissue (Fig. S13E, H). SCENIC analysis identified HIF-1α as critical regulons for these cells (Fig. 4g). Inhibition of HIF-1α with PX-478 in a hypoxic cellular model abolished the upregulation of matrix degradation-hindering PLOD2 and P4HA1 (Fig. 4h, i; Fig. S14C), cartilage-like matrix synthesis‑related SOX9 and COL2α1 (Fig. S14B), as well as the accumulation of cartilage‑like matrix (Fig. S14D, E). Hypoxia-induced co-expression of HIF-1α and SOX9 was validated by immunofluorescence (Fig. 4j). To validate HIF-1α‘s role, we used SCX^CreERT2^;HIF-1α^flox/flox^ mice (HIF-1α^fibro-^), conditionally knocking out HIF-1α in tenocytes (Fig. 4k). In a collagenase-induced MPC-G model, HIF-1α^fibro-^ mouse showed significant improvements in collagen fiber arrangement (Fig. 4m–o; Fig. S14F-G). Collectively, MPC-G development is associated with the formation of distinct,chondro-modulatory tenocytes driven by hypoxic signaling. HIF-1α acts as a master regulator, mediating the expression of enzymes involved in hindering cartilage-like matrix degradation and genes related to cartilage-like matrix synthesis.

### Lactate as a pathogenic metabolite driving MPC-G tendinopathy via HIF-1α stabilization

**Fig. 5:**
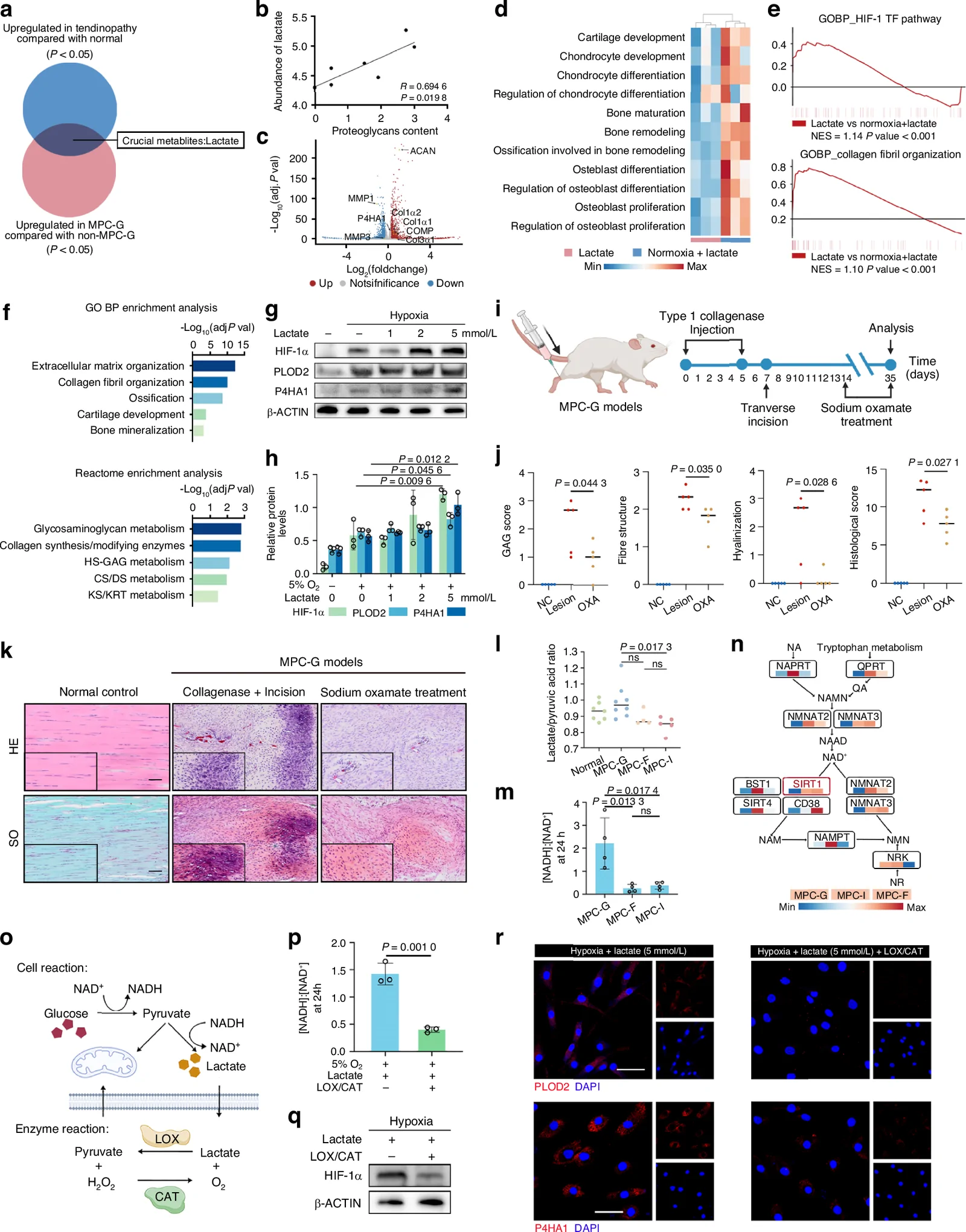
Metabolic intervention via lactate clearance alleviates MPC-G tendinopathy. **a** Screening criteria of cruical pathogenic metabolites of MPC-G. **b** Scatter plot of GAG score versus lactate abundance. **c** Volcano plots of differentially expressed genes between lactate treatment and control groups. **d** GSVA reveals differential chondrification and ossification related pathways between lactate treatment and control groups. **e** GSEA plots showing upregulation of HIF-1 transcription factor pathway and collagen fibril organization pathway in lactate treatment group. **f** GO (Up) and Reactome (Down) enrichment analysis for differentially expressed metabolic genes. **g**, **h** Western blot and semi-quantification of *HIF-1α*, PLOD2 and P4HA1 under 5% hypoxia with increasing lactate. **i** Schematic of oxamate treatment in MPC-G rat model induced by type 1 collagenase. **j** Effect of oxamate on GAG score, fiber structure, hyalinization, and histological score (*n* = 5) **k** Representative H&E and S.O. staining. Scale bar, 100 μm. **l**, **m** The lactate/pyruvate and NADH/NAD^+^ (*n* = 4) ratios are significantly elevated in MPC-G. **n** Expression changes of NAD^+^ synthetases among three MPCs. Alteration scores are log_2_(fold-change versus normal) **o** Schematic illustration of lactate clearance mechanism using LOX/CAT. **p** LOX/CAT alleviating NADH/NAD^+^ imbalance in vitro under 5% hypoxia (*n* = 3) **q** Western blot images showing LOX/CAT inhibiting *HIF-1α* protein accumulation. **r** LOX/CAT inhibiting *P4HA1* and *PLOD2* protein expression. Scale bar, 50 μm (*n* = 3) The data were presented as the mean ± SD. Statistically significant differences are indicated by *P* < 0.05 between the indicated groups

Cross-screening of upregulated metabolites in diseased and MPC-G tissues identified glycolytic product lactate as a key candidate (Fig. 5a, b). Consistently, chondro-modulatory tenocytes exhibited elevated glycolytic activity (Fig. S15A). In line with previous reports, inhibition of HIF-1α significantly downregulated the expression of glycolysis-related genes (Fig. S15B). However, whether lactate stabilizes HIF-1α and synergizes with hypoxic conditions to drive tendinopathy remains to be elucidated. To explore this, we first assessed the effect of lactate under normoxia (21% O_2_) in tenocytes. Lactate treatment upregulated the expression of extracellular matrix genes, including COMP and aggrecan (ACAN), as well as collagen-modifying enzymes such as P4HA1 (Fig. 5c). Upregulated differentially expressed genes were significantly enriched in pathways related to extracellular matrix assembly, ossification, cartilage development, and polysaccharide metabolism (Fig. 5f). The activity of pathways related to chondrification and ossification was significantly increased (Fig. 5d). Notably, the HIF-1α signaling pathway was markedly upregulated (Fig. 5e). Having established that lactate alone can activate HIF-1α-related programs under normoxia, we next asked whether lactate amplifies the cellular response to hypoxia. Under hypoxic conditions (5% O_2_), lactate dose-dependently increased HIF-1α protein levels and upregulated the expression of its downstream targets, including collagen-modifying enzymes (Fig. 5g, h). To determine whether endogenous lactate contributes to MPC-G tendinopathy, we performed loss-of-function experiments using the glycolysis inhibitor sodium oxamate. In vitro, oxamate treatment effectively suppressed the expression of cartilage-like matrix synthesis-related genes at both mRNA and protein level (Fig. S15D-H). In vivo, local injection of oxamate significantly improved GAG score, fiber structure, hyalinization, and histological score in the MPC-G rat model induced by type 1 collagenase combined with transverse incision (Fig. 5i, j), and significantly alleviated the accumulation of cartilage-like matrix (Fig. 5k). Oxamate also significantly ameliorated the thickened appearance of the Achilles tendon (Fig. S15I-K). Previous studies reported that an elevated NADH:NAD^+^ ratio inhibits SIRT1 deacetylase activity. This impedes HIF-1α deacetylation at Lys674, thereby promoting HIF-1α binding to p300 and activating downstream gene expression.^19^ The lactate:pyruvate ratio was commonly used as a marker for the intracellular NADH:NAD^+^ ratio.^20,21^ As shown in Fig. 5l, the lactate:pyruvate ratio was significantly elevated in MPC-G tendinopathy. Further kit-based assays confirmed an increase in the NADH:NAD^+^ ratio in MPC-G tendinopathy (Fig. 5m). The transcriptomic data revealed that NAD^+^-related synthetic and catabolic enzymes, particularly SIRT1 expression, were significantly downregulated in MPC-G tendinopathy (Fig. 5n). To therapeutically target lactate, we used lactate oxidase (LOX) and catalase (CAT) to convert extracellular lactate to pyruvate, thereby detoxifying H_2_O_2_ and restoring NADH/NAD^+^ balance (Fig. 5o). This intervention alleviated lactate-induced HIF-1α accumulation and reduced expression of SOX9 and collagen-modifying enzymes in tenocytes under hypoxia (Fig. 5q, r; Fig. S15L-M).

Overall, our findings demonstrated that glycolytic product lactate served as a critical pathogenic metabolite in MPC-G tendinopathy, promoting disease progression by disrupting NADH/NAD^+^ homeostasis to induce HIF-1α stabilization. Targeted lactate clearance reduced HIF-1α protein accumulation and downregulated genes involved in both cartilage-like matrix synthesis and matrix degradation resistance.

### Targeting HIF-1α to inhibit chondro-modulatory tenocyte formation for MPC-G tendinopathy treatment

**Fig. 6:**
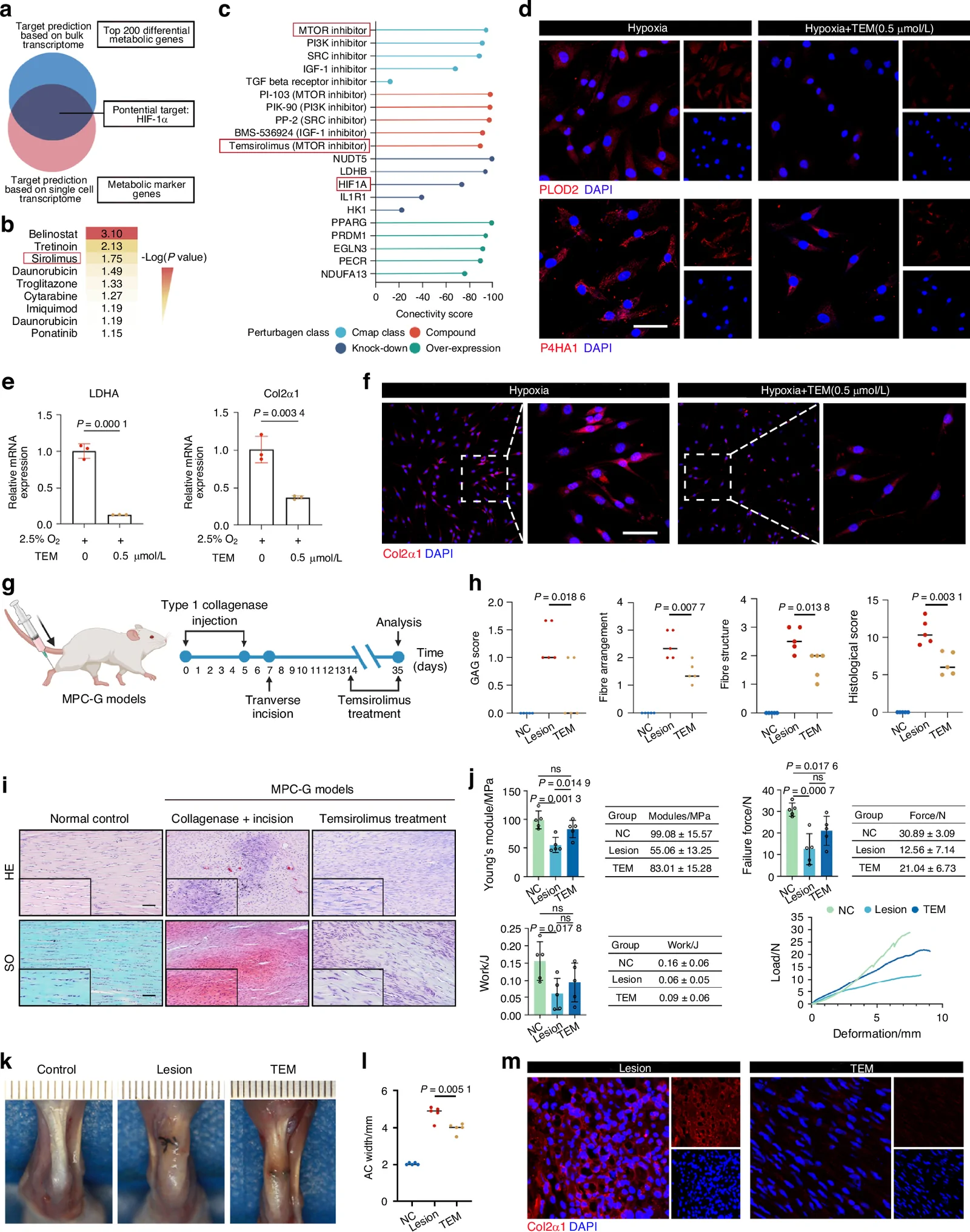
Pharmacological targeting of HIF-1α with temsirolimus ameliorates MPC-G tendinopathy. **a** Screening criteria of a crucial target for MPC-G. **b** Asgard analysis predicting potential therapeutic agents for reversing MPC-G pathogenic cells, based on the metabolic marker gene of these subsets (*P* < 0.05, FC > 2) **c** Cmap analysis predi**c**ting potential therapeutic agents for reversing MPC-G metabolic dysregulation, based on the top 200 overlapping differentially expressed metabolic genes with higher expression in MPC-G compared to other classifications or normal tendons. **d** Immunofluorescence showing effect of temsirolimus on *PLOD2* and *P4HA1* expression under 2.5% hypoxia. Scale bar, 50 μm. **e** Effects of temsirolimus on the *LDHA* and *Col2α1* mRNA expression under 2.5% hypoxia. **f** Immunofluorescence showing the effect of temsirolimus on the protein expression of *Col2α1* under 2.5% hypoxia. Scale bar, 50 μm. **g** Schematic of experiment for temsirolimus treatment in MPC-G rat models induced by type 1 collagenase combined with transverse incision. **h** Effect of temsirolimus on GAG score, fiber structure, fiber arrangement, and histological score (*n* = 5) **i** Representative H&E and S.O. staining. Scale bar, 100 μm. **j** Effect of temsirolimus on histological total score, failure force, work, and load-deformation curve (*n* = 5) **k**, **l** Effect of temsirolimus on Achilles tendon width (*n* = 5) **m** Immunofluorescence showing effect of temsirolimus on *Col2α1* protein expression. Scale bar, 50 μm. The data were presented as the mean ± SD. Statistically significant differences are indicated by *P* < 0.05 between the indicated groups

Finally, we predicted high-potential drugs for MPC-G and MPC-G pathogenic cells based on bulk transcriptome data and single-cell transcriptome data, respectively. For the bulk transcriptome data, we identified overlapping differentially expressed metabolic genes with higher expression in MPC-G than in normal tendons or other metabolic classifications, and utilized the Cmap database for prediction. (Fig. 6a) Consistent with previous findings, HIF-1α was predicted to be a key therapeutic target. In addition, mTOR inhibitors, which suppress HIF-1α accumulation, were identified as high-potential therapeutic agents. For the single-cell transcriptome data, we selected metabolic marker genes (*P* < 0.05 and FC > 2) specific to the MPC-G pathogenic cells and performed prediction using Asgard analysis. (Fig. 6b) The results similarly indicated that mTOR inhibitors possess high therapeutic potential.

mTOR is an atypical serine/threonine protein kinase and a member of the phosphoinositide kinase family. It forms two complexes through binding with different proteins: the rapamycin-sensitive complex mTORC1 and the rapamycin-insensitive complex mTORC2. mTORC1 and mTORC2 promote the expression of downstream HIF-1α by phosphorylating the ribosomal protein S6 kinase (p70S6K) and the eukaryotic translation initiation factor 4E (eIF-4E) binding protein 1 (bp1).^22,23^ However, some studies have reported that mTORC1 is also involved in tendon collagen synthesis, acting as a key effector protein downstream of insulin-like growth factor (IGF) -1 and transforming growth factor (TGF) -β.^24^ In our study, the mTOR inhibitor temsirolimus achieved a high reversal score, thus we evaluated its therapeutic effects. In vitro, it alleviated hypoxia-induced expression of HIF-1α, glycolytic related genes, cartilage-like matrix synthesis-related genes, and collagen-modifying enzymes (Fig. 6d–f; Fig. S16A-B). In a collagenase-induced MPC-G rat model, it reduced proteoglycan accumulation, and histological scores (Fig. S16C-I). Finally, in an MPC-G rat model induced by type 1 collagenase combined with transverse incision, temsirolimus administration improved fiber arrangement, reduced proteoglycan accumulation and Young’s modulus, without compromising failure force (Fig. 6g-n). Immunofluorescence also showed a decrease in the expression of Col2α1 (Fig. 6m).

For MPC-I, Cmap analysis predicted knockdown of inflammatory mediators, such as TNF and IL-6, and agents like cyclooxygenase inhibitors or glucocorticoid receptor agonists as therapeutic strategies (Fig. S17A). To validate the predicted therapeutic strategies for the MPC-I subtype, we established a levofloxacin-induced MPC-I rat model and assessed the efficacy of glucocorticoid treatment. Histological analysis revealed that glucocorticoid treatment significantly improved tendon architecture, reducing abnormal vascularization and cellularity compared to the untreated MPC-I model group (Fig. S17C, D). Transcriptomic analysis further demonstrated that glucocorticoid treatment restored the expression of genes involved in fatty acid oxidation, which was downregulated in the MPC-I model (Fig. S17B). Consistently, the treatment also suppressed the expression of key genes associated with MMPs and the VEGF signaling pathway (Fig. S17E). These results demonstrate that glucocorticoid treatment effectively alleviates key pathological features of MPC-I tendinopathy by targeting inflammation and its downstream consequences. Overall, our findings expand precision therapy for tendinopathy: temsirolimus effectively targets the mTOR/HIF-1α axis in MPC-G, inhibiting pathological accumulation of cartilage-like matrix while preserving mechanical function, and glucocorticoids show efficacy in MPC-I by restoring metabolic homeostasis and reducing inflammation.

## Discussion

Dysregulated metabolism was previously reported to be associated with the progression of tendinopathy,^25–27^ and regulation of metabolism is a powerful strategy for the treatment of tendinopathy.^28,29^ Understanding the metabolic heterogeneity of diseases is crucial to elucidating the pathogenesis and developing therapeutic approaches for heterogeneous diseases such as tendinopathy. We successfully classified tendinopathy into 3 heterogeneous metabolic classifications with distinct metabolic characteristics, histological changes, and clinical function. Targeting the biosynthesis of some functional metabolites, such as lactate, or using mTOR inhibitors, such as temsirolimus, both improved MPC-G tendinopathy by inhibiting HIF-1α accumulation. Anti-inflammatory drugs, such as glucocorticoid, improved fatty acid oxidation levels and abnormal vascularization in MPC-I tendinopathy.

We observed common patterns of metabolic adaptation shared across all MPCs, including increased glucose utilization and glycan synthesis, and decreased lipid oxidation energy supply. The previous studies also reported a significant shift in the energy metabolism patterns of diseased tendon. Kvist et al. were the first to report an increase in the activity of anaerobic glycolytic enzymes and pentose phosphate pathway enzymes in tendinopathy, while the activity of aerobic energy metabolism enzymes decreased.^30^ Zhang et al.‘s research showed that injured tendons exhibit increased glucose utilization, with enhanced glycolysis, lactate synthesis, and tricarboxylic acid cycle activity.^31^ Besides, we also found that vitamin/cofactor and amino acid metabolism show significant dysregulation in the diseased tendon. Previous studies have reported that vitamin D and vitamin C supplementation may help with tendinopathy recovery.^32,33^ Overall, our study provides the first holistic landscape of metabolic alterations in the tendons of disease. Among three MPCs, MPC-F exhibited the mildest metabolic dysregulation and histological lesions, indicating a less severe pathological state. This classification had high levels of fatty acid oxidation and amino acid metabolism similar to normal tendons. MPC-G exhibited significant metabolic features in hypoxic environments, including activation of glycolysis, which were histologically manifested as decreased cell density and increased cartilage-like matrix accumulation. MPC-I showed intermediate levels of glycolysis and fatty acid oxidation in the transcriptome, but exhibited the most significant metabolite dysregulation of all MPCs in metabolome. This classification showed severe vascular infiltration and a higher degree of joint adhesion.

Clinical parameters showed distinct patterns across the three MPCs. Among the routine demographic and comorbidity parameters, only BMI showed a statistically significant difference. MPC-F had the highest proportion of normal-BMI patients whereas MPC-G and MPC-I trended toward higher BMI values. Hematological parameters also varied across groups. The albumin/globulin ratio was lowest in MPC-I. Given that alanine aminotransferase, aspartate aminotransferase and bilirubin levels were normal across groups, this reduction is unlikely due to hepatic synthetic dysfunction and may instead reflect differences in nutritional status or systemic metabolism. Additionally, MPC-I showed a higher proportion of glycated albumin, and MPC-G showed longer thrombin time. These observations hint at systemic metabolic or nutritional influences on local tendon phenotypes. Although cross-sectional data cannot establish causality, we speculate that BMI may predispose individuals to specific metabolic classifications. Elevated BMI is commonly associated with systemic metabolic alterations including insulin resistance and dyslipidemia as well as chronic low-grade inflammation. These changes could influence the local tendon microenvironment and favor distinct metabolic phenotypes.^34^ For instance the inflammatory characteristics of MPC-I may be exacerbated by obesity-related adipose tissue dysfunction and increased circulating pro-inflammatory cytokines. Conversely, it is also possible that the metabolic classification reflects an underlying systemic metabolic state with BMI serving as a surrogate marker rather than a direct driver. Longitudinal studies are needed to clarify whether BMI modulation could shift metabolic subtype assignment or alter disease progression. Integrating systemic metabolic parameters with local tissue profiling may refine patient stratification in future studies. We further investigated the drivers for each MPC. MPC-F showed activation of HIF-1α pathway; MPC-I showed activation of NF-kB pathways; MPC-G showed activation of both HIF-1α and NF-kB pathways. Our research showed that the critical signals for development of MPC-I may be TNF-α and IL-1β, while the critical signals for development of MPC-G may be HIF-1α and IL-1β. The activation of HIF-1α and IL-1β pathways inducing a shift in the energy metabolism pattern of fibroblasts from predominantly utilizing fatty acids to mainly utilizing glucose.

Previous studies have reported that inflammation and oxidative stress are strongly associated with ectopic ossification of tendons.^35–37^ Our research deepened these understandings. Specifically, we found that IL-1β stimulation and H_2_O_2_-mediated oxidative stress promoted chondrogenesis, while TNF-α stimulation did not. Similarly, we observed that hypoxia contributed to this process by upregulating SOX9, a transcription factor linked to chondrogenesis that is typically elevated under pathological conditions. Notably, IL-1β stimulation and H_2_O_2_-mediated oxidative stress significantly induced the expression of multiple extracellular matrix degrading enzymes. This may be the reason why MPC-I did not develop cartilage-like matrix accumulation. Hypoxia significantly induced expression of multiple collagen crosslinking-related collagen-modifying enzymes. Previous studies have reported that the upregulation of these enzymes enhanced the ability of cartilage-like matrix to resist degradation.^38–40^ In tendinopathy, hypoxia is considered an initial signal triggering extracellular matrix disruption and tendon degeneration.^41^ The study by Jiao et al. found that inhibiting HIF-1α effectively reduced the phosphorylation levels of p65 in the NF-kB pathway, as well as the phosphorylation levels of extracellular signal-regulated kinase 1/2 (ERK1/2), p38, and Jun N-terminal kinase (JNK) in the MAPK pathway.^42^ Additionally, excessive activation of HIF-1α signaling exacerbates scar formation by inducing aberrant angiogenesis.^43^ However, hypoxia also plays a beneficial role in tendon repair. For example, hypoxia promotes the proliferation and migration of tendon stem cells and enhances the expression of tendon-related markers such as Col1α1, Col3α1, decorin (DCN), and tenomodulin.^44^ Future studies should aim to balance the dual effects of hypoxia and develop targeted therapeutic strategies. We further investigated the pathogenesis of cells for each MPC. MPC-I tendinopathy was related to mural cells, and MPC-G tendinopathy was related to chondro-modulatory tenocytes. Both hypoxia-related HIF-1α and inflammation-related NF-kB signaling activation were associated with the formation of chondro-modulatory tenocytes, but HIF-1α may be the key regulator. HIF-1α mediated the transformation of fibroblasts into chondro-modulatory tenocytes through SOX9 expression induced by glycolytic reprogramming. Previous studies have found a close association between tendon chondroid metaplasia and mucoid degeneration.^45,46^ Additionally, tendon calcification often coexists with chondroid metaplasia.^45^ Recently, Fu et al. revealed a continuous process in which tendon stem cells transform into pathological fibroblasts, chondrocytes, and osteocytes under tendinopathy conditions, highlighting the critical driving role of inflammation in this process.^47^ Metabolic alterations also play a significant role in chondrocyte formation. In a prior study, Izumi et al. reported that the glycolysis inhibitor 2-DG significantly suppressed the expression of chondrogenic marker genes, including ACAN and SOX9, in micro-mass-cultured tendon-derived progenitor cells, which is consistent with our findings.^48^

Finally, we explored classification-specific treatment strategies. We found that lactate was an important MPC-G-promoting metabolite, which enhanced the accumulation of HIF-1α protein. Tendons are avascular tissues with physiological oxygen levels estimated between 1% and 5%.^49–51^ We therefore conducted in vitro studies at 5% O_2_ to evaluate lactate’s pathogenic role in disease progression. Under these conditions, lactate significantly stabilized HIF-1α protein accumulation. The mechanisms by which lactate regulates HIF-1α are multifaceted. Studies under normoxic conditions have reported that lactate stabilizes HIF-1α by inhibiting prolyl hydroxylase expression^52^ or via lactylation modification.^53^ However, the effect of lactate on HIF-1α accumulation is highly dependent on oxygen concentration. Liu et al. demonstrated that under a more severe 1% O_2_ environment, lactate conversely promotes the degradation of HIF-1α protein.^54^ This contrast underscores that the lactate-HIF-1α feedback loop operates most potently within a specific oxygen niche.

mTOR inhibitors were high-potential agents for MPC-G tendinopathy. Previous studies have found that mTORC1 signaling was a key signal for normal tendon development and maturation.^55^ However, an increasing body of research suggested that abnormal activation of mTOR signaling under disease conditions may be associated with ectopic ossification in tendons.^56,57^ In this study, we found that temsirolimus, a widely used mTOR inhibitor for treating various diseases, effectively inhibited glycolysis and chondro-modulatory tenocyte formation in MPC-G tendinopathy. In the previous process of analyzing transcriptome heterogeneity, we identified two transcriptional subtypes: subtype H and subtype I, where no effective treatment strategy has been developed for subtype H.^14^ Metabolic MPC-G highly overlaped with transcriptional subtype H, and our study filled in the gaps of previous studies. Inflammation inhibition was the critical target for treatment of MPC-I tendinopathy, and glucocorticoid effectively restored fatty acid oxidation levels and inhibited vascularization in MPC-I. Overall, our research provides therapeutic strategies with strong clinical translational potential for each MPC. Offering different treatment strategies based on metabolic characteristics for patients with different tendinopathy MPCs may be clinically beneficial.

Several limitations of our study should be considered. First, using hamstring tendons as controls, while ethically necessary, introduces anatomical differences. However, prior studies have demonstrated cellular similarity between these tendon types.^58^ Importantly, the identification of three metabolic-pathway-based classifications was derived from unsupervised clustering within the diseased cohort itself, with normal controls serving only to establish a baseline; thus, the observed metabolic heterogeneity is robust to the choice of control tissue. Second, the activity of metabolic pathways indirectly reflects the metabolic state. Therefore, we performed pseudo-targeted metabolomics on tendinopathy samples to validate our results. Secondly, the efficacy of the identified classification-specific drugs still requires further clinical evaluation.

In future studies, it is essential to conduct further multi-omics analysis on different tissue types within the rotator cuff region, including adipose, muscle, and synovial bursa tissues, to comprehensively elucidate the complex microenvironment of rotator cuff tendinopathy, which is crucial for clarifying the pathogenesis of metabolic classification. In particular, integrating proteomic data will be critical to validate and refine the transcriptome-based metabolic subtypes, as it directly reflects the functional state of metabolic enzymes and pathways. During the translation of metabolic classification into clinical practice, several key challenges remain. First, preoperative diagnosis of metabolic subtypes requires the identification of reliable biomarkers to prospectively guide treatment selection. Potential candidates may include circulating metabolites detectable via serum metabolomics, or imaging-based radiomic signatures. Beyond biomarker discovery, the development of rapid diagnostic tools, such as preoperative prediction models or intraoperative diagnostic models integrating multi-modal data, that are compatible with routine clinical workflows will be essential for clinical implementation. Second, further large animal studies are needed to evaluate the efficacy of subtype-specific therapeutic strategies. Meeting these challenges will be essential to realize the promise of precision medicine for patients with tendinopathy.

In conclusion, our study revealed the metabolic heterogeneity in tendinopathy and identified three classifications with distinct metabolic phenotypes. Hypoxia and inflammation determined the metabolic fate of tendinopathy. Different metabolic classifications were suitable for different therapeutic strategies. MPC-I tendinopathy was related to mural cells, and MPC-G tendinopathy was related to chondro-modulatory tenocytes. For MPC-G, we revealed that lactate was a crucial promoting metabolite and mTOR inhibitor temsirolimus effectively improved this classifcation by inhibiting HIF-1α; For MPC-I, we revealed that glucocorticoid effectively improved this classifcation by inhibiting inflammation.

## Materials and methods

### Study cohorts

All human tissue samples included in the study were obtained after approval of the local ethics committee (Ethics Committee of the Second Affiliated Hospital, School of Medicine, Zhejiang University, code: 2019-168, 2020-080, 2023-1175). The patients with supraspinatus tendon injuries who met the criteria and were willing to participate in the present study were included as the disease group. The inclusion criteria were patients with confirmed supraspinatus tendon injuries based on MRI, with tear lengths less than 5 cm, and exclusion criteria were tear lengths larger than 5 cm. The wasted tendon from shoulder arthroscopic surgery were collected (*n* = 197). The patients with anterior cruciate ligament injury and were willing to participate in the present study were included as the normal group. The wasted healthy hamstring tendons were collected (*n* = 32). In all, BulkRNA-sequencing was performed on 192 diseased tendons and 29 normal tendons. Among these, 17 diseased tendons and 17 normal tendons also underwent pseudotargeted metabolome analysis Additionally, five diseased tendons and three normal tendons were subjected to scRNA-sequencing analysis only.

### Rat

All animal experiments were performed according to protocols approved by the Research Ethical Committee of Zhejiang University Institutional Animal Care and Use Committee (ZJU20240137).

Eight- to ten-week-old male Sprague-Dawley adult rats with a body weight ranging from 230 to 270 g were used in this study. Rats were housed under 12 h light/darkness cycles and ambient temperature (20–22)°C and humidity (60% ± 10%) with free access to standard rodent diet and water ad libitum in individually ventilated cages.

### Mice

All animal experiments were performed according to protocols approved by the Research Ethical Committee of Zhejiang University Institutional Animal Care and Use Committee (ZJU20230128 and ZJU20240137).

All used mouse strains were immunocompetent and housed in standard cages under 12 h light/darkness cycles and ambient temperature (20–22) °C and humidity (60% ± 10%) with free access to standard rodent diet and water ad libitum in individually ventilated cages. SCX^creERT2^ (NM-KI-215092) mice and HIF-1α^fl/fl^ (NM-CKO190065) mice were obtained from the Shanghai Model Organisms Center. Tendon fibroblast-specific deletion of HIF-1α was obtained by crossing these mouse (SCX^creERT2+^; HIF-1α^fl/fl^, referred to as HIF-1α^Fibro−^) SCX^creERT2-^; HIF-1α^fl/fl^ (referred to as HIF-1α^Fibro+^) littermates were used as controls in all experiments.

### Isolation and culture of healthy human tendon stem/progenitor cells

Fresh healthy hamstring tendon tissues were placed in cold high-glucose DMEM (H-DMEM) (Gibco) with 1% penicillin-streptomycin and transported to the lab in an ice box for fibroblast cell isolation and culture. Tissues were washed in the cold PBS for 3–5 times and then minced into small fragments (1 mm^3^ or less) using sterile scalpels. Tissues were digested with 0.2% type 1 collagenase in H-DMEM for 4 h at 37 °C. Dissociated tissues were spun down at 1 250 g for 5 min and resuspended in H-DMEM with 10% fetal bovine serum (FBS) and spun down again. Cell subsets were lastly cultured in a basic medium (H-DMEM, 10% FBS, and 1% penicillin–streptomycin).

### BulkRNA-sequencing for tendinopathy samples

Total RNA was extracted from the tissue using TRIzol® Reagent according the manufacturer’s instructions (Invitrogen) and genomic DNA was removed using DNase I (TaKara). BulkRNA-sequencing transcriptome libraries were prepared following TruSeqTM RNA sample preparation Kit from Illumina (San Diego, CA), using 1 μg of total RNA. Shortly, messenger RNA was isolated with polyA selection by oligo(dT) beads and fragmented using fragmentation buffer. cDNA synthesis, end repair, A-base addition and ligation of the Illumina-indexed adaptors were performed according to Illumina’s protocol. Libraries were then size-selected for cDNA target fragments of 200–300 bp on 2% Low Range Ultra Agarose, followed by PCR amplified using Phusion DNA polymerase (NEB) for 15 PCR cycles. After quantification by TBS380, Paired-end libraries were sequenced with the Illumina HiSeq PE 2X150 bp read length.

### Reads quality control and mapping

The raw paired-end reads were trimmed and quality controlled by Trimmomatic with default parameters (http://www.usadellab.org/cms/uploads/supplementary/

Trimmomatic). Then clean reads were separately aligned to reference genome with orientation mode using hisat2 (https://ccb.jhu.edu/software/hisat2/index.shtml) software. Tophat was a program which can align RNA-Seq reads to a genome in order to identify gene expression and exon-exon splice junctions. It is built on the ultrafast short read mapping program Bowtie2. This software was used to map with default parameters.

### Metabolite extraction of tendinopathy samples

The sample stored at −80 °C refrigerator was thawed on ice. Add a steel ball with tweezers. A 400 μL solution (Methanol:Water = 7:3, V/V) containing internal standard was added to 20 mg grinded sample, and shaked at 1 500 r/min for 5 min. After placing on ice for 15 min, the sample was centrifuged at 12 000 r/min for 10 min (4 °C). All of supernatant was collected and placed in −20 °C for 30 min. The sample was then centrifuged at 12 000 r/min for 3 min (4 °C). All aliquots of supernatant were transferred for LC-MS analysis.

### LC-MS/MS analysis of tendinopathy samples

All samples were for three LC/MS methods. One aliquot was analyzed under positive ion conditions using a T3 column (Waters ACQUITY UPLC HSS T3 C18 1.8 µm, 2.1 mm * 100 mm). Solvent A was 0.1% formic acid in water, and solvent B was 0.1% formic acid in acetonitrile, with the following gradient: 5%-20% B in 2 min, 20%–60% B in 3 min, 60%–99% B in 1 min, held at 99% B for 1.5 min, then returned to 5% B in 0.1 min and held for 2.4 min. The analytical conditions were as follows: column temperature, 40 °C; flow rate, 0.4 mL/min; injection volume, 2 μL or 5 μL; Another alipuot was using negative ion conditions and was the same as the elution gradient of positive mode. The third alipuot was analyzed via negative ionization and was eluted from a HILIC column (Waters ACQUITY UPLC BEH HILIC Column 1 mm * 150 mm, 1.7 µm) using 60% ACN, 30% water, and 10% MeOH with 20 mmol/L ammonium formate, pH 10.6 as solvent A and 40% ACN and 60% water with 20 mmol/L ammonium formate as solvent B in the following gradient, 5% to 30% B in 3.5 min, 50% to 95% B in 2 min, hold at 95% B for 1 min, then rapidly return to starting conditions. The data acquisition was operated using the information-dependent acquisition (IDA) mode using Analyst TF 1.7.1 Software (Sciex, Concord, ON, Canada). The source parameters were set as follows: ion source gas 1 (GAS1), 50 psi; ion source gas 2 (GAS2), 50 psi; curtain gas (CUR), 25 psi; temperature (TEM), 550°C; desubseting potential (DP), 60 V, or−60 V in positive or negative modes, respectively; and ion spray voltage floating (ISVF), 5 000 V or−4 000 V in positive or negative modes, respectively. The TOF MS scan parameters were set as follows: mass range, 50–1 000 Da; accumulation time, 200 ms; and dynamic background subtract on. The product ion scan parameters were set as follows: mass range, 25–1 000 Da; accumulation time, 40 ms; collision energy, 30 or −30 V in positive or negative modes, respectively; collision energy spread, 15; resolution, UNIT; charge state, 1 to 1; intensity, 100 cps; exclude isotopes within 4 Da; mass tolerance, 50 ppm; maximum number of candidate ions to monitor per cycle, 18. LIT and triple quadrupole (QQQ) scans were acquired on a triple quadrupole-linear ion trap mass spectrometer (QTRAP), QTRAP® LC-MS/MS System, equipped with an ESI Turbo Ion-Spray interface, operating in positive and negative ion mode and controlled by Analyst 1.6.3 software (Sciex). The ESI source operation parameters were as follows: source temperature 500 °C; ion spray voltage (IS) 5 500 V (positive), −4 500 V (negative); ion source gas I (GSI), gas II (GSII), and curtain gas (CUR) were set at 50, 50, and 25.0 psi, respectively; the collision gas (CAD) was high. Instrument tuning and mass calibration were performed with 10 and 100 μmol/L polypropylene glycol solutions in QQQ and LIT modes, respectively. A specific set of MRM transitions was monitored for each period according to the metabolites eluted within this period.

### Single-cell RNA-sequencing for tendinopathy samples

Cellular suspensions were loaded on a 10X Genomics GemCode Single-cell instrument that generates single-cell Gel Bead-In-Emulsion (GEMs). Libraries were generated and sequenced from the cDNAs with Chromium Next GEM Single Cell 3’ Reagent Kits v3.1. Silane magnetic beads were used to remove leftover biochemical reagents and primers from the post GEM reaction mixture. Full-length, barcoded cDNAs were then amplified by PCR to generate sufficient mass for library construction. R1 (read 1 primer sequence) were added to the molecules during GEM incubation. P5, P7, a sample index, and R2 (read 2 primer sequence) were added during library construction via End Repair, A-tailing, Adaptor Ligation, and PCR. The final libraries contained the P5 and P7 primers used in Illumina bridge amplification.

The Single Cell 3’ Protocol produced Illumina-ready sequencing libraries. A Single Cell 3’ Library comprised standard Illumina paired-end constructs which begin and end with P5 and P7. The Single Cell 3’ 16 bp 10x Barcode and 10 bp UMI were encoded in Read 1, while Read 2 was used to sequence the cDNA fragment. Sample index sequences were incorporated as the i7 index read. Read 1 and Read 2 were standard Illumina® sequencing primer sites used in paired-end sequencing.

### Differential gene expression analysis and enrichment analysis

To account for any batch effects, we used removeBatchEffect in the Bioconductor limma package and Bioconductor DESeq2 to normalize the data. We used the DESeq2 package^59^ to identify differentially expressed genes (DEGs). Gene Ontology (GO) and Kyoto Encyclopedia of Genes and Genomes (KEGG) pathway enrichment analysis were performed using DAVID (https://david.ncifcrf.gov/). Gene set enrichment analysis (GSEA).^60^ was completed through GSEA software (http://www.gsea-msigdb.org/gsea/index.jsp).

### Differential Rank Conservation (DIRAC) analysis

The DIRAC analysis^15^ was performed using MATLAB (version R2016a). DIRAC is composed of a set of measures to quantify differential expression variability among phenotypes using subsets of genes based on the transcriptome of individuals. Based on the comparison between the expression values of pairs of genes, it would define a rank template for each pathway and phenotype representing the expected pairwise ordering of gene expression for that pathway in that phenotype. Then, the rank matching score (RMS) was used to determine how well the pathway ranking in each individual sample matched the ordering defined in the rank template. Averaging the RMS over all samples within a phenotype yields a pathway-specific rank conservation index (RCI), which represents how well, on average, all samples in the same phenotype match the corresponding rank template. An RCI of 1.0 indicates the ranks of pathway genes are mostly unchanged among samples, and an RCI of 0.5 indicates the ranks of pathway genes are greatly varied between samples of the same phenotype. In this study, 148 metabolic pathways comprising 2 732 human metabolic genes from KEGG and REACTOME were applied to calculate the RCIs in normal or tendinopathy samples.

### Metabolic-gene-based subseting

The top 50% most-variant metabolic genes were subjected to non-negative matrix factorization (NMF) for unsupervised consensus-subseting. The non-smooth NMF (nsNMF) and the Brunet algorithm was performed using 200 iterations for the rank survey and subseting runs.^61,62^ A preferred subset result was selected by considering the profiles of the cophenetic score and average silhouette width for subseting solutions between 2 and 7 subsets.

### Calculation of metabolic pathway enrichment score

Metabolic pathways were downloaded from the Kyoto Encyclopedia of Genes and Genomes (KEGG) database (https://www.genome.jp/kegg/) and Reactome database (https://reactome.org/). A total of 2 732 human metabolic genes from each of the 148 metabolic pathways are listed in Table S2, 3. Gene set variation analysis (GSVA) was utilized to calculate the enrichment score of each metabolic pathway in each sample with transcriptomic data.^16^

### Metabolic-pathway-based subseting

The nsNMF and the Brunet algorithm were performed to determine the metabolic pathway-based tendinopathy classifications. In order to meet the requirements of nsNMF and the Brunet algorithm, we transform the matrix of metabolic pathway enrichment score. The specific method is to construct the positive and negative matrix of metabolic pathway enrichment score. A positive matrix transforms a negative number to 0, while a negative matrix transforms a positive number to 0 and takes the absolute of a negative number. Finally, combine the two matrices. A preferred subset result was selected by considering the profiles of the cophenetic score and average silhouette width for subseting solutions between 2 and 7 subsets.

### Integration of single-cell data across patients and assessment of batch effects

To integrate single-cell transcriptomic data from multiple donors and minimize technical batch effects, we utilized the anchor-based integration workflow implemented in Seurat v3.^63^ Briefly, we first performed log-normalization and identified highly variable features in each dataset independently. Datasets were then integrated using the FindIntegrationAnchors and IntegrateData functions with default parameters, which identify mutual nearest neighbors across datasets in a shared low-dimensional space (canonical correlation vectors) and compute a corrected expression matrix for joint analysis. After integration, UMAP projections demonstrated that cells from different donors were well mixed and clustered primarily by biological cell types rather than batch origin. This confirmed effective removal of batch effects while preserving biological heterogeneity. All downstream analyses, including clustering, dimensionality reduction, and differential expression, were performed on the integrated data.

### Association of single-cell clusters with bulk-derived metabolic classifications

To associate single-cell clusters with the bulk-derived MPCs, we first identified MPC-specific gene signatures. Using the DESeq2 package, we performed differential expression analysis on the bulk RNA-seq data, comparing each MPC group (e.g., MPC-G) against all other disease samples (MPC-F and MPC-I combined). Metabolic genes with an adjusted *P*-value < 0.05 were selected and ranked by log_2_ fold change. The top 25% of upregulated metabolic genes for each MPC were defined as its specific signature. The activity of these signatures in each single cell was then quantified using the AddModuleScore_UCell function from the UCell package, which calculates a score based on the average expression of the signature genes relative to a background of randomly selected genes.

### Prediction of metabolic-pathway-based classification for scRNA-seq donors

To assign MPCs to additional tendon samples derived from scRNA-seq, we developed a random forest classifier based on the bulk RNA-seq data of the 192 tendinopathy samples with established MPCs. For each donor subjected to scRNA-seq, a pseudo-bulk expression profile was generated by aggregating the transcript counts of all cells from that donor. The training dataset consisted of the bulk gene expression matrix and the corresponding classification labels. For each pseudo-bulk sample, the expression matrix was filtered to retain only genes present in the training set. Both training and pseudo-bulk matrices were then transposed to a sample-by-gene format and normalized by Z-score transformation using the mean and standard deviation of each gene calculated from the training set. A random forest model was trained using the randomForest R package with 500 trees and default parameters, using the normalized training data as predictors and the classification as the outcome. The trained model was subsequently applied to the normalized expression data of the pseudo-bulk samples to predict their MPCs, yielding both a predicted class and posterior probabilities for each of the three MPCs. This approach leverages the full transcriptomic signature to classify each scRNA-seq donor into the previously established metabolic framework.

### Validation of MPCs mechanisms using animal models

To evaluate the impact of inflammation and hypoxia-related signaling pathway activation on tendon tissue, we established tendinopathy animal models using different methods in 8-week-old male Sprague-Dawley rats and compared the differences among these models. A total of 5 rats were included as the normal control group. 25 rats were assigned to type 1 collagenase injection group, PGE2 injection group, levofloxacin injection group, tendon ligation group and forced treadmill running group. After 4 weeks of modeling, rats were euthanized with excess pentobarbital sodium, and tendon samples were collected for transcriptome sequencing and histological evaluation. The specific scheme of animal model construction was as follows. Type 1 collagenase (0.2%, 30 U per leg) was injected into the middle of the Achilles tendon and the same operation repeated 3 days later to stabilize the effect in order to construct the type 1 collagenase injection model. PGE2 (0.002%, 30 U per leg) was injected into the middle of the Achilles tendon and the same operation MNN 3 days later to stabilize the effect in order to construct the PGE2 injection model. Levofloxacin (0.2%, 30 U per leg) was injected into the middle of the Achilles tendon and the same operation repeated 3 days later to stabilize the effect in order to construct the levofloxacin injection model. For the tendon ligation model, a 3 cm incision was made directly above the Achilles tendon, and the tendon was isolated from the surrounding fascia. Finally, the myotendinous portion and calcaneal insertion were ligated with natural silk sutures. For forced treadmill running model, rats were subjected to treadmill activity under modified conditions (20 m/min, 6 days/week, 0°) for 1 h/day.

### Validation of MPCs mechanisms using cell models

To evaluate the impact of hypoxia, oxidative stress and different proinflammatory cytokines on tendon-derived fibroblasts, we established tendinopathy cell models using different methods and compared the differences among these models. Healthy human tendon-derived fibroblasts were treated with IL-1β (5 ng/mL), TNF-α (5 ng/mL), IL-1β (5 ng/mL)+TNF-α (5 ng/mL), H_2_O_2_ (200 μmol/L), respectively, or cultured them in 2.5% hypoxic environment for 24 h. Three replicates were set for each treatment group. The total RNA was then extracted for transcriptome sequencing.

### The effect of tendon fibroblast-specific knockout of HIF-1α on tendon tissue

In the study evaluating the effect of tendon fibroblast-specific knockout of HIF-1α on cartilage-like matrix accumulation, three SCX^creERT2+^; HIF-1α^fl/fl^ mice were assigned to the knockout group, and three SCX^creERT2-^; HIF-1α^fl/fl^ mice served as controls. Collagenase type I (1.0%, 30 U per leg) was injected into the midportion of the Achilles tendon in both groups and repeated after 3 days to simulate MPC-G tendinopathy.

For the knockout group: Tamoxifen was dissolved in corn oil at a concentration of 20 mg/mL by shaking the solution for at least 4 h at 37 °C in the dark. HIF-1α knockout induction: 8-week-old mice received daily intraperitoneal (i.p.) injections of 100 μg/g tamoxifen (Sigma, T5648) dissolved in corn oil to induce CreERT2-mediated knockout. The same procedure was performed in the control group.

After 4 weeks of treatment, mice were euthanized via pentobarbital sodium overdose, and tendon samples were collected for further evaluation.

### In vivo trials of glucocorticoid treatment

We evaluated the response to glucocorticoid treatment in the representative MPC-I tendinopathy model induced by levofloxacin. A total of 15 rats were randomly allocated to the normal control group, levofloxacin group and levofloxacin+glucocorticoid group. After 2 week of modeling, rats in levofloxacin+glucocorticoid group were local injected with 30 μL of compound betamethasone(diprospan) peritendinous, while rats in levofloxacin group were local injected with saline. After 2 weeks of treatment, rats were euthanized with excess pentobarbital sodium, and tendon samples were collected for histological evaluation and bulk RNA-sequencing.

### In vivo trials of temsirolimus treatment

The in vivo trials of temsirolimus treatment were divided into two parts. First, we explored the response of representative MPC-G tendinopathy models to temsirolimus treatment. Secondly, to more closely mimic clinical scenarios, we introduced lesion repair into the representative animal models and re-evaluated the efficacy of temsirolimus treatment on the animal models. The subsequent paragraph describes the consistent experimental process for two parts of an in vivo trial. The lesion repair model was constructed as follows: Type 1 collagenase (0.2%, 30 U per leg) was injected into the middle of the Achilles tendon and the same operation repaeted 3 days later to stabilize the effect. Surgical procedures were performed under general anesthesia (intraperitoneal injection of 40 mg/kg pentobarbital sodium) 7 days after the initial collagenase injection. The rats were prevented from feeling any pain by controlling their reflexes at intervals throughout the procedure and by administering an additional dose of anesthesia when necessary. After shaving, the incision areas were washed with povidone-iodine and all surgical procedures were performed under sterile conditions. After the surgical preparation of the lower extremities, ankle posterior line incisions were made and the Achilles tendons and plantar tendons were exposed. 0.2 cm partial lacerations were performed at 0.5 cm proximal to the calcaneus insertion of the middle of the Achilles tendons, by using a flat blade with a width of 0.2 cm. Tendons were repaired end-to-end with 6/0 round polypropylene monofilament sutures. After washing the wounded areas with saline, the incisions were closed by using 4/0 polypropylene monofilament sutures and the integrity of the skin was achieved. In the first part, 15 rats were allocated to the normal control group, collagenase group and collagenase+temsirolimus group. After 1 week of modeling, rats in collagenase + temsirolimus group were local injected with 30 μL of temsirolimus (100 μmol/L), while rats in collagenase group were local injected with saline. Tendon samples were subjected to histological evaluation following euthanasia with excess pentobarbital sodium at 3 weeks post-treatment.

In the second part, 15 rats were allocated to the normal control group, lesion group and lesion + temsirolimus group. After 1 week of laceration, rats in lesion + temsirolimus group were local injected with 30 μL of temsirolimus (100 μmol/L), while rats in collagenase group were locally injected with saline. Tendon samples were subjected to histological evaluation following euthanasia with excess pentobarbital sodium at 3 weeks post-treatment.

### In vitro trials of PX-478 treatment

Human healthy tendon-derived fibroblasts were cultured under different conditions to evaluate the effect of HIF-1α inhibition. For mRNA expression analysis, cells were treated for 24 h in a hypoxic environment (2.5% O_2_) with or without the HIF-1α inhibitor PX-478 (20 μmol/L), and the expression levels of SOX9 and COL2α1 were assessed. For immunofluorescence staining, cells were similarly cultured under hypoxia (2.5% O_2_) with or without PX-478 (20 μmol/L) for 24 h, and the protein levels of PLOD2 and P4HA1 were examined. For Western blot analysis, cells were subjected to three conditions: normoxia, hypoxia (2.5% O_2_), and hypoxia with PX-478 (20 μmol/L) for 24 h, and the protein expression of SOX9 and PLOD2 was evaluated. Three replicates were set for each treatment group.

### Pellet culture under hypoxic conditions

Human healthy tendon-derived fibroblasts were detached and resuspended at a density of 1 × 10^5^ cells per 10 μL. Twelve-well plates were pre-coated with 1% gelatin at 37 °C for 1 h, air-dried, and then 10 μL of the cell suspension was seeded into each well. After allowing cell attachment for 2 h under normoxia, 1 mL of chondrogenic induction medium (H-DMEM supplemented with 1% ITS, 1 mmol/L sodium pyruvate, 10^−7 ^mol/L dexamethasone, 50 μg/mL ascorbic acid, 10 ng/mL TGF-β3, and 1% penicillin/streptomycin) was added. The micromass cultures were maintained under hypoxia (2.5% O_2_) with or without the HIF-1α inhibitor PX-478 (20 μmol/L) for 14 days, with medium changes every 2–3 days. On day 14, the micromasses were collected for histological analysis.

### In vitro trials of sodium oxamate treatment

In the sodium oxamate treatment experiment, the human healthy tendon-derived fibroblasts were treated for 24 h in a 2.5% hypoxic environment with or without sodium oxamate (20 mmol/L). Three replicates were set for each treatment group.

### In vitro trials of temsirolimus treatment

In the temsirolimus treatment experiment, the human healthy tendon-derived fibroblasts were treated for 24 h in a 2.5% hypoxic environment with or without temsirolimus (0.5 μmol/L). Three replicates were set for each treatment group.

### In vitro trials of lactate effects

The in vivo trials of lactate effects were divided into two parts. First, we evaluated the effects of different concentrations of lactate on tendon-derived fibroblasts. Second, we assessed the role of lactate clearance. In the first part, we initially treated human tendon-derived fibroblasts with or without 5 mmol/L lactate under normoxic conditions and extracted RNA for bulk RNA sequencing. Each treatment group was set up with three replicates. Next, human tendon-derived fibroblasts cultured under normoxic conditions served as controls. Cells were treated with 0, 1, 2, or 5 mmol/L lactate under 5% O_2_ hypoxia, followed by protein and RNA extraction for subsequent experiments. In the second part, the human healthy tendon-derived fibroblasts were treated in 5% hypoxic environment containing lactate (5 mmol/L) with or without LOX (1 U/mL) and CAT (400 U/mL) for 24 h, followed by protein and RNA extraction for subsequent experiments. Each treatment group was set up with three replicates.

### NearestTemplatePrediction (NTP) analysis

To clarify the correspondence between animal models and cell models, and MPCs, NTP analysis was performed. First, the biomaRt package was used to convert homologous genes. The NTP module in GenePattern was used to assign each sample into either classifications.^64^

### Histological preparation and scoring

The isolated human or rat tendons were fixed in 4% paraformaldehyde for 24 h. Then, the samples were either paraffin-embedded or frozen-embedded according to the usual procedure. The histological score was performed based on H&E and Safranin O staining according to the scoring system that is modified from a previous study.^65,66^ For H&E staining, the eight parameters (fiber structure and arrangement, cell population, roundness of nuclei, inflammation, vascularity, and hyalinization) were semi-quantitatively assessed with a score range 0-21. For Safranin O staining, the GAG score was assessed. The lower score indicates better tissue quality.

### Compounds

PX-478 (HY-10231), sodium oxamate (HY-W013032A), temsirolimus (HY-50910), and lactate (HY-B2227) were purchased from MedChemExpress. LOX from Aerococcus viridans (L9795), CAT from bovine liver (SRE0041) and Hydrogen peroxide solution (323381) were purchased from Sigma. Recombinant Human IL-1b (CG93) and Recombinant Human TNF alpha (C008) were purchased from Novoprotein. Dexamethasone (S1322) was purchased from Selleck. The Betamethasone (J20140160) were purchased from Schering-Ploug Labo N.V.

### Antibodies

Anti-Collagen II antibody (ab34712), Anti-SOX9 antibody (ab185230) and Donkey Anti-Rabbit IgG H&L (Alexa Fluor® 555)(ab150074) were purchased from Abcam. HIF-1 alpha Polyclonal antibody (20960-1-AP), P4HA1 Polyclonal antibody (12658-1-AP), PLOD2-Specific Polyclonal antibody (21214-1-AP), Aggrecan Polyclonal antibody (13880-1-AP), LDHA-Specific Polyclonal antibody(19987-1-AP), GLUT1 Monoclonal antibody(66290-1-Ig) and Hexokinase 2 Monoclonal antibody (66974-1-Ig) were purchased from Proteintech. Human/Mouse TNF-alpha Antibody (AF-410) were purchased from R&D. Anti-α Smooth Muscle Actin (614851), HRP-labeled Goat Anti-Rabbit IgG(H + L) (A0208) and HRP-labeled Goat Anti-Mouse IgG(H + L) (A0216) were purchased from Beyotime.

### Oligonucleotides

Primers for Col2a1 gene: 5′-TGGACGATCAGGCGAAACC-3′; 5′-GCTGCGGATGCTCTCAATCT-3′. Primers for SOX9 gene: 5′-AGCGAACGCACATCAAGAC-3′; 5′-CTGTAGGCGATCTGTTGGGG-3′. Primers for β-actin gene: 5′-CATGTACGTTGCTATCCAGGC-3′; 5′-CTCCTTAATGTCACGCACGAT-3′. Primers for SLC2A1 gene: 5′-TCTGGCATCAACGCTGTCTTC-3′; 5′-CGATACCGGAGCCAATGGT-3′. Primers for HK2 gene: 5′-GAGCCACCACTCACCCTACT-3′; 5′-CCAGGCATTCGGCAATGTG-3′. Primers for LDHA gene: 5′-ATGGCAACTCTAAAGGATCAGC-3′; 5′-CCAACCCCAACAACTGTAAT

CT-3′.

### Quantification and statistical analysis

Statistical analysis was performed using SPSS program version 23.0 (IBM Corp., Armonk, NY). Normality was tested by the Shapiro-Wilk normality test. Levene’s test was performed for examination of equal variance. When variables presented normal distribution and equal variance, student’s *t* test was performed for comparison between two groups and one-way analysis of variance (ANOVA) was used for multiple group comparisons. Data failing normality or equal variance were analyzed by Mann-Whitney *U* test or Kruskal-Wallis test. Categorical or dichotomous variables were compared using the chi-squared test or Fisher’s exact test. Values of *P* < 0.05 were considered to be statistically significant. All data are presented as mean ± standard deviation (SD). Analyses of transcriptional data were conducted in R (version 4.2.0, The R Project for Statistical Computing, www.r-project.org).

## Supplementary information


Supplementary information
Fig. S1
Fig. S2
Fig. S3
Fig. S4
Fig. S5
Fig. S6
Fig. S7
Fig. S8
Fig. S9
Fig. S10
Fig. S11
Fig. S12
Fig. S13
Fig. S14
Fig. S15
Fig. S16
Fig. S17
Table S1
Table S2
Table S3
Table S4


## Data Availability

All data are available from the corresponding authors upon reasonable request. Single-cell RNA-seq data for human tendon tissue are available at the Genome Sequence Archive in National Genomics Data Center, China National Center for Bioinformation/Beijing Institute of Genomics, Chinese Academy of Sciences with the accession ID HRA001687; bulk RNA-seq data for human tendon tissue, animal model, and cell model are available at the same repository with the accession IDs HRA006534, HRA008332, CRA014406, and HRA008281, respectively; and LC-MS/MS proteomics data for human tendon tissue are available at the Metabolights repository with the accession ID MTBLS10869.
