# Supplementary material for: Metabolic-pathway-based classification of tendinopathy reveals a precise therapeutic strategy targeting chondro-modulatory tenocytes
Source: Bone Res. 2026 Aug 27;14:91. doi: 10.1038/s41413-026-00574-w (PMC13522621; doi:10.1038/s41413-026-00574-w)
Supplement: Supplementary file 1 [file 41413_2026_574_MOESM19_ESM.docx]

Table S1. Related to Figure 1. General clinical characteristics between disease and normal group.

|  | | Disease group | Normal group | p-value |
| --- | --- | --- | --- | --- |
|  |  | (n=192) | (n=29) |  |
| Age, year |  |  |  | < 0.001 |
|  | <50 | 13 (6.8%) | 28 (96.6%) |  |
|  | 50~60 | 56 (29.2%) | 1 (3.4%) |  |
|  | 60~70 | 91 (47.4%) | 0 (0%) |  |
|  | ≥70 | 32 (16.7%) | 0 (0%) |  |
| Sex, (%) |  |  |  | 0.642 |
|  | Male | 134 (69.8%) | 19 (65.5%) |  |
|  | Female | 58 (30.2%) | 10 (34.5%) |  |
| BMI, kg/m^2^ |  |  |  | 0.365 |
|  | <18.5 | 8 (4.2%) | 0 (0%) |  |
|  | 18.5~25 | 127 (66.1%) | 19 (65.5%) |  |
|  | 25~30 | 49 (25.5%) | 10 (34.5%) |  |
|  | ≥30 | 8 (4.2%) | 0 (0%) |  |
| Hypertension, (%) |  |  |  | 0.010 |
|  | Yes | 57 (29.7%) | 2 (6.9%) |  |
|  | No | 135 (70.3%) | 27 (93.1%) |  |
| Diabetes mellitus, (%) |  |  |  | 0.701 |
|  | Yes | 15 (7.8%) | 1 (3.4%) |  |
|  | No | 177 (92.2%) | 28 (96.6%) |  |
