# Supplementary material for: Metabolic-pathway-based classification of tendinopathy reveals a precise therapeutic strategy targeting chondro-modulatory tenocytes
Source: Bone Res. 2026 Aug 27;14:91. doi: 10.1038/s41413-026-00574-w (PMC13522621; doi:10.1038/s41413-026-00574-w)
Supplement: Supplementary file 2 [file 41413_2026_574_MOESM1_ESM.docx]

**Supplementary Information**

Metabolic-pathway-based classification of tendinopathy reveals a precise therapeutic strategy targeting chondro-modulatory tenocytes

Junchao Luo^1,2,3,4,5,7#^, Zetao Wang^1,2,3,4,5,7#^, Ruifu Lin^2,3,4,5,7#^, Jiayun Huang^1,2,3,4,5,7^, Chenqi Tang^2,3,4,5,6,7^, Honglu Cai^1,2,3,4,5^, Yang Fei^1,2,3,4,5,7,8^, Dengfeng Ruan^1,2,3,4,5^, Xinji Wang^1,2,3,4,5^, Peiru Li^7^, Menyun Liu^2,3,4,5,7^, Ruojin Yan^7,8^, Chunmei Fan^7,8^, Canlong Wang^1,2,3,4,5^, Cunqi Ye^9^, Hongwei Ouyang^7,8,10^, Zi Yin^7,8*^, Xiao Chen^7,8*^, Weiliang Shen^1,2,3,4,5,7,8*^

This supporting information includes:

Key resources table

Supplementary Figures S1 to S17

Supplementary Table S1 to S4

**Key resources table**

| REAGENT or RESOURCE | SOURCE | IDENTIFIER |
| --- | --- | --- |
| Antibodies |  |  |
| Anti-Collagen II antibody | Abcam | ab34712 |
| Anti-SOX9 antibody | Abcam | ab185230 |
| Donkey Anti-Rabbit IgG H&L (Alexa Fluor® 555) | Abcam | ab150074 |
| HIF-1 alpha Polyclonal antibody | Proteintech | 20960-1-AP |
| P4HA1 Polyclonal antibody | Proteintech | 12658-1-AP |
| PLOD2-Specific Polyclonal antibody | Proteintech | 21214-1-AP |
| Aggrecan Polyclonal antibody | Proteintech | 13880-1-AP |
| LDHA-Specific Polyclonal antibody | Proteintech | 19987-1-AP |
| GLUT1 Monoclonal antibody | Proteintech | 66290-1-Ig |
| Hexokinase 2 Monoclonal antibody | Proteintech | 66974-1-Ig |
| Human/Mouse TNF-alpha Antibody | R&D | AF-410 |
| Anti-α Smooth Muscle Actin Antibody | Biolgend | 614851 |
| HRP-labeled Goat Anti-Rabbit IgG(H+L) | Beyotime | A0208 |
| HRP-labeled Goat Anti-Mouse IgG(H+L) | Beyotime | A0216 |
| Biological Samples |  |  |
| Human rotator cuff tendon tissue sample | This study | N/A |
| Human hamstring tendon tissue sample | This study | N/A |
| Chemicals, Peptides, and Recombinant Proteins |  |  |
| PX-478 | MedChemExpress | HY-10231 |
| Sodium oxamate | MedChemExpress | HY-W013032A |
| Temsirolimus | MedChemExpress | HY-50910 |
| Lactate | MedChemExpress | HY-B2227 |
| LOX from Aerococcus viridans | Sigma | L9795 |
| CAT from bovine liver | Sigma | SRE0041 |
| Hydrogen peroxide solution | Sigma | 323381 |
| Recombinant Human IL-1b | Novoprotein | CG93 |
| Recombinant Human TNF alpha | Novoprotein | C008 |
| Dexamethasone | Selleck | S1322 |
| Betamethasone | Schering-Ploug Labo N.V. | J20140160 |
| Critical Commercial Assays |  |  |
| Cell Counting Kit-8 | Beyotime | C0037 |
| NAD/NADH Ratio Fluorimetric Detection Assay Kit (Red Fluorescence) | Yeasen | 50130ES72 |
| Deposited Data |  |  |
| BulkRNA-seq for human tendon tissue | Genome Sequence Archive in National Genomics Data Center, China National Center for Bioinformation / Beijing Institute of Genomics, Chinese Academy of Sciences | HRA006534;HRA008332 |
| BulkRNA-seq for animal model | Genome Sequence Archive in National Genomics Data Center, China National Center for Bioinformation / Beijing Institute of Genomics, Chinese Academy of Sciences | CRA014406 |
| BulkRNA-seq for cell model | Genome Sequence Archive in National Genomics Data Center, China National Center for Bioinformation / Beijing Institute of Genomics, Chinese Academy of Sciences | HRA008281 |
| BulkRNA-seq for human tendon derived fibroblast treated with lactate | Genome Sequence Archive in National Genomics Data Center, China National Center for Bioinformation / Beijing Institute of Genomics, Chinese Academy of Sciences | HRA016859 |
| scRNA-seq for human tendon tissue | Genome Sequence Archive in National Genomics Data Center, China National Center for Bioinformation / Beijing Institute of Genomics, Chinese Academy of Sciences | HRA001687 |
| LC-MS/MS anslysis for human tendon tissue | Genome Sequence Archive in National Genomics Data Center, China National Center for Bioinformation / Beijing Institute of Genomics, Chinese Academy of Sciences | MTBLS10869 |
| Oligonucleotides |  |  |
| Primers for Col2a1 gene; 5'-TGGACGATCAGGCGAAACC-3' 5'-GCTGCGGATGCTCTCAATCT-3' | N/A | N/A |
| Primers for SOX9 gene; 5'-AGCGAACGCACATCAAGAC-3' 5'-CTGTAGGCGATCTGTTGGGG-3' | N/A | N/A |
| Primers for β-actin gene; 5'-CATGTACGTTGCTATCCAGGC-3' 5'-CTCCTTAATGTCACGCACGAT-3' | N/A | N/A |
| Primers for SLC2A1 gene; 5'-TCTGGCATCAACGCTGTCTTC-3' 5'-CGATACCGGAGCCAATGGT-3' | N/A | N/A |
| Primers for HK2 gene; 5'-GAGCCACCACTCACCCTACT-3' 5'-CCAGGCATTCGGCAATGTG-3' | N/A | N/A |
| Primers for LDHA gene; 5'-ATGGCAACTCTAAAGGATCAGC-3' 5'-CCAACCCCAACAACTGTAATCT-3' | N/A | N/A |
| Software and Algorithms |  |  |
| Fiji / ImageJ software | NIH | https://imagej.net/Fiji |
| R version 4.2.1 | R Foundation for Statistical Computing | https://cran.r-project.org |
| GraphPad Prism software version 9.0 | GraphPad Software | https://www.graphpad.com |
| MATLAB version R2022b | MathWorks | <https://www.mathworks.com/products/matlab.html> |
| SPSS program version 23.0 | N/A | https://www.ibm.com/spss |
| Gene Set Enrichment Analysis (GSEA) version 4.3.2 | ^66^ | http://software.broadinstitute.org/cancer/software/genepattern |
| Gene set variation analysis (GSVA) | ^16^ | http://www.bioconductor.org/packages/release/bioc/html/GSVA.html |
| DESeq2 | ^59^ | https://bioconductor.org/packages/release/bioc/html/DESeq2.html |
| NMF | N/A | https://cran.r-project.org/ |
| pheatmap | N/A | <https://www.rdocumentation.org/packages/pheatmap/versions/1.0.2> |

**Supplementary Figure S1.

Supplementary Fig. S1 Related to Figure 1. Detailed description of human tendon single-cell atlas. a-b** Feature plots and violin plots of depicting single-cell gene expression of representative gene markers. **c** Pie plots showing cell-type composition in diseased (Up) and normal (Down) tissues. Cell types are colored as in b. **d** GSVA of carbohydrate and lipid metabolism in normal versus diseased cells. Cell types are colored as in b.

**Supplementary Figure S2.**

**Supplementary Fig. S2 Related to Figure 1. Differences in the expression of tissue-specific genes between diseased and normal tendons. a-e** mRNA expression differences of tendon-specific, adipose-specific, muscle-specific, vascular-specific, and cartilage-specific genes between diseased and normal tendons.

**Supplementary Figure S3.**

**Supplementary Fig. S3 Related to Figure 2. Process and validation of subseting based on metabolic genes and pathways.**

**a** Cophenetic correlation coefficient plots for metabolic-gene-based classifications (MGCs). The NMF input was the normalized RNA-seq matrix of the 2732 metabolic genes, across 192 diseased samples. Based on visual inspection of the hierarchical subseting and the profiles of cophenetic correlation coefficient with 2 to 7 subsets, we consider K = 3 to be the preferred solution (indicated by black triangles) and use this to arrange the samples shown in C and D. **b** Heatmap of NMF consensus matrix for MGCs. **c** PCA of tendinopathy samples (*n = 192*) for the mRNA expression of metabolic genes. **d** Heatmap shows normalized metabolic gene expressions of 3 MGCs (top 25% genes by standard error). **e** Cophenetic correlation coefficient plots for MPCs. **f** Heatmap of NMF consensus matrix for MPCs. The NMF input was the activity matrix of the 148 metabolic pathways, across 192 diseased samples. Based on visual inspection of the hierarchical subseting and the profiles of cophenetic correlation coefficient with 2 to 7 subsets, we consider K = 3 to be the preferred solution (indicated by black triangles).

**Supplementary Figure S4.**

**Supplementary Fig. S4 Related to Figure 2. Transcriptomic characterization of metabolic-pathway-based classifications. a** PCA of metabolic pathway enrichment scores in 192 tendinopathy samples. **b** Representative GSEA plots of differentially enriched pathways among three MPCs and normal tissues. **c** KEGG enrichment analysis for simultaneously differentially expressed metabolic genes between diseased tendon or its classifications and normal tendon.

**Supplementary Figure S5.**

**Supplementary Fig. S5 Related to Figure 2. Metabolomic, clinical functional, and histological profiles of metabolic-pathway-based classifications. a** PCA of metabolite abundance in 17 diseased tendons. **b** Volcano plots showing differences in metabolite abundance between MPC pairs. **c** Heatmap of metabolite abundance for three MPCs. **d-f** Relative intensity levels of metabolites involved in fatty acid oxidation, vitamin/cofactor metabolism, and amino acid metabolism across three MPCs. **g** Frequency distribution of tendon stump and joint synovium color among three MPCs. **h** Preoperative range of motion of shoulder joint. **i** Histological total score, cell density, fibre structure, fibre arrangement, round of nuclei and hyalinization among three MPCs.

**Supplementary Figure S6.**

**Supplementary Fig. S6 Differences in the expression of tissue-specific genes between diseased and normal tendons. a-e** mRNA expression differences of tendon-specific, adipose-specific, muscle-specific, vascular-specific, and cartilage-specific genes between diseased and normal tendons.

**Supplementary Figure S7.**

**Supplementary Fig. S7 Related to Figure 2. Immunohistochemistry showing protein expresssion of glucose utilization related transporters and enzymes between three metabolic-pathway-based classfications. a** Representative immunohistochemical images of proteins associated with glucose utilization among three MPCs. Scale bar, 50 μm. **b** Semi-quantitative analysis of immunohistochemical images. (*n = 3*) The data were presented as the mean ± SD.

**Supplementary Figure S8.**

**Supplementary Fig. S8 Related to Figure 2. Liver function, blood coagulation function and glucose profile among different metabolic-pathway-based classifications of tendipathy. a** Preoperative liver function. **b** Preoperative blood coagulation function. **c** Preoperative glucose profile.

**Supplementary Figure S9.**

**Supplementary Fig. S9 Related to Figure 3. Changes in metabolic and functional states in different animal models. a** PCA of mRNA expression in different animal models. **b** Volcano plot of genes related to cartilage-like matrix accumulation between hypoxic-dominant and inflammatory-dominant models. **c-d** Representative H&E and S. O. staining. Scale bar, 100 μm. **e** Differentially expressed genes of *HIF-1α* signaling pathway between hypoxic and inflammatory models. **f** Differentially expressed genes of glycolysis, oxidative phosphorylation, and fatty acid oxidation between hypoxic-dominant and inflammatory-dominant models.

**Supplementary Figure S10.**

**Supplementary Fig. S10 Related to Figure 3. Changes in metabolic states in different cell models. a** PCA of mRNA expression in different cell models. **b** CCK-8 assay showing the effects of IL-1β, H_2_O_2_ and TNF-α on the viability of tendon fibroblasts. **c-d** CCK-8 assay and light microscopy showing the effect of 2.5% hypoxia on the viability of tendon fibroblasts. Scale bar, 400 μm. **e** KEGG enrichment analysis of differentially expressed genes in each cell model versus normal. **f-g** Differentially expressed genes of *HIF-1α* signaling pathway between hypoxia group and H_2_O_2_ groups versus normal. **h** Differentially expressed genes of fatty acid oxidation in hypoxia versus normal group. **i** GSVA of carbohydrate metabolism in different cell models.

**Supplementary Figure S11.**

**Supplementary Fig. S11 Related to Figure 3. Changes in functional states in different cell models. a-b** Volcano plots of genes related to cartilage-like matrix accumulation in hypoxia and H_2_O_2_ groups versus normal. **c** Immunofluorescence of *HIF-1α*, *PLOD2* and *P4HA1* under hypoxia. Scale bar, 50 μm. **d-f** GSVA of angiogenesis, chondrification, and ossification pathways in different cell models.

**Supplementary Figure S12.**

**Supplementary Fig. S12 Related to Figure 4. Molecular characterization of human tendon stromal cell subsets. a** Violin plots of representative gene markers. **b** Heatmap of representative gene markers. **c** Average expression levels of representative metabolic genes for each subset. **d** Feature plots of depicting single-cell gene expression of representative gene markers.

**Supplementary Figure S13.**

**Supplementary Fig. S13 Related to Figure 4. Cellular composition, classification association, and functional features of stromal subsets. a** Bar plots showing the cell-subset composition within the disease tissues and normal tissues. **b** Heatmap of predicted MPC probabilities for each scRNA-seq donor (random forest classifier) and violin plots of MPC-G signature scores in chondro-modulatory tenocytes (subsets 5, 6, and 7) across donors. **c** Bar plot showing the percentage distribution of stromal cell subsets across donors with different predicted MPCs. **d** The bubble plot displays expression differences of tendon-related, fibrosis, chondrogenic and osteogenic genes across distinct subsets. **e-f** Immunohistochemistry of *Col2α1*, *SOX9*, *P4HA1* and *PLOD2* and semi-quantitative analysis. Scale bar, 50 μm. (*n = 3*) **g** CytoTRACE analysis showing that subsets 5 and 7 exhibited relatively high proliferative potential. **h** CytoTRACE was used to evaluate the proliferative potential of tenocytes..

**Supplementary Figure S14.**

**Supplementary Fig. S14 Related to Figure 4. HIF-1α drives the formation of MPC-G pathogenic cells. a** CCK-8 assay of PX-478 on tendon fibroblast viability. **b** Effect of PX-478 on *SOX9* and *Col2α1* mRNA under 2.5% hypoxia. (*n=3*) **c** Immunofluorescence showing the effect of PX-478 on the protein expression of *PLOD2* and *P4HA1* under 2.5% hypoxia. Scale bar, 50 μm. **d-e** Pellet cultures of tendon fibroblasts under 2.5% hypoxia with chondrogenic medium, with or without PX-478. Scale bar, 400μm. (*n = 3*) **f** Western blot analysis revealed the difference in *HIF-1α* levels between HIF-1α^fibro+^ and HIF-1α^fibro-^ mice. **g** Round of nuclei, number of vessels, cell counts and hyalinization among HIF-1α^fibro+^ and HIF-1α^fibro-^ mice. (*n = 5*)

**Supplementary Figure S15.**

**Supplementary Fig. S15 Related to Figure 5. Glycolytic inhibitors inhibit the formation of MPC-G pathogenic cells. a** Gene set variation analysis reveals that chondro-modulatory tenocytes (subsets 5, 6, and 7) exhibit similar carbohydrate metabolic patterns. **b** Effect of PX-478 on the *HIF-1α*, *SLC2A1*, *HK2* and *LDHA* mRNA expression at 2.5% hypoxia. (*n = 3*). **c** Differentially expressed genes of extracelluar matrix organization pathway between lactate treatment and control group. **d** CCK-8 assay showing the effects of sodium oxamate on the viability of tendon fibroblasts. **e** Effect of sodium oxamate on the *SOX9* and *Col2α1* mRNA expression at 2.5% hypoxia. (*n = 3*). **f** Immunofluorescence showing the effect of sodium oxamate on the protein expression of *Col2α1* under 2.5% hypoxia. Scale bar, 50 μm. **g-h** Western blot and semi-quantification of *SOX9* under 2.5% hypoxia with or without oxamate. **i** Representative images of Achilles tendon in different groups of rats. **j** Effect of temsirolimus on Achilles tendon width in MPC-G animal model induced by type 1 collagenase combined with transverse incision. (*n = 5*) **k** Effect of temsirolimus on round of nuclei, number of vessels, fibre arrangement and cell counts in MPC-G models induced by type 1 collagenase combined with transverse incision. (*n = 5*) **l** Semi-quantitative analysis of western blot results demonstrating LOX/CAT inhibiting *HIF-1α* accumulation under 5% hypoxia **m** Effect of LOX/CAT on *SOX9*, *PLOD2* and *P4HA1* mRNA expression under 5% hypoxia. (*n = 3*). **n** LOX/CAT inhibiting *SOX9* protein expression under 5% hypoxia. Scale bar, 50 μm. (*n = 3*)

**Supplementary Figure S16.**

**Supplementary Fig. S16 Related to Figure 6. The effects of mTOR inhibitors on MPC-G cell and animal model. a** CCK-8 assay of temsirolimus on tendon fibroblast viability. **b** Effects of temsirolimus on the *HIF-1α*, *SOX9, SLC2A1*, *HK2* and *LDHA* mRNA expression at 2.5% hypoxia. **c** Representative images of Achilles tendon in different groups of rats. **d** Effect of temsirolimus on Achilles tendon width in MPC-G animal model. (*n = 5*) **e** Representative H&E and SO staining. Scale bar, 100 μm.

**f** Effect of temsirolimus on histological total score and cell counts in MPC-G animal model. (*n = 5*) **g** Effect of temsirolimus on GAG content score in MPC-G animal model. (*n = 5*) **h** Effect of temsirolimus on semi-quantitative scoring of GAG content in MPC-G animal model. (*n = 5*) **i** Effect of temsirolimus on round of nuclei, number of vessels, cell counts and hyalinization MPC-G models induced by type 1 collagenase combined with transection. (*n = 5*)

**Supplementary Figure S17.**

**Supplementary Fig. S17 Related to Figure 6. Related to Figure 6. The effects of glucocorticoid agonists on MPC-I animal model. a** Using Cmap analysis to predict potential therapeutic targets for reversing MPC-I metabolic dysregulation, based on the top 200 overlapping differentially expressed metabolic genes with higher expression in MPC-I compared to other classifications or normal tendons. **b** GSVA of glucocorticoid effect on fatty acid oxidation in MPC-I animal model. **c** Representative H&E staining of glucocorticoid in the treatment of MPC-I animal model. Scale bar, 100 μm. **d** Effect of glucocorticoid on histological total score, fibre structure, fibre arrangement, vascular density, cell density, and round of nuclei. (*n = 5*) **e** Differentially expressed genes of vascular endothelial growth factor signaling pathway and matrix metal degrading enzymes after glucocorticoid treatment in MPC-I animal model.

Table S1. Related to Figure 1. General clinical characteristics between disease and normal group.

Table S2. Related to Figure 1. KEGG human metabolic pathway gene list.

Table S3. Related to Figure 1. Reactome human metabolic pathway gene list.

Table S4. Related to Figure 2. General clinical characteristics among different metabolic-pathway-based classifications of tendinopathy.
