# Supplementary material for: Metabolic-pathway-based classification of tendinopathy reveals a precise therapeutic strategy targeting chondro-modulatory tenocytes
Source: Bone Res. 2026 Aug 27;14:91. doi: 10.1038/s41413-026-00574-w (PMC13522621; doi:10.1038/s41413-026-00574-w)
Supplement: Supplementary file 3 [file 41413_2026_574_MOESM20_ESM.docx]

Table S2. Related to Figure 1. KEGG human metabolic pathway gene list.

| Gene symbol | Metabolic pathways involved |
| --- | --- |
| A3GALT2 | Glycosphingolipid biosynthesis-globo and isoglobo series |
| A4GALT | Glycosphingolipid biosynthesis-globo and isoglobo series; Glycosphingolipid biosynthesis-lacto and neolacto series |
| AACS | Butanoate metabolism; Valine, leucine and isoleucine degradation |
| AADAT | Lysine degradation; Tryptophan metabolism |
| AANAT | Tryptophan metabolism |
| AASDH | Lysine degradation |
| AASDHPPT | Pantothenate and CoA biosynthesis |
| AASS | Lysine degradation |
| ABAT | Alanine, aspartate and glutamate metabolism; beta-Alanine metabolism; Butanoate metabolism; Propanoate metabolism; Valine, leucine and isoleucine degradation |
| ABO | Glycosphingolipid biosynthesis-lacto and neolacto series |
| ABP1 | Arginine and proline metabolism; Histidine metabolism, Tryptophan metabolism |
| ACAA1 | alpha-Linolenic acid metabolism; Biosynthesis of unsaturated fatty acids; Fatty acid degradation; Valine, leucine and isoleucine degradation |
| ACAA2 | Fatty acid degradation; Fatty acid elongation; Valine, leucine and isoleucine degradation |
| ACACA | Fatty acid biosynthesis; Propanoate metabolism; Pyruvate metabolism |
| ACACB | Fatty acid biosynthesis; Propanoate metabolism; Pyruvate metabolism |
| ACAD8 | Valine, leucine and isoleucine degradation |
| ACADL | Fatty acid degradation |
| ACADM | beta-Alanine metabolism; Fatty acid degradation; Propanoate metabolism; Valine, leucine and isoleucine degradation |
| ACADS | Butanoate metabolism; Fatty acid degradation; Valine, leucine and isoleucine degradation |
| ACADSB | Fatty acid degradation; Valine, leucine and isoleucine degradation |
| ACADVL | Fatty acid degradation |
| ACAT1 | Butanoate metabolism; Fatty acid degradation; Glyoxylate and dicarboxylate metabolism; Lysine degradation; Propanoate metabolism; Pyruvate metabolism; Terpenoid backbone biosynthesis; Tryptophan metabolism; Valine, leucine and isoleucine degradation |
| ACAT2 | Butanoate metabolism; Fatty acid degradation; Glyoxylate and dicarboxylate metabolism; Lysine degradation; Propanoate metabolism; Pyruvate metabolism; Terpenoid backbone biosynthesis; Tryptophan metabolism; Valine, leucine and isoleucine degradation |
| ACER1 | Sphingolipid metabolism |
| ACER2 | Sphingolipid metabolism |
| ACER3 | Sphingolipid metabolism |
| ACHE | Glycerophospholipid metabolism |
| ACLY | TCA cycle |
| ACMSD | Tryptophan metabolism |
| ACO1 | Glyoxylate and dicarboxylate metabolism; TCA cycle |
| ACO2 | Glyoxylate and dicarboxylate metabolism; TCA cycle |
| ACOT1 | Biosynthesis of unsaturated fatty acids; Fatty acid elongation |
| ACOT12 | Pyruvate metabolism |
| ACOT2 | Biosynthesis of unsaturated fatty acids; Fatty acid elongation |
| ACOT4 | Biosynthesis of unsaturated fatty acids; Fatty acid elongation |
| ACOT7 | Biosynthesis of unsaturated fatty acids; Fatty acid elongation |
| ACOX1 | alpha-Linolenic acid metabolism; Biosynthesis of unsaturated fatty acids; Fatty acid degradation |
| ACOX3 | alpha-Linolenic acid metabolism; Biosynthesis of unsaturated fatty acids; Fatty acid degradation |
| ACP1 | Riboflavin metabolism; Thiamine metabolism |
| ACP2 | Riboflavin metabolism |
| ACP5 | Riboflavin metabolism |
| ACSBG1 | Fatty acid biosynthesis; Fatty acid degradation |
| ACSBG2 | Fatty acid biosynthesis; Fatty acid degradation |
| ACSF3 | Valine, leucine and isoleucine degradation |
| ACSL1 | Fatty acid biosynthesis; Fatty acid degradation |
| ACSL3 | Fatty acid biosynthesis; Fatty acid degradation |
| ACSL4 | Fatty acid biosynthesis; Fatty acid degradation |
| ACSL5 | Fatty acid biosynthesis; Fatty acid degradation |
| ACSL6 | Fatty acid biosynthesis; Fatty acid degradation |
| ACSM1 | Butanoate metabolism |
| ACSM2 | Butanoate metabolism |
| ACSM2A | Butanoate metabolism |
| ACSM3 | Butanoate metabolism |
| ACSM4 | Butanoate metabolism |
| ACSM5 | Butanoate metabolism |
| ACSS1 | Glycolysis; Gluconeogenesis; Glyoxylate and dicarboxylate metabolism; Propanoate metabolism; Pyruvate metabolism |
| ACSS2 | Glycolysis; Gluconeogenesis; Glyoxylate and dicarboxylate metabolism; Propanoate metabolism; Pyruvate metabolism |
| ACSS3 | Propanoate metabolism |
| ACY1 | Arginine biosynthesis |
| ACY3 | Alanine aspartate and glutamate metabolism；Histidine metabolism |
| ACYP1 | Pyruvate metabolism |
| ACYP2 | Pyruvate metabolism |
| ADA | Purine metabolism |
| ADC | Arginine and proline metabolism |
| ADCY1 | Purine metabolism |
| ADCY10 | Purine metabolism |
| ADCY2 | Purine metabolism |
| ADCY3 | Purine metabolism |
| ADCY4 | Purine metabolism |
| ADCY5 | Purine metabolism |
| ADCY6 | Purine metabolism |
| ADCY7 | Purine metabolism |
| ADCY8 | Purine metabolism |
| ADCY9 | Purine metabolism |
| ADH1A | Drug metabolism-cytochrome P450; Fatty acid degradation; Gluconeogenesis; Glycolysis; Metabolism of xenobiotics by cytochrome P450; Retinol metabolism; Tyrosine metabolism |
| ADH1B | Drug metabolism-cytochrome P450; Fatty acid degradation; Gluconeogenesis; Glycolysis; Metabolism of xenobiotics by cytochrome P450; Retinol metabolism; Tyrosine metabolism |
| ADH1C | Drug metabolism-cytochrome P450; Fatty acid degradation; Gluconeogenesis; Glycolysis; Metabolism of xenobiotics by cytochrome P450; Retinol metabolism; Tyrosine metabolism |
| ADH4 | Drug metabolism-cytochrome P450; Fatty acid degradation; Gluconeogenesis; Glycolysis; Metabolism of xenobiotics by cytochrome P450; Retinol metabolism; Tyrosine metabolism |
| ADH5 | Drug metabolism-cytochrome P450; Fatty acid degradation; Gluconeogenesis; Glycolysis; Metabolism of xenobiotics by cytochrome P450; Retinol metabolism; Tyrosine metabolism |
| ADH6 | Drug metabolism-cytochrome P450; Fatty acid degradation; Gluconeogenesis; Glycolysis; Metabolism of xenobiotics by cytochrome P450; Retinol metabolism; Tyrosine metabolism |
| ADH7 | Drug metabolism-cytochrome P450; Fatty acid degradation; Gluconeogenesis; Glycolysis; Metabolism of xenobiotics by cytochrome P450; Retinol metabolism; Tyrosine metabolism |
| ADI1 | Cysteine and methionine metabolism |
| ADK | Purine metabolism |
| ADO | Taurine and hypotaurine metabolism |
| ADPGK | Glycolysis; Gluconeogenesis |
| ADPRM | Glycerophospholipid metabolism；Purine metabolism |
| ADSL | Alanine aspartate and glutamate metabolism；Purine metabolism |
| ADSS | Alanine aspartate and glutamate metabolism；Purine metabolism |
| ADSS1 | Alanine aspartate and glutamate metabolism；Purine metabolism |
| ADSS2 | Alanine aspartate and glutamate metabolism；Purine metabolism |
| ADSSL1 | Arginine and proline metabolism；Histidine metabolism；Tryptophan metabolism |
| AFMID | Oxidative phosphorylation |
| AGA | Oxidative phosphorylation |
| AGK | Oxidative phosphorylation |
| AGL | Oxidative phosphorylation |
| AGMAT | Oxidative phosphorylation |
| AGPAT1 | Oxidative phosphorylation |
| AGPAT2 | oxidative phosphorylation |
| AGPAT3 | oxidative phosphorylation |
| AGPAT4 | oxidative phosphorylation |
| AGPAT5 | oxidative phosphorylation |
| AGPAT6 | oxidative phosphorylation |
| AGPAT9 | oxidative phosphorylation |
| AGPHD1 | oxidative phosphorylation |
| AGPS | oxidative phosphorylation |
| AGXT | oxidative phosphorylation |
| AGXT2 | oxidative phosphorylation |
| AGXT2L1 | Glycerophospholipid metabolism |
| AGXT2L2 | Lysine degradation |
| AHCY | Cysteine and methionine metabolism |
| AHCYL1 | Cysteine and methionine metabolism |
| AHCYL2 | Cysteine and methionine metabolism |
| AK1 | Purine metabolism; Thiamine metabolism |
| AK2 | Purine metabolism; Thiamine metabolism |
| AK3 | Purine metabolism; Purine metabolism; Thiamine metabolism |
| AK4 | Purine metabolism |
| AK5 | Folate biosynthesis |
| AK6 | Arginine and proline metabolism；Histidine metabolism；Tryptophan metabolism |
| AK7 | Purine metabolism; Thiamine metabolism |
| AKD1 | Purine metabolism; Pyrimidine metabolism |
| AKR1A1 | Glycerolipid metabolism; Glycerolipid metabolism; Glycolysis; Gluconeogenesis; Pentose and glucuronate interconversions |
| AKR1B1 | Folate biosynthesis; Fructose and mannose metabolism; Galactose metabolism; Glycerolipid metabolism; Glycerolipid metabolism; Pentose and glucuronate interconversions |
| AKR1B10 | Folate biosynthesis; Fructose and mannose metabolism; Galactose metabolism; Glycerolipid metabolism; Glycerolipid metabolism; Pentose and glucuronate interconversions |
| AKR1C1 | Metabolism of xenobiotics by cytochrome P450 |
| AKR1C3 | Arachidonic acid metabolism; Folate biosynthesis |
| AKR7A2 | Metabolism of xenobiotics by cytochrome P450 |
| AKR7A3 | Metabolism of xenobiotics by cytochrome P450 |
| ALAD | Porphyrin and chlorophyll metabolism |
| ALAS1 | Glycine, serine and threonine metabolism; Porphyrin and chlorophyll metabolism |
| ALAS2 | Glycine, serine and threonine metabolism; Porphyrin and chlorophyll metabolism |
| ALDH18A1 | Arginine and proline metabolism |
| ALDH1A1 | Retinol metabolism |
| ALDH1A2 | Retinol metabolism |
| ALDH1A3 | beta-Alanine metabolism; Drug metabolism-cytochrome P450; Gluconeogenesis; Glycolysis; Histidine metabolism; Metabolism of xenobiotics by cytochrome P450; Phenylalanine metabolism; Tyrosine metabolism |
| ALDH1B1 | Arginine and proline metabolism; Ascorbate and aldarate metabolism; beta-Alanine metabolism; Fatty acid degradation; Glycerolipid metabolism; Glycerolipid metabolism; Glycolysis; Gluconeogenesis; Histidine metabolism; Lysine degradation; Pyruvate metabolism; Tryptophan metabolism; Valine, leucine and isoleucine degradation |
| ALDH1L1 | One carbon pool by folate |
| ALDH1L2 | One carbon pool by folate |
| ALDH2 | Arginine and proline metabolism; Ascorbate and aldarate metabolism; beta-Alanine metabolism; Fatty acid degradation; Gluconeogenesis; Glycerolipid metabolism; Glycerolipid metabolism; Glycolysis; Gluconeogenesis; Histidine metabolism; Lysine degradation; Pyruvate metabolism; Tryptophan metabolism; Valine, leucine and isoleucine degradation |
| ALDH3A1 | beta-Alanine metabolism; Drug metabolism-cytochrome P450; Glycolysis; Gluconeogenesis; Histidine metabolism; Metabolism of xenobiotics by cytochrome P450; Phenylalanine metabolism; Tyrosine metabolism |
| ALDH3A2 | Arginine and proline metabolism; Ascorbate and aldarate metabolism; beta-Alanine metabolism; Fatty acid degradation; Gluconeogenesis; Glycerolipid metabolism; Glycerolipid metabolism; Glycolysis; Gluconeogenesis; Histidine metabolism; Lysine degradation; Pyruvate metabolism; Tryptophan metabolism; Valine, leucine and isoleucine degradation |
| ALDH3B1 | beta-Alanine metabolism; Drug metabolism-cytochrome P450; Glycolysis; Gluconeogenesis; Histidine metabolism; Metabolism of xenobiotics by cytochrome P450; Phenylalanine metabolism; Tyrosine metabolism |
| ALDH3B2 | beta-Alanine metabolism; Drug metabolism-cytochrome P450; Glycolysis; Gluconeogenesis; Histidine metabolism; Metabolism of xenobiotics by cytochrome P450; Phenylalanine metabolism; Tyrosine metabolism |
| ALDH4A1 | Alanine, aspartate and glutamate metabolism; Arginine and proline metabolism |
| ALDH5A1 | Alanine, aspartate and glutamate metabolism; Butanoate metabolism |
| ALDH6A1 | beta-Alanine metabolism; Inositol phosphate metabolism; Propanoate metabolism; Valine, leucine and isoleucine degradation |
| ALDH7A1 | Arginine and proline metabolism; Ascorbate and aldarate metabolism; beta-Alanine metabolism; Fatty acid degradation; Glycerolipid metabolism; Glycerolipid metabolism; Glycine, serine and threonine metabolism; Glycolysis; Gluconeogenesis; Histidine metabolism; Lysine degradation; Pyruvate metabolism; Tryptophan metabolism; Valine, leucine and isoleucine degradation |
| ALDH9A1 | Arginine and proline metabolism; Ascorbate and aldarate metabolism; beta-Alanine metabolism; Fatty acid degradation; Glycerolipid metabolism; Glycerolipid metabolism; Glycolysis; Gluconeogenesis; Histidine metabolism; Lysine degradation; Pyruvate metabolism; Tryptophan metabolism; Valine, leucine and isoleucine degradation |
| ALDOA | Fructose and mannose metabolism; Glycolysis; Gluconeogenesis; Pentose phosphate pathway |
| ALDOB | Fructose and mannose metabolism; Glycolysis; Gluconeogenesis; Pentose phosphate pathway |
| ALDOC | Fructose and mannose metabolism; Glycolysis; Gluconeogenesis; Pentose phosphate pathway |
| ALG1 | Various types of N-glycan biosynthesis |
| ALG10 | N-Glycan biosynthesis |
| ALG10B | N-Glycan biosynthesis |
| ALG11 | Various types of N-glycan biosynthesis |
| ALG12 | Various types of N-glycan biosynthesis |
| ALG13 | Various types of N-glycan biosynthesis |
| ALG14 | Various types of N-glycan biosynthesis |
| ALG2 | Various types of N-glycan biosynthesis |
| ALG3 | Various types of N-glycan biosynthesis |
| ALG5 | N-Glycan biosynthesis |
| ALG6 | N-Glycan biosynthesis |
| ALG8 | N-Glycan biosynthesis |
| ALG9 | Various types of N-glycan biosynthesis |
| ALLC | Purine metabolism |
| ALOX12 | Arachidonic acid metabolism |
| ALOX12B | Arachidonic acid metabolism |
| ALOX15 | Arachidonic acid metabolism; Linoleic acid metabolism |
| ALOX15B | Arachidonic acid metabolism |
| ALOX5 | Arachidonic acid metabolism |
| ALPG | Folate biosynthesis |
| ALPI | Arginine and proline metabolism；Histidine metabolism；Tryptophan metabolism |
| ALPL | Folate biosynthesis; Thiamine metabolism |
| ALPP | Folate biosynthesis; Thiamine metabolism |
| ALPPL2 | Folate biosynthesis; Thiamine metabolism |
| AMD1 | Arginine and proline metabolism; Cysteine and methionine metabolism |
| AMDHD1 | Histidine metabolism |
| AMDHD2 | Amino sugar and nucleotide sugar metabolism |
| AMPD1 | Purine metabolism |
| AMPD2 | Purine metabolism |
| AMPD3 | Purine metabolism |
| AMT | Glycine, serine and threonine metabolism; Glyoxylate and dicarboxylate metabolism; One carbon pool by folate |
| AMY1A | Starch and sucrose metabolism |
| AMY1B | Starch and sucrose metabolism |
| AMY1C | Starch and sucrose metabolism |
| AMY2A | Starch and sucrose metabolism |
| AMY2B | Starch and sucrose metabolism |
| ANPEP | Glutathione metabolism |
| AOC1 | Arginine and proline metabolism；Histidine metabolism；Tryptophan metabolism |
| AOC2 | beta-Alanine metabolism; Glycine, serine and threonine metabolism; Phenylalanine metabolism; Tyrosine metabolism |
| AOC3 | beta-Alanine metabolism; Glycine, serine and threonine metabolism; Phenylalanine metabolism; Tyrosine metabolism |
| AOX1 | Drug metabolism-cytochrome P450; Nicotinate and nicotinamide metabolism; Retinol metabolism; Tryptophan metabolism; Tyrosine metabolism; Valine, leucine and isoleucine degradation; Vitamin B6 metabolism |
| APIP | Cysteine and methionine metabolism |
| APRT | Purine metabolism |
| ARG1 | Arginine and proline metabolism; Arginine biosynthesis |
| ARG2 | Arginine and proline metabolism; Arginine biosynthesis |
| ARSA | Sphingolipid metabolism |
| ARSB | Glycosaminoglycan degradation |
| ASAH1 | Sphingolipid metabolism |
| ASAH2 | Sphingolipid metabolism |
| ASH1L | Lysine degradation |
| ASL | Alanine, aspartate and glutamate metabolism; Arginine biosynthesis |
| ASMT | Tryptophan metabolism |
| ASNS | Alanine, aspartate and glutamate metabolism |
| ASPA | Alanine, aspartate and glutamate metabolism; Histidine metabolism |
| ASPDH | Nicotinate and nicotinamide metabolism |
| ASS1 | Alanine, aspartate and glutamate metabolism; Arginine biosynthesis |
| ATIC | One carbon pool by folate; Purine metabolism |
| ATP12A | Oxidative phosphorylation |
| ATP4A | Oxidative phosphorylation |
| ATP4B | Oxidative phosphorylation |
| ATP5A1 | Oxidative phosphorylation |
| ATP5B | Oxidative phosphorylation |
| ATP5C1 | Oxidative phosphorylation |
| ATP5D | Oxidative phosphorylation |
| ATP5E | Oxidative phosphorylation |
| ATP5F1 | Oxidative phosphorylation |
| ATP5F1A | Oxidative phosphorylation |
| ATP5F1B | Oxidative phosphorylation |
| ATP5F1C | Oxidative phosphorylation |
| ATP5F1D | Oxidative phosphorylation |
| ATP5F1E | Oxidative phosphorylation |
| ATP5G1 | Oxidative phosphorylation |
| ATP5G2 | Oxidative phosphorylation |
| ATP5G3 | Oxidative phosphorylation |
| ATP5H | Oxidative phosphorylation |
| ATP5I | Oxidative phosphorylation |
| ATP5J | Oxidative phosphorylation |
| ATP5J2 | Oxidative phosphorylation |
| ATP5L | Oxidative phosphorylation |
| ATP5MC1 | Oxidative phosphorylation |
| ATP5MC1P5 | Oxidative phosphorylation |
| ATP5MC2 | Oxidative phosphorylation |
| ATP5MC3 | Oxidative phosphorylation |
| ATP5ME | Oxidative phosphorylation |
| ATP5MF | Oxidative phosphorylation |
| ATP5MG | Oxidative phosphorylation |
| ATP5O | Oxidative phosphorylation |
| ATP5PB | Oxidative phosphorylation |
| ATP5PD | Oxidative phosphorylation |
| ATP5PF | Oxidative phosphorylation |
| ATP5PO | Oxidative phosphorylation |
| ATP6AP1 | Oxidative phosphorylation |
| ATP6V0A1 | Oxidative phosphorylation |
| ATP6V0A2 | Oxidative phosphorylation |
| ATP6V0A4 | Oxidative phosphorylation |
| ATP6V0B | Oxidative phosphorylation |
| ATP6V0C | Oxidative phosphorylation |
| ATP6V0D1 | Oxidative phosphorylation |
| ATP6V0D2 | Oxidative phosphorylation |
| ATP6V0E1 | Oxidative phosphorylation |
| ATP6V0E2 | Oxidative phosphorylation |
| ATP6V1A | Oxidative phosphorylation |
| ATP6V1B1 | Oxidative phosphorylation |
| ATP6V1B2 | Oxidative phosphorylation |
| ATP6V1C1 | Oxidative phosphorylation |
| ATP6V1C2 | Oxidative phosphorylation |
| ATP6V1D | Oxidative phosphorylation |
| ATP6V1E1 | Oxidative phosphorylation |
| ATP6V1E2 | Oxidative phosphorylation |
| ATP6V1F | Oxidative phosphorylation |
| ATP6V1G1 | Oxidative phosphorylation |
| ATP6V1G2 | Oxidative phosphorylation |
| ATP6V1G3 | Oxidative phosphorylation |
| ATP6V1H | Oxidative phosphorylation |
| AUH | Valine, leucine and isoleucine degradation |
| AWAT2 | Retinol metabolism |
| AZIN2 | Arginine and proline metabolism |
| B3GALNT1 | glycosaminoglycan biosynthesis keratan sulfate；glycosphingolipid biosynthesis lacto and neolacto series |
| B3GALNT2 | glycosaminoglycan biosynthesis keratan sulfate；glycosphingolipid biosynthesis lacto and neolacto series |
| B3GALT1 | Glycosphingolipid biosynthesis-lacto and neolacto series |
| B3GALT2 | Glycosphingolipid biosynthesis-lacto and neolacto series |
| B3GALT4 | Glycosphingolipid biosynthesis-ganglio series |
| B3GALT5 | Glycosphingolipid biosynthesis-globo and isoglobo series; Glycosphingolipid biosynthesis-lacto and neolacto series |
| B3GALT6 | Glycosaminoglycan biosynthesis-chondroitin sulfate / dermatan sulfate; Glycosaminoglycan biosynthesis-heparan sulfate / heparin |
| B3GALTL | Other types of O-glycan biosynthesis |
| B3GAT1 | Mannose type O-glycan biosynthesis |
| B3GAT2 | Mannose type O-glycan biosynthesis |
| B3GAT3 | Glycosaminoglycan biosynthesis-chondroitin sulfate / dermatan sulfate; Glycosaminoglycan biosynthesis-heparan sulfate / heparin |
| B3GNT1 | Glycosaminoglycan biosynthesis-keratan sulfate; Glycosphingolipid biosynthesis-lacto and neolacto series |
| B3GNT2 | Glycosaminoglycan biosynthesis keratan sulfate；Glycosphingolipid biosynthesis lacto and neolacto series |
| B3GNT3 | Glycosphingolipid biosynthesis-lacto and neolacto series |
| B3GNT4 | Glycosphingolipid biosynthesis-lacto and neolacto series |
| B3GNT5 | Glycosphingolipid biosynthesis-lacto and neolacto series |
| B3GNT6 | Mannose type O-glycan biosynthesis; Mucin type O-glycan biosynthesis |
| B3GNT7 | Glycosaminoglycan biosynthesis-keratan sulfate |
| B4GALNT1 | Glycosphingolipid biosynthesis-ganglio series |
| B4GALNT3 | Various types of N-glycan biosynthesis |
| B4GALNT4 | Various types of N-glycan biosynthesis |
| B4GALT1 | Galactose metabolism; Glycosaminoglycan biosynthesis-keratan sulfate; Glycosphingolipid biosynthesis-lacto and neolacto series; Mannose type O-glycan biosynthesis; Other types of O-glycan biosynthesis; Various types of N-glycan biosynthesis |
| B4GALT2 | Galactose metabolism; Glycosaminoglycan biosynthesis-keratan sulfate; Glycosphingolipid biosynthesis-lacto and neolacto series; Mannose type O-glycan biosynthesis; Other types of O-glycan biosynthesis; Various types of N-glycan biosynthesis |
| B4GALT3 | Glycosaminoglycan biosynthesis-keratan sulfate; Glycosphingolipid biosynthesis-lacto and neolacto series; Mannose type O-glycan biosynthesis; Other types of O-glycan biosynthesis; Various types of N-glycan biosynthesis |
| B4GALT4 | Glycosaminoglycan biosynthesis-keratan sulfate; Glycosphingolipid biosynthesis-lacto and neolacto series |
| B4GALT5 | Mucin type O-glycan biosynthesis |
| B4GALT6 | Sphingolipid metabolism |
| B4GALT7 | Glycosaminoglycan biosynthesis-chondroitin sulfate / dermatan sulfate; Glycosaminoglycan biosynthesis-heparan sulfate / heparin |
| B4GAT1 | Glycosaminoglycan biosynthesis keratan sulfate；Glycosphingolipid biosynthesis lacto and neolacto series |
| BAAT | Biosynthesis of unsaturated fatty acids; Taurine and hypotaurine metabolism |
| BBOX1 | Lysine degradation |
| BCAT1 | Cysteine and methionine metabolism; Pantothenate and CoA biosynthesis; Valine, leucine and isoleucine biosynthesis; Valine, leucine and isoleucine degradation |
| BCAT2 | Cysteine and methionine metabolism; Pantothenate and CoA biosynthesis; Valine, leucine and isoleucine biosynthesis; Valine, leucine and isoleucine degradation |
| BCKDHA | Propanoate metabolism; Valine, leucine and isoleucine degradation |
| BCKDHB | Propanoate metabolism; Valine, leucine and isoleucine degradation |
| BCMO1 | Retinol metabolism |
| BCO1 | Retinol metabolism |
| BDH1 | histidine metabolism；selenoamino acid metabolism；tyrosine metabolism |
| BDH2 | drug metabolism other enzymes |
| BHMT | oxidative phosphorylation |
| BHMT2 | Cysteine and methionine metabolism |
| BLVRA | Porphyrin and chlorophyll metabolism |
| BLVRB | Porphyrin and chlorophyll metabolism; Riboflavin metabolism |
| BPGM | Glycine, serine and threonine metabolism; Glycolysis; Gluconeogenesis |
| BPNT1 | Sulfur metabolism |
| BRP44L | Gluconeogenesis |
| BST1 | Nicotinate and nicotinamide metabolism |
| BTD | Biotin metabolism |
| BUD23 | Histidine metabolism；Selenoamino acid metabolism；Tyrosine metabolism |
| C10orf129 | drug metabolism other enzymes |
| C12orf5 | oxidative phosphorylation |
| C14orf149 | pyrimidine metabolism |
| C14orf159 | retinol metabolism；drug metabolism cytochrome p450；drug metabolism other enzymes |
| C17orf48 | metabolism of xenobiotics by cytochrome p450；drug metabolism cytochrome p450；drug metabolism other enzymes；linoleic acid metabolism；retinol metabolism |
| C1GALT1 | Mucin type O-glycan biosynthesis |
| C1GALT1C1 | Mucin type O-glycan biosynthesis |
| C1orf57 | Purine metabolism; Thiamine metabolism |
| C1orf93 | Arachidonic acid metabolism |
| C2orf34 | Lysine degradation |
| C3orf39 | Mannose type O-glycan biosynthesis |
| C3orf64 | Other types of O-glycan biosynthesis |
| C5orf33 | Nicotinate and nicotinamide metabolism |
| C9orf103 | Pentose phosphate pathway |
| C9orf41 | Histidine metabolism |
| C9orf95 | Nicotinate and nicotinamide metabolism |
| C9orf98 | Purine metabolism; Thiamine metabolism |
| CA1 | Nitrogen metabolism |
| CA12 | Nitrogen metabolism |
| CA13 | Nitrogen metabolism |
| CA14 | Nitrogen metabolism |
| CA2 | Nitrogen metabolism |
| CA3 | Nitrogen metabolism |
| CA4 | Nitrogen metabolism |
| CA5A | Nitrogen metabolism |
| CA5B | Nitrogen metabolism |
| CA6 | Nitrogen metabolism |
| CA7 | Nitrogen metabolism |
| CA8 | Nitrogen metabolism |
| CA9 | Nitrogen metabolism |
| CAD | Alanine, aspartate and glutamate metabolism; Pyrimidine metabolism |
| CANT1 | Purine metabolism; Pyrimidine metabolism |
| CARNS1 | Arginine and proline metabolism; beta-Alanine metabolism; Histidine metabolism |
| CAT | Glyoxylate and dicarboxylate metabolism; Tryptophan metabolism |
| CBR1 | Arachidonic acid metabolism; Folate biosynthesis; Metabolism of xenobiotics by cytochrome P450 |
| CBR3 | Arachidonic acid metabolism; Metabolism of xenobiotics by cytochrome P450 |
| CBS | Cysteine and methionine metabolism; Cysteine and methionine metabolism; Glycine, serine and threonine metabolism; Glycine, serine and threonine metabolism |
| CCBL1 | Selenocompound metabolism; Tryptophan metabolism |
| CCBL2 | Selenocompound metabolism; Tryptophan metabolism |
| CD38 | Nicotinate and nicotinamide metabolism |
| CDA | Drug metabolism-other enzymes; Pyrimidine metabolism |
| CDIPT | Glycerophospholipid metabolism; Inositol phosphate metabolism |
| CDO1 | Cysteine and methionine metabolism; Taurine and hypotaurine metabolism |
| CDS1 | Glycerophospholipid metabolism |
| CDS2 | Glycerophospholipid metabolism |
| CECR1 | Purine metabolism |
| CEL | Glycerolipid metabolism; Glycerolipid metabolism |
| CEPT1 | Ether lipid metabolism; Glycerophospholipid metabolism; Phosphonate and phosphinate metabolism |
| CERK | Sphingolipid metabolism |
| CES1 | Drug metabolism-other enzymes |
| CES2 | Drug metabolism-other enzymes |
| CES5A | Drug metabolism other enzymes |
| CHAC1 | oxidative phosphorylation |
| CHAC2 | pyrimidine metabolism |
| CHAT | retinol metabolism；drug metabolism cytochrome p450；drug metabolism other enzymes |
| CHDH | metabolism of xenobiotics by cytochrome p450；drug metabolism cytochrome p450；drug metabolism other enzymes；linoleic acid metabolism；retinol metabolism |
| CHIA | cysteine and methionine metabolism |
| CHIT1 | fatty acid metabolism |
| CHKA | fatty acid metabolism |
| CHKB | porphyrin and chlorophyll metabolism；aminoacyl trna biosynthesis |
| CHPF | fructose and mannose metabolism；amino sugar and nucleotide sugar metabolism |
| CHPF2 | porphyrin and chlorophyll metabolism |
| CHPT1 | porphyrin and chlorophyll metabolism |
| CHST1 | Glycosaminoglycan biosynthesis-keratan sulfate |
| CHST10 | galactose metabolism；amino sugar and nucleotide sugar metabolism |
| CHST11 | o glycan biosynthesis |
| CHST12 | o glycan biosynthesis |
| CHST13 | Glycosaminoglycan biosynthesis-chondroitin sulfate / dermatan sulfate |
| CHST14 | Glycosaminoglycan biosynthesis-chondroitin sulfate / dermatan sulfate |
| CHST15 | Glycosaminoglycan biosynthesis-chondroitin sulfate / dermatan sulfate |
| CHST2 | Glycosaminoglycan biosynthesis-keratan sulfate |
| CHST3 | Glycosaminoglycan biosynthesis-chondroitin sulfate / dermatan sulfate |
| CHST4 | Glycosaminoglycan biosynthesis-keratan sulfate |
| CHST6 | Glycosaminoglycan biosynthesis-keratan sulfate |
| CHST7 | Glycosaminoglycan biosynthesis-chondroitin sulfate / dermatan sulfate |
| CHST8 | Various types of N-glycan biosynthesis |
| CHST9 | Various types of N-glycan biosynthesis |
| CHSY1 | Glycosaminoglycan biosynthesis-chondroitin sulfate / dermatan sulfate |
| CHSY3 | Glycosaminoglycan biosynthesis-chondroitin sulfate / dermatan sulfate |
| CKB | Arginine and proline metabolism |
| CKM | Arginine and proline metabolism |
| CKMT1A | Arginine and proline metabolism |
| CKMT1B | Arginine and proline metabolism |
| CKMT2 | Arginine and proline metabolism |
| CMAS | Amino sugar and nucleotide sugar metabolism |
| CMPK1 | Drug metabolism-other enzymes; Pyrimidine metabolism |
| CMPK2 | Pyrimidine metabolism |
| CNDP1 | Arginine and proline metabolism; beta-Alanine metabolism; Histidine metabolism |
| CNDP2 | Arginine and proline metabolism; beta-Alanine metabolism; Histidine metabolism |
| COASY | Pantothenate and CoA biosynthesis |
| COMT | Tyrosine metabolism |
| COQ2 | Ubiquinone and other terpenoid-quinone biosynthesis; Ubiquinone and other terpenoid-quinone biosynthesis |
| COQ3 | Ubiquinone and other terpenoid-quinone biosynthesis; Ubiquinone and other terpenoid-quinone biosynthesis |
| COQ5 | Ubiquinone and other terpenoid-quinone biosynthesis; Ubiquinone and other terpenoid-quinone biosynthesis |
| COQ6 | Ubiquinone and other terpenoid-quinone biosynthesis; Ubiquinone and other terpenoid-quinone biosynthesis |
| COQ7 | Ubiquinone and other terpenoid-quinone biosynthesis; Ubiquinone and other terpenoid-quinone biosynthesis |
| COX10 | Oxidative phosphorylation; Porphyrin and chlorophyll metabolism |
| COX11 | Oxidative phosphorylation |
| COX15 | Oxidative phosphorylation; Porphyrin and chlorophyll metabolism |
| COX17 | Oxidative phosphorylation |
| COX4I1 | Oxidative phosphorylation |
| COX4I2 | Oxidative phosphorylation |
| COX5A | Oxidative phosphorylation |
| COX5B | Oxidative phosphorylation |
| COX6A1 | Oxidative phosphorylation |
| COX6A2 | Oxidative phosphorylation |
| COX6B1 | Oxidative phosphorylation |
| COX6B2 | Oxidative phosphorylation |
| COX6C | Oxidative phosphorylation |
| COX6CP3 | Oxidative phosphorylation |
| COX7A1 | Oxidative phosphorylation |
| COX7A2 | Oxidative phosphorylation |
| COX7A2L | Oxidative phosphorylation |
| COX7B | Oxidative phosphorylation |
| COX7B2 | Oxidative phosphorylation |
| COX7C | Oxidative phosphorylation |
| COX8A | Oxidative phosphorylation |
| COX8C | Oxidative phosphorylation |
| CP | Porphyrin and chlorophyll metabolism |
| CPOX | Porphyrin and chlorophyll metabolism |
| CPS1 | Alanine, aspartate and glutamate metabolism; Arginine biosynthesis; Nitrogen metabolism |
| CPT1A | Fatty acid degradation |
| CPT1B | Fatty acid degradation |
| CPT1C | Fatty acid degradation |
| CPT2 | Fatty acid degradation |
| CRLS1 | Glycerophospholipid metabolism |
| CRYL1 | Pentose and glucuronate interconversions |
| CS | Glyoxylate and dicarboxylate metabolism; TCA cycle |
| CSAD | Taurine and hypotaurine metabolism |
| CSGALNACT1 | Glycosaminoglycan biosynthesis-chondroitin sulfate / dermatan sulfate |
| CSGALNACT2 | Glycosaminoglycan biosynthesis-chondroitin sulfate / dermatan sulfate |
| CTH | Cysteine and methionine metabolism; Glycine, serine and threonine metabolism; Selenocompound metabolism |
| CTPS | Pyrimidine metabolism |
| CTPS1 | Pyrimidine metabolism |
| CTPS2 | Pyrimidine metabolism |
| CYB5R1 | Amino sugar and nucleotide sugar metabolism |
| CYB5R2 | Amino sugar and nucleotide sugar metabolism |
| CYB5R3 | Amino sugar and nucleotide sugar metabolism |
| CYB5R4 | Amino sugar and nucleotide sugar metabolism |
| CYB5RL | Amino sugar and nucleotide sugar metabolism |
| CYC1 | Oxidative phosphorylation |
| CYP1A1 | Metabolism of xenobiotics by cytochrome P450; Retinol metabolism; Tryptophan metabolism |
| CYP1A2 | Drug metabolism-cytochrome P450; Linoleic acid metabolism; Metabolism of xenobiotics by cytochrome P450; Retinol metabolism; Tryptophan metabolism |
| CYP1B1 | Metabolism of xenobiotics by cytochrome P450; Tryptophan metabolism |
| CYP26A1 | Retinol metabolism |
| CYP26B1 | Retinol metabolism |
| CYP26C1 | Retinol metabolism |
| CYP27C1 | Retinol metabolism |
| CYP2A13 | Metabolism of xenobiotics by cytochrome P450 |
| CYP2A6 | Drug metabolism-cytochrome P450; Drug metabolism-other enzymes; Metabolism of xenobiotics by cytochrome P450; Retinol metabolism |
| CYP2A7 | Drug metabolism cytochrome P450；Drug metabolism other enzymes；Retinol metabolism |
| CYP2B6 | Arachidonic acid metabolism; Drug metabolism-cytochrome P450; Metabolism of xenobiotics by cytochrome P450; Retinol metabolism |
| CYP2C18 | Retinol metabolism |
| CYP2C19 | Arachidonic acid metabolism; Drug metabolism-cytochrome P450; Linoleic acid metabolism |
| CYP2C8 | Arachidonic acid metabolism; Drug metabolism-cytochrome P450; Linoleic acid metabolism; Retinol metabolism |
| CYP2C9 | Arachidonic acid metabolism; Drug metabolism-cytochrome P450; Linoleic acid metabolism; Metabolism of xenobiotics by cytochrome P450; Retinol metabolism |
| CYP2D6 | Drug metabolism-cytochrome P450; Drug metabolism-cytochrome P450; Metabolism of xenobiotics by cytochrome P450; Metabolism of xenobiotics by cytochrome P450 |
| CYP2D7 | Drug metabolism-cytochrome P450; Metabolism of xenobiotics by cytochrome P450 |
| CYP2E1 | Arachidonic acid metabolism; Drug metabolism-cytochrome P450; Drug metabolism-other enzymes; Linoleic acid metabolism; Metabolism of xenobiotics by cytochrome P450 |
| CYP2F1 | Metabolism of xenobiotics by cytochrome P450 |
| CYP2J2 | Arachidonic acid metabolism; Linoleic acid metabolism |
| CYP2S1 | Metabolism of xenobiotics by cytochrome P450; Retinol metabolism |
| CYP2U1 | Arachidonic acid metabolism |
| CYP3A4 | Drug metabolism-cytochrome P450; Drug metabolism-other enzymes; Linoleic acid metabolism; Metabolism of xenobiotics by cytochrome P450; Retinol metabolism |
| CYP3A43 | Metabolism of xenobiotics by cytochrome P450；Drug metabolism cytochrome P450；Drug metabolism other enzymes；Linoleic acid metabolism；Retinol metabolism |
| CYP3A5 | Drug metabolism-cytochrome P450; Metabolism of xenobiotics by cytochrome P450; Retinol metabolism |
| CYP3A7 | Retinol metabolism |
| CYP4A11 | Arachidonic acid metabolism; Fatty acid degradation; Retinol metabolism |
| CYP4A22 | Arachidonic acid metabolism; Fatty acid degradation; Retinol metabolism |
| CYP4F2 | Arachidonic acid metabolism |
| CYP4F3 | Arachidonic acid metabolism |
| CYP4F8 | Arachidonic acid metabolism |
| DAD1 | Various types of N-glycan biosynthesis |
| DAK | Fructose and mannose metabolism; Glycerolipid metabolism; Glycerolipid metabolism |
| DAO | Arginine and proline metabolism; D-Amino acid metabolism; Glycine, serine and threonine metabolism; Various types of N-glycan biosynthesis |
| DBH | Tyrosine metabolism |
| DBT | Propanoate metabolism; Valine, leucine and isoleucine degradation |
| DCI | Fatty acid degradation |
| DCK | Purine metabolism; Pyrimidine metabolism |
| DCT | Tyrosine metabolism |
| DCTD | Pyrimidine metabolism |
| DCTPP1 | Pyrimidine metabolism |
| DCXR | Pentose and glucuronate interconversions |
| DDC | Phenylalanine metabolism; Tryptophan metabolism; Tyrosine metabolism |
| DDO | Alanine, aspartate and glutamate metabolism; D-Amino acid metabolism |
| DDOST | Various types of N-glycan biosynthesis |
| DEGS1 | Sphingolipid metabolism |
| DEGS2 | Sphingolipid metabolism |
| DERA | Pentose phosphate pathway |
| DGAT1 | Glycerolipid metabolism; Glycerolipid metabolism; Retinol metabolism |
| DGAT2 | Glycerolipid metabolism; Glycerolipid metabolism |
| DGKA | Glycerolipid metabolism; Glycerolipid metabolism; Glycerophospholipid metabolism |
| DGKB | Glycerolipid metabolism; Glycerolipid metabolism; Glycerophospholipid metabolism |
| DGKD | Glycerolipid metabolism; Glycerolipid metabolism; Glycerophospholipid metabolism |
| DGKE | Glycerolipid metabolism; Glycerolipid metabolism; Glycerophospholipid metabolism |
| DGKG | Glycerolipid metabolism; Glycerolipid metabolism; Glycerophospholipid metabolism |
| DGKH | Glycerolipid metabolism; Glycerolipid metabolism; Glycerophospholipid metabolism |
| DGKI | Glycerolipid metabolism; Glycerolipid metabolism; Glycerophospholipid metabolism |
| DGKK | Glycerolipid metabolism; Glycerolipid metabolism; Glycerophospholipid metabolism |
| DGKQ | Glycerolipid metabolism; Glycerolipid metabolism; Glycerophospholipid metabolism |
| DGKZ | Glycerolipid metabolism; Glycerolipid metabolism; Glycerophospholipid metabolism |
| DGLUCY | D-Amino acid metabolism |
| DGUOK | Purine metabolism |
| DHDDS | Terpenoid backbone biosynthesis |
| DHDH | Metabolism of xenobiotics by cytochrome P450; Pentose and glucuronate interconversions |
| DHDPSL | Arginine and proline metabolism; Glyoxylate and dicarboxylate metabolism |
| DHFR | Folate biosynthesis; One carbon pool by folate |
| DHFRL1 | Folate biosynthesis; One carbon pool by folate |
| DHODH | Pyrimidine metabolism |
| DHRS3 | Retinol metabolism |
| DHRS4 | Retinol metabolism |
| DHRS4L1 | Retinol metabolism |
| DHRS4L2 | Retinol metabolism |
| DHRS9 | Retinol metabolism |
| DLAT | Glycolysis; Gluconeogenesis; Pyruvate metabolism; TCA cycle |
| DLD | Glycine, serine and threonine metabolism; Glycolysis; Gluconeogenesis; Glyoxylate and dicarboxylate metabolism; Propanoate metabolism; Pyruvate metabolism; TCA cycle; Valine, leucine and isoleucine degradation |
| DLST | Lysine degradation; TCA cycle |
| DMGDH | Glycine, serine and threonine metabolism |
| DNMT1 | Cysteine and methionine metabolism |
| DNMT3A | Cysteine and methionine metabolism |
| DNMT3B | Cysteine and methionine metabolism |
| DNMT3L | Cysteine and methionine metabolism |
| DOLK | N-Glycan biosynthesis |
| DOLPP1 | N-Glycan biosynthesis |
| DOT1L | Lysine degradation |
| DPAGT1 | N-Glycan biosynthesis |
| DPM1 | N-Glycan biosynthesis |
| DPM2 | Glycosylphosphatidylinositol (GPI)-anchor biosynthesis; N-Glycan biosynthesis |
| DPM3 | N-Glycan biosynthesis |
| DPYD | beta-Alanine metabolism; Drug metabolism-other enzymes; Pantothenate and CoA biosynthesis; Pyrimidine metabolism |
| DPYS | beta-Alanine metabolism; Drug metabolism-other enzymes; Pantothenate and CoA biosynthesis; Pyrimidine metabolism |
| DSE | Glycosaminoglycan biosynthesis-chondroitin sulfate / dermatan sulfate |
| DTYMK | Pyrimidine metabolism |
| DUT | Drug metabolism-other enzymes; Pyrimidine metabolism |
| EARS2 | Porphyrin and chlorophyll metabolism |
| ECHDC1 | Propanoate metabolism |
| ECHS1 | beta-Alanine metabolism; Butanoate metabolism; Fatty acid degradation; Fatty acid elongation; Lysine degradation; Propanoate metabolism; Tryptophan metabolism; Valine, leucine and isoleucine degradation |
| ECI1 | Fatty acid metabolism |
| ECI2 | Fatty acid metabolism |
| EHHADH | beta-Alanine metabolism; Butanoate metabolism; Fatty acid degradation; Lysine degradation; Propanoate metabolism; Tryptophan metabolism; Valine, leucine and isoleucine degradation |
| EHMT1 | Lysine degradation |
| EHMT2 | Lysine degradation |
| ELOVL1 | Fatty acid elongation |
| ELOVL2 | Biosynthesis of unsaturated fatty acids; Fatty acid elongation |
| ELOVL3 | Fatty acid elongation |
| ELOVL4 | Fatty acid elongation |
| ELOVL5 | Biosynthesis of unsaturated fatty acids; Fatty acid elongation |
| ELOVL6 | Biosynthesis of unsaturated fatty acids; Fatty acid elongation |
| ELOVL7 | Fatty acid elongation |
| ENGASE | Other glycan degradation |
| ENO1 | Glycolysis; Gluconeogenesis |
| ENO2 | Glycolysis; Gluconeogenesis |
| ENO3 | Glycolysis; Gluconeogenesis |
| ENOPH1 | Cysteine and methionine metabolism |
| ENOSF1 | Fructose and mannose metabolism |
| ENPP1 | Nicotinate and nicotinamide metabolism; Pantothenate and CoA biosynthesis; Purine metabolism; Pyrimidine metabolism; Riboflavin metabolism; Starch and sucrose metabolism |
| ENPP2 | Ether lipid metabolism |
| ENPP3 | Nicotinate and nicotinamide metabolism; Pantothenate and CoA biosynthesis; Purine metabolism; Pyrimidine metabolism; Riboflavin metabolism; Starch and sucrose metabolism |
| ENPP4 | Purine metabolism |
| ENPP6 | Ether lipid metabolism |
| ENPP7 | Sphingolipid metabolism |
| ENTPD1 | Purine metabolism; Pyrimidine metabolism |
| ENTPD2 | Purine metabolism |
| ENTPD3 | Purine metabolism; Pyrimidine metabolism |
| ENTPD4 | Purine metabolism; Pyrimidine metabolism |
| ENTPD5 | Purine metabolism; Pyrimidine metabolism |
| ENTPD6 | Purine metabolism; Pyrimidine metabolism |
| ENTPD8 | Purine metabolism; Pyrimidine metabolism |
| EPHX1 | Metabolism of xenobiotics by cytochrome P450 |
| EPHX2 | Arachidonic acid metabolism |
| EPRS | Porphyrin and chlorophyll metabolism |
| EPRS1 | Porphyrin and chlorophyll metabolism；Aminoacyl trna biosynthesis |
| EPT1 | Ether lipid metabolism; Glycerophospholipid metabolism; Phosphonate and phosphinate metabolism |
| ETHE1 | Sulfur metabolism |
| ETNK1 | Glycerophospholipid metabolism |
| ETNK2 | Glycerophospholipid metabolism |
| EXT1 | Glycosaminoglycan biosynthesis-heparan sulfate / heparin |
| EXT2 | Glycosaminoglycan biosynthesis-heparan sulfate / heparin |
| EXTL1 | Glycosaminoglycan biosynthesis-heparan sulfate / heparin |
| EXTL2 | Glycosaminoglycan biosynthesis-heparan sulfate / heparin |
| EXTL3 | Glycosaminoglycan biosynthesis-heparan sulfate / heparin |
| EZH1 | Lysine degradation |
| EZH2 | Lysine degradation |
| FADS1 | Biosynthesis of unsaturated fatty acids |
| FADS2 | alpha-Linolenic acid metabolism; Biosynthesis of unsaturated fatty acids |
| FAH | Tyrosine metabolism |
| FAHD1 | Tyrosine metabolism |
| FAM108A1 | Fatty acid elongation |
| FAM108B1 | Fatty acid elongation |
| FAM108C1 | Fatty acid elongation |
| FASN | Fatty acid biosynthesis |
| FBP1 | Fructose and mannose metabolism; Glycolysis; Gluconeogenesis; Pentose phosphate pathway |
| FBP2 | Fructose and mannose metabolism; Glycolysis; Gluconeogenesis; Pentose phosphate pathway |
| FCSK | Fructose and mannose metabolism；Amino sugar and nucleotide sugar metabolism |
| FDPS | Terpenoid backbone biosynthesis |
| FECH | Porphyrin and chlorophyll metabolism |
| FH | Pyruvate metabolism; TCA cycle |
| FHIT | Purine metabolism |
| FIG4 | Inositol phosphate metabolism |
| FKRP | Mannose type O-glycan biosynthesis |
| FKTN | Mannose type O-glycan biosynthesis |
| FLAD1 | Riboflavin metabolism |
| FMO1 | Drug metabolism-cytochrome P450 |
| FMO2 | Drug metabolism-cytochrome P450 |
| FMO3 | Drug metabolism-cytochrome P450 |
| FMO4 | Drug metabolism-cytochrome P450 |
| FMO5 | Drug metabolism-cytochrome P450 |
| FNTA | Terpenoid backbone biosynthesis |
| FNTB | Terpenoid backbone biosynthesis |
| FOLH1 | Alanine, aspartate and glutamate metabolism |
| FPGS | Folate biosynthesis |
| FPGT | Amino sugar and nucleotide sugar metabolism; Fructose and mannose metabolism |
| FTCD | Histidine metabolism; One carbon pool by folate |
| FTH1 | Porphyrin and chlorophyll metabolism |
| FTMT | Porphyrin and chlorophyll metabolism |
| FUCA1 | Other glycan degradation |
| FUCA2 | Other glycan degradation |
| FUK | Amino sugar and nucleotide sugar metabolism; Fructose and mannose metabolism |
| FUT1 | Glycosphingolipid biosynthesis-globo and isoglobo series; Glycosphingolipid biosynthesis-lacto and neolacto series |
| FUT2 | Glycosphingolipid biosynthesis-globo and isoglobo series; Glycosphingolipid biosynthesis-lacto and neolacto series |
| FUT3 | Glycosphingolipid biosynthesis-lacto and neolacto series |
| FUT4 | Glycosphingolipid biosynthesis-lacto and neolacto series; Mannose type O-glycan biosynthesis |
| FUT5 | Glycosphingolipid biosynthesis-lacto and neolacto series |
| FUT6 | Glycosphingolipid biosynthesis-lacto and neolacto series |
| FUT7 | Glycosphingolipid biosynthesis-lacto and neolacto series |
| FUT8 | Glycosaminoglycan biosynthesis-keratan sulfate; Various types of N-glycan biosynthesis |
| FUT9 | Glycosphingolipid biosynthesis-globo and isoglobo series; Glycosphingolipid biosynthesis-lacto and neolacto series; Mannose type O-glycan biosynthesis |
| FXN | Porphyrin and chlorophyll metabolism |
| G6PC1 | Starch and sucrose metabolism；Galactose metabolism；Glycolysis; Gluconeogenesis |
| G6PC2 | Galactose metabolism; Glycolysis; Gluconeogenesis; Starch and sucrose metabolism |
| G6PC3 | Galactose metabolism; Gluconeogenesis; Starch and sucrose metabolism |
| G6PD | Glutathione metabolism; Pentose phosphate pathway |
| GAA | Galactose metabolism; Starch and sucrose metabolism |
| GAD1 | Alanine, aspartate and glutamate metabolism; beta-Alanine metabolism; Butanoate metabolism; Taurine and hypotaurine metabolism |
| GAD2 | Alanine, aspartate and glutamate metabolism; beta-Alanine metabolism; Butanoate metabolism; Taurine and hypotaurine metabolism |
| GADL1 | beta-Alanine metabolism; Pantothenate and CoA biosynthesis; Taurine and hypotaurine metabolism |
| GAL3ST1 | Ether lipid metabolism; Sphingolipid metabolism |
| GALC | Sphingolipid metabolism |
| GALE | Amino sugar and nucleotide sugar metabolism; Galactose metabolism |
| GALK1 | Amino sugar and nucleotide sugar metabolism; Galactose metabolism |
| GALK2 | Galactose metabolism；Amino sugar and nucleotide sugar metabolism |
| GALM | Galactose metabolism; Glycolysis; Gluconeogenesis |
| GALNS | Glycosaminoglycan degradation |
| GALNT1 | Mucin type O-glycan biosynthesis |
| GALNT10 | Mucin type O-glycan biosynthesis |
| GALNT11 | Mucin type O-glycan biosynthesis |
| GALNT12 | Mucin type O-glycan biosynthesis |
| GALNT13 | Mucin type O-glycan biosynthesis |
| GALNT14 | Mucin type O-glycan biosynthesis |
| GALNT15 | O-Glycan biosynthesis |
| GALNT16 | O-Glycan biosynthesis |
| GALNT17 | O-Glycan biosynthesis |
| GALNT18 | O-Glycan biosynthesis |
| GALNT2 | Mucin type O-glycan biosynthesis |
| GALNT3 | Mucin type O-glycan biosynthesis |
| GALNT4 | Mucin type O-glycan biosynthesis |
| GALNT5 | Mucin type O-glycan biosynthesis |
| GALNT6 | Mucin type O-glycan biosynthesis |
| GALNT7 | Mucin type O-glycan biosynthesis |
| GALNT8 | Mucin type O-glycan biosynthesis |
| GALNT9 | Mucin type O-glycan biosynthesis |
| GALNTL2 | Mucin type O-glycan biosynthesis |
| GALNTL4 | Mucin type O-glycan biosynthesis |
| GALNTL5 | Mucin type O-glycan biosynthesis |
| GALNTL6 | Mucin type O-glycan biosynthesis |
| GALT | Amino sugar and nucleotide sugar metabolism; Galactose metabolism |
| GAMT | Arginine and proline metabolism; Glycine, serine and threonine metabolism |
| GANAB | N-Glycan biosynthesis |
| GANC | Galactose metabolism; Starch and sucrose metabolism |
| GAPDH | Glycolysis; Gluconeogenesis |
| GAPDHS | Glycolysis; Gluconeogenesis |
| GART | One carbon pool by folate; Purine metabolism |
| GATM | Arginine and proline metabolism; Glycine, serine and threonine metabolism |
| GBA | Other glycan degradation; Sphingolipid metabolism |
| GBA2 | Other glycan degradation; Sphingolipid metabolism |
| GBA3 | Starch and sucrose metabolism |
| GBE1 | Starch and sucrose metabolism |
| GBGT1 | Glycosphingolipid biosynthesis-globo and isoglobo series |
| GCAT | Glycine, serine and threonine metabolism |
| GCDH | Fatty acid degradation; Lysine degradation; Tryptophan metabolism |
| GCH1 | Folate biosynthesis |
| GCK | Amino sugar and nucleotide sugar metabolism; Galactose metabolism; Glycolysis; Gluconeogenesis; Starch and sucrose metabolism |
| GCLC | Cysteine and methionine metabolism; Glutathione metabolism |
| GCLM | Cysteine and methionine metabolism; Glutathione metabolism |
| GCNT1 | Mucin type O-glycan biosynthesis |
| GCNT2 | Glycosphingolipid biosynthesis-lacto and neolacto series |
| GCNT3 | Mucin type O-glycan biosynthesis |
| GCNT4 | Mucin type O-glycan biosynthesis |
| GCSH | Glycine, serine and threonine metabolism; Glyoxylate and dicarboxylate metabolism |
| GDA | Purine metabolism |
| GDPD1 | Ether lipid metabolism |
| GDPD3 | Ether lipid metabolism |
| GFPT1 | Alanine, aspartate and glutamate metabolism; Amino sugar and nucleotide sugar metabolism |
| GFPT2 | Alanine, aspartate and glutamate metabolism; Amino sugar and nucleotide sugar metabolism |
| GFUS | Amino sugar and nucleotide sugar metabolism；Fructose and mannose metabolism |
| GGCT | Glutathione metabolism |
| GGCX | Ubiquinone and other terpenoid-quinone biosynthesis; Ubiquinone and other terpenoid-quinone biosynthesis |
| GGH | Folate biosynthesis |
| GGPS1 | Terpenoid backbone biosynthesis |
| GGT1 | Arachidonic acid metabolism; Glutathione metabolism; Taurine and hypotaurine metabolism |
| GGT5 | Arachidonic acid metabolism; Glutathione metabolism; Taurine and hypotaurine metabolism |
| GGT6 | Glutathione metabolism; Taurine and hypotaurine metabolism |
| GGT7 | Glutathione metabolism; Taurine and hypotaurine metabolism |
| GK | Glycerolipid metabolism; Glycerolipid metabolism |
| GK2 | Glycerolipid metabolism; Glycerolipid metabolism |
| GLA | Galactose metabolism; Glycerolipid metabolism; Glycerolipid metabolism; Glycosphingolipid biosynthesis-globo and isoglobo series; Sphingolipid metabolism |
| GLB1 | Galactose metabolism; Glycosaminoglycan degradation; Glycosphingolipid biosynthesis-ganglio series; Other glycan degradation; Sphingolipid metabolism |
| GLCE | Glycosaminoglycan biosynthesis-heparan sulfate / heparin |
| GLDC | Glycine, serine and threonine metabolism; Glyoxylate and dicarboxylate metabolism |
| GLO1 | Pyruvate metabolism |
| GLS | Alanine, aspartate and glutamate metabolism; Arginine biosynthesis; D-Amino acid metabolism |
| GLS2 | Alanine, aspartate and glutamate metabolism; Arginine biosynthesis; D-Amino acid metabolism |
| GLT25D1 | Lysine degradation; Other types of O-glycan biosynthesis |
| GLT25D2 | Lysine degradation; Other types of O-glycan biosynthesis |
| GLT8D4 | Other types of O-glycan biosynthesis |
| GLUD1 | Alanine, aspartate and glutamate metabolism; Arginine biosynthesis; Nitrogen metabolism |
| GLUD2 | Alanine, aspartate and glutamate metabolism; Arginine biosynthesis; Nitrogen metabolism |
| GLUL | Alanine, aspartate and glutamate metabolism; Arginine biosynthesis; Glyoxylate and dicarboxylate metabolism; Nitrogen metabolism |
| GLYAT | Phenylalanine metabolism |
| GLYCTK | Glycerolipid metabolism; Glycerolipid metabolism; Glycine, serine and threonine metabolism; Glyoxylate and dicarboxylate metabolism; Pentose phosphate pathway |
| GMDS | Amino sugar and nucleotide sugar metabolism; Fructose and mannose metabolism |
| GMPPA | Amino sugar and nucleotide sugar metabolism; Fructose and mannose metabolism |
| GMPPB | Amino sugar and nucleotide sugar metabolism; Fructose and mannose metabolism |
| GMPR | Purine metabolism |
| GMPR2 | Purine metabolism |
| GMPS | Drug metabolism-other enzymes; Purine metabolism |
| GNE | Amino sugar and nucleotide sugar metabolism |
| GNMT | Glycine, serine and threonine metabolism |
| GNPAT | Glycerophospholipid metabolism |
| GNPDA1 | Amino sugar and nucleotide sugar metabolism |
| GNPDA2 | Amino sugar and nucleotide sugar metabolism |
| GNPNAT1 | Amino sugar and nucleotide sugar metabolism |
| GNS | Glycosaminoglycan degradation |
| GOT1 | Alanine, aspartate and glutamate metabolism; Arginine and proline metabolism; Arginine biosynthesis; Cysteine and methionine metabolism; Gluconeogenesis; Phenylalanine metabolism; Phenylalanine, tyrosine and tryptophan biosynthesis; Tyrosine metabolism |
| GOT2 | Alanine, aspartate and glutamate metabolism; Arginine and proline metabolism; Arginine biosynthesis; Cysteine and methionine metabolism; Gluconeogenesis; Phenylalanine metabolism; Phenylalanine, tyrosine and tryptophan biosynthesis; Tyrosine metabolism |
| GPAA1 | Glycosylphosphatidylinositol (GPI)-anchor biosynthesis |
| GPAM | Glycerolipid metabolism; Glycerolipid metabolism; Glycerophospholipid metabolism |
| GPAT2 | Glycerolipid metabolism; Glycerolipid metabolism; Glycerophospholipid metabolism |
| GPAT3 | Glycerolipid metabolism；Glycerophospholipid metabolism |
| GPAT4 | Glycerolipid metabolism；Glycerophospholipid metabolism |
| GPCPD1 | Glycerophospholipid metabolism |
| GPD1 | Glycerophospholipid metabolism |
| GPD1L | Glycerophospholipid metabolism |
| GPD2 | Glycerophospholipid metabolism |
| GPHN | Folate biosynthesis |
| GPI | Amino sugar and nucleotide sugar metabolism; Gluconeogenesis; Glycolysis; Gluconeogenesis; Pentose phosphate pathway; Starch and sucrose metabolism |
| GPLD1 | Glycosylphosphatidylinositol (GPI)-anchor biosynthesis |
| GPT | Alanine, aspartate and glutamate metabolism; Arginine biosynthesis |
| GPT2 | Alanine, aspartate and glutamate metabolism; Arginine biosynthesis |
| GPX1 | Arachidonic acid metabolism; Glutathione metabolism |
| GPX2 | Arachidonic acid metabolism; Glutathione metabolism |
| GPX3 | Arachidonic acid metabolism; Glutathione metabolism |
| GPX4 | Glutathione metabolism |
| GPX5 | Arachidonic acid metabolism; Glutathione metabolism |
| GPX6 | Arachidonic acid metabolism; Glutathione metabolism |
| GPX7 | Arachidonic acid metabolism; Glutathione metabolism |
| GPX8 | Arachidonic acid metabolism; Glutathione metabolism |
| GRHPR | Glycine, serine and threonine metabolism; Glyoxylate and dicarboxylate metabolism; Pyruvate metabolism |
| GSR | Glutathione metabolism |
| GSS | Cysteine and methionine metabolism; Glutathione metabolism |
| GSTA1 | Drug metabolism-cytochrome P450; Drug metabolism-other enzymes; Glutathione metabolism; Metabolism of xenobiotics by cytochrome P450 |
| GSTA2 | Drug metabolism-cytochrome P450; Drug metabolism-other enzymes; Glutathione metabolism; Metabolism of xenobiotics by cytochrome P450 |
| GSTA3 | Drug metabolism-cytochrome P450; Drug metabolism-other enzymes; Glutathione metabolism; Metabolism of xenobiotics by cytochrome P450 |
| GSTA4 | Drug metabolism-cytochrome P450; Drug metabolism-other enzymes; Glutathione metabolism; Metabolism of xenobiotics by cytochrome P450 |
| GSTA5 | Drug metabolism-cytochrome P450; Drug metabolism-other enzymes; Glutathione metabolism; Metabolism of xenobiotics by cytochrome P450 |
| GSTK1 | Drug metabolism-cytochrome P450; Glutathione metabolism; Metabolism of xenobiotics by cytochrome P450 |
| GSTM1 | Drug metabolism-cytochrome P450; Drug metabolism-other enzymes; Glutathione metabolism; Metabolism of xenobiotics by cytochrome P450 |
| GSTM2 | Drug metabolism-cytochrome P450; Drug metabolism-other enzymes; Glutathione metabolism; Metabolism of xenobiotics by cytochrome P450 |
| GSTM3 | Drug metabolism-cytochrome P450; Drug metabolism-other enzymes; Glutathione metabolism; Metabolism of xenobiotics by cytochrome P450 |
| GSTM4 | Drug metabolism-cytochrome P450; Drug metabolism-other enzymes; Glutathione metabolism; Metabolism of xenobiotics by cytochrome P450 |
| GSTM5 | Drug metabolism-cytochrome P450; Drug metabolism-other enzymes; Glutathione metabolism; Metabolism of xenobiotics by cytochrome P450 |
| GSTO1 | Drug metabolism-cytochrome P450; Drug metabolism-other enzymes; Glutathione metabolism; Metabolism of xenobiotics by cytochrome P450 |
| GSTO2 | Drug metabolism-cytochrome P450; Drug metabolism-other enzymes; Glutathione metabolism; Metabolism of xenobiotics by cytochrome P450 |
| GSTP1 | Drug metabolism-cytochrome P450; Drug metabolism-other enzymes; Glutathione metabolism; Metabolism of xenobiotics by cytochrome P450 |
| GSTT1 | Drug metabolism-cytochrome P450; Drug metabolism-other enzymes; Glutathione metabolism; Metabolism of xenobiotics by cytochrome P450 |
| GSTT2 | Drug metabolism-cytochrome P450; Drug metabolism-other enzymes; Glutathione metabolism; Metabolism of xenobiotics by cytochrome P450 |
| GSTT2B | Drug metabolism-cytochrome P450; Drug metabolism-other enzymes; Glutathione metabolism; Metabolism of xenobiotics by cytochrome P450 |
| GSTZ1 | Tyrosine metabolism |
| GUCY1A1 | Purine metabolism |
| GUCY1A2 | Purine metabolism |
| GUCY1A3 | Purine metabolism |
| GUCY1B1 | Purine metabolism |
| GUCY1B3 | Purine metabolism |
| GUCY2C | Purine metabolism |
| GUCY2D | Purine metabolism |
| GUCY2F | Purine metabolism |
| GUK1 | Purine metabolism |
| GUSB | Drug metabolism-other enzymes; Glycosaminoglycan degradation; Pentose and glucuronate interconversions; Porphyrin and chlorophyll metabolism |
| GXYLT1 | Other types of O-glycan biosynthesis |
| GYG1 | Starch and sucrose metabolism |
| GYG2 | Starch and sucrose metabolism |
| GYLTL1B | Mannose type O-glycan biosynthesis |
| GYS1 | Starch and sucrose metabolism |
| GYS2 | Starch and sucrose metabolism |
| H6PD | Pentose phosphate pathway |
| HAAO | Tryptophan metabolism |
| HACD1 | Biosynthesis of unsaturated fatty acids |
| HACD2 | Biosynthesis of unsaturated fatty acids |
| HADH | Butanoate metabolism; Fatty acid degradation; Fatty acid elongation; Lysine degradation; Tryptophan metabolism; Valine, leucine and isoleucine degradation |
| HADHA | beta-Alanine metabolism; Biosynthesis of unsaturated fatty acids; Butanoate metabolism; Fatty acid degradation; Fatty acid elongation; Lysine degradation; Propanoate metabolism; Tryptophan metabolism; Valine, leucine and isoleucine degradation |
| HADHB | Fatty acid degradation; Fatty acid elongation; Valine, leucine and isoleucine degradation |
| HAGH | Pyruvate metabolism |
| HAGHL | Pyruvate metabolism |
| HAL | Histidine metabolism |
| HAO1 | Glyoxylate and dicarboxylate metabolism |
| HAO2 | Glyoxylate and dicarboxylate metabolism |
| HCCS | Porphyrin and chlorophyll metabolism |
| HDC | Histidine metabolism |
| HDDC3 | Purine metabolism |
| HEMK1 | Histidine metabolism；Selenoamino acid metabolism；Tyrosine metabolism |
| HEPH | Porphyrin and chlorophyll metabolism |
| HEXA | Amino sugar and nucleotide sugar metabolism; Glycosaminoglycan degradation; Glycosphingolipid biosynthesis-ganglio series; Glycosphingolipid biosynthesis-globo and isoglobo series; Other glycan degradation; Various types of N-glycan biosynthesis |
| HEXB | Amino sugar and nucleotide sugar metabolism; Glycosaminoglycan degradation; Glycosphingolipid biosynthesis-ganglio series; Glycosphingolipid biosynthesis-globo and isoglobo series; Other glycan degradation; Various types of N-glycan biosynthesis |
| HEXD | Various types of N-glycan biosynthesis |
| HEXDC | Other glycan degradation |
| HGD | Tyrosine metabolism |
| HGSNAT | Glycosaminoglycan degradation |
| HIBADH | Valine, leucine and isoleucine degradation |
| HIBCH | beta-Alanine metabolism; Propanoate metabolism; Valine, leucine and isoleucine degradation |
| HK1 | Amino sugar and nucleotide sugar metabolism; Fructose and mannose metabolism; Galactose metabolism; Glycolysis; Gluconeogenesis; Starch and sucrose metabolism |
| HK2 | Amino sugar and nucleotide sugar metabolism; Fructose and mannose metabolism; Galactose metabolism; Glycolysis; Gluconeogenesis; Starch and sucrose metabolism |
| HK3 | Amino sugar and nucleotide sugar metabolism; Fructose and mannose metabolism; Galactose metabolism; Glycolysis; Gluconeogenesis; Starch and sucrose metabolism |
| HKDC1 | Amino sugar and nucleotide sugar metabolism; Fructose and mannose metabolism; Galactose metabolism; Glycolysis; Gluconeogenesis; Starch and sucrose metabolism |
| HLCS | Biotin metabolism |
| HMBS | Porphyrin and chlorophyll metabolism |
| HMGCL | Butanoate metabolism; Valine, leucine and isoleucine degradation |
| HMGCLL1 | Butanoate metabolism; Valine, leucine and isoleucine degradation |
| HMGCR | Terpenoid backbone biosynthesis |
| HMGCS1 | Butanoate metabolism; Terpenoid backbone biosynthesis; Valine, leucine and isoleucine degradation |
| HMGCS2 | Butanoate metabolism; Terpenoid backbone biosynthesis; Valine, leucine and isoleucine degradation |
| HMOX1 | Porphyrin and chlorophyll metabolism |
| HMOX2 | Porphyrin and chlorophyll metabolism |
| HNMT | Histidine metabolism |
| HPD | Phenylalanine metabolism; Tyrosine metabolism; Ubiquinone and other terpenoid-quinone biosynthesis; Ubiquinone and other terpenoid-quinone biosynthesis |
| HPGDS | Arachidonic acid metabolism; Drug metabolism-cytochrome P450; Glutathione metabolism; Metabolism of xenobiotics by cytochrome P450 |
| HPRT1 | Drug metabolism-other enzymes; Purine metabolism |
| HPSE | Glycosaminoglycan degradation |
| HPSE2 | Glycosaminoglycan degradation |
| HS2ST1 | Glycosaminoglycan biosynthesis-heparan sulfate / heparin |
| HS3ST1 | Glycosaminoglycan biosynthesis-heparan sulfate / heparin |
| HS3ST2 | Glycosaminoglycan biosynthesis-heparan sulfate / heparin |
| HS3ST3A1 | Glycosaminoglycan biosynthesis-heparan sulfate / heparin |
| HS3ST3B1 | Glycosaminoglycan biosynthesis-heparan sulfate / heparin |
| HS3ST5 | Glycosaminoglycan biosynthesis-heparan sulfate / heparin |
| HS6ST1 | Glycosaminoglycan biosynthesis-heparan sulfate / heparin |
| HS6ST2 | Glycosaminoglycan biosynthesis-heparan sulfate / heparin |
| HS6ST3 | Glycosaminoglycan biosynthesis-heparan sulfate / heparin |
| HSD11B1 | Metabolism of xenobiotics by cytochrome P450 |
| HSD17B10 | Valine, leucine and isoleucine degradation |
| HSD17B12 | Biosynthesis of unsaturated fatty acids; Fatty acid elongation |
| HSD17B6 | Retinol metabolism |
| HYAL1 | Glycosaminoglycan degradation |
| HYAL2 | Glycosaminoglycan degradation |
| HYAL3 | Glycosaminoglycan degradation |
| HYAL4 | Glycosaminoglycan degradation |
| HYI | Glyoxylate and dicarboxylate metabolism |
| ICMT | Terpenoid backbone biosynthesis |
| IDH1 | Glutathione metabolism; TCA cycle |
| IDH2 | Glutathione metabolism; TCA cycle |
| IDH3A | TCA cycle |
| IDH3B | TCA cycle |
| IDH3G | TCA cycle |
| IDI1 | Terpenoid backbone biosynthesis |
| IDI2 | Terpenoid backbone biosynthesis |
| IDO1 | Tryptophan metabolism |
| IDO2 | Tryptophan metabolism |
| IDS | Glycosaminoglycan degradation |
| IDUA | Glycosaminoglycan degradation |
| IL4I1 | Alanine, aspartate and glutamate metabolism; Cysteine and methionine metabolism; Phenylalanine metabolism; Phenylalanine, tyrosine and tryptophan biosynthesis; Tryptophan metabolism; Tyrosine metabolism; Valine, leucine and isoleucine degradation |
| IMPA1 | Inositol phosphate metabolism |
| IMPA2 | Inositol phosphate metabolism |
| IMPAD1 | Inositol phosphate metabolism; Sulfur metabolism |
| IMPDH1 | Drug metabolism-other enzymes; Purine metabolism |
| IMPDH2 | Drug metabolism-other enzymes; Purine metabolism |
| INMT | Selenocompound metabolism; Tryptophan metabolism |
| INPP1 | Inositol phosphate metabolism |
| INPP4A | Inositol phosphate metabolism |
| INPP4B | Inositol phosphate metabolism |
| INPP5A | Inositol phosphate metabolism |
| INPP5B | Inositol phosphate metabolism |
| INPP5D | Inositol phosphate metabolism |
| INPP5E | Inositol phosphate metabolism |
| INPP5F | Inositol phosphate metabolism |
| INPP5J | Inositol phosphate metabolism |
| INPP5K | Inositol phosphate metabolism |
| INPPL1 | Inositol phosphate metabolism |
| IPMK | Inositol phosphate metabolism |
| IPPK | Inositol phosphate metabolism |
| ISPD | Mannose type O-glycan biosynthesis; Pentose and glucuronate interconversions |
| ISYNA1 | Inositol phosphate metabolism |
| ITGB1BP3 | Nicotinate and nicotinamide metabolism |
| ITPA | Drug metabolism-other enzymes; Purine metabolism |
| ITPK1 | Inositol phosphate metabolism |
| ITPKA | Inositol phosphate metabolism |
| ITPKB | Inositol phosphate metabolism |
| ITPKC | Inositol phosphate metabolism |
| IVD | Valine, leucine and isoleucine degradation |
| JMJD7-PLA2G4B | Alpha linolenic acid metabolism；Arachidonic acid metabolism；Ether lipid metabolism；Glycerophospholipid metabolism；Linoleic acid metabolism |
| KDSR | Sphingolipid metabolism |
| KHK | Fructose and mannose metabolism |
| KL | Pentose and glucuronate interconversions |
| KMO | Tryptophan metabolism |
| KMT2B | Lysine degradation |
| KMT5A | Lysine degradation |
| KMT5B | Lysine degradation |
| KMT5C | Lysine degradation |
| KTELC1 | Other types of O-glycan biosynthesis |
| KYNU | Tryptophan metabolism |
| L2HGDH | Butanoate metabolism |
| LALBA | Galactose metabolism |
| LAP3 | Arginine and proline metabolism; Glutathione metabolism |
| LARGE | Mannose type O-glycan biosynthesis |
| LASS1 | Sphingolipid metabolism |
| LASS2 | Sphingolipid metabolism |
| LASS3 | Sphingolipid metabolism |
| LASS4 | Sphingolipid metabolism |
| LASS5 | Sphingolipid metabolism |
| LASS6 | Sphingolipid metabolism |
| LCAT | Glycerophospholipid metabolism |
| LCLAT1 | Glycerolipid metabolism; Glycerolipid metabolism; Glycerophospholipid metabolism |
| LCMT1 | Histidine metabolism；Selenoamino acid metabolism；Tyrosine metabolism |
| LCMT2 | Histidine metabolism；Selenoamino acid metabolism；Tyrosine metabolism |
| LCT | Galactose metabolism |
| LDHA | Cysteine and methionine metabolism; Glycolysis; Gluconeogenesis; Propanoate metabolism; Pyruvate metabolism |
| LDHAL6A | Cysteine and methionine metabolism;  Glycolysis; Gluconeogenesis; Propanoate metabolism; Pyruvate metabolism |
| LDHAL6B | Cysteine and methionine metabolism; Glycolysis; Gluconeogenesis; Propanoate metabolism; Pyruvate metabolism |
| LDHB | Cysteine and methionine metabolism; Glycolysis; Gluconeogenesis; Propanoate metabolism; Pyruvate metabolism |
| LDHC | Cysteine and methionine metabolism; Glycolysis; Gluconeogenesis; Propanoate metabolism; Pyruvate metabolism |
| LDHD | Pyruvate metabolism |
| LFNG | Other types of O-glycan biosynthesis |
| LHPP | Oxidative phosphorylation |
| LIAS | Lipoic acid metabolism |
| LIPC | Glycerolipid metabolism; Glycerolipid metabolism |
| LIPF | Glycerolipid metabolism; Glycerolipid metabolism |
| LIPG | Glycerolipid metabolism; Glycerolipid metabolism |
| LIPT1 | Lipoic acid metabolism |
| LIPT2 | Lipoic acid metabolism |
| LPCAT1 | Ether lipid metabolism; Glycerophospholipid metabolism |
| LPCAT2 | Ether lipid metabolism; Glycerophospholipid metabolism |
| LPCAT3 | Glycerophospholipid metabolism |
| LPCAT4 | Ether lipid metabolism; Glycerophospholipid metabolism |
| LPGAT1 | Glycerophospholipid metabolism |
| LPIN1 | Glycerolipid metabolism; Glycerolipid metabolism; Glycerophospholipid metabolism |
| LPIN2 | Glycerolipid metabolism; Glycerolipid metabolism; Glycerophospholipid metabolism |
| LPIN3 | Glycerolipid metabolism; Glycerolipid metabolism; Glycerophospholipid metabolism |
| LPL | Glycerolipid metabolism; Glycerolipid metabolism |
| LRAT | Retinol metabolism |
| LRTOMT | Tyrosine metabolism |
| LTA4H | Arachidonic acid metabolism |
| LTC4S | Arachidonic acid metabolism |
| LYPLA1 | Glycerophospholipid metabolism |
| LYPLA2 | Glycerophospholipid metabolism |
| MAGT1 | Various types of N-glycan biosynthesis |
| MAN1A1 | Various types of N-glycan biosynthesis |
| MAN1A2 | Various types of N-glycan biosynthesis |
| MAN1B1 | Various types of N-glycan biosynthesis |
| MAN1C1 | Various types of N-glycan biosynthesis |
| MAN2A1 | Various types of N-glycan biosynthesis |
| MAN2A2 | Various types of N-glycan biosynthesis |
| MAN2B1 | Other glycan degradation |
| MAN2B2 | Other glycan degradation |
| MAN2C1 | Other glycan degradation |
| MANBA | Other glycan degradation |
| MAOA | Arginine and proline metabolism; Drug metabolism-cytochrome P450; Glycine, serine and threonine metabolism; Histidine metabolism; Phenylalanine metabolism; Tryptophan metabolism; Tyrosine metabolism |
| MAOB | Arginine and proline metabolism; Drug metabolism-cytochrome P450; Glycine, serine and threonine metabolism; Histidine metabolism; Phenylalanine metabolism; Tryptophan metabolism; Tyrosine metabolism |
| MARS | Selenocompound metabolism |
| MARS2 | Selenocompound metabolism |
| MAT1A | Cysteine and methionine metabolism |
| MAT2A | Cysteine and methionine metabolism |
| MAT2B | Cysteine and methionine metabolism |
| MBOAT1 | Glycerolipid metabolism; Glycerolipid metabolism; Glycerophospholipid metabolism |
| MBOAT2 | Glycerolipid metabolism; Glycerolipid metabolism; Glycerophospholipid metabolism |
| MBOAT7 | Glycerophospholipid metabolism |
| MCAT | Fatty acid biosynthesis |
| MCCC1 | Valine, leucine and isoleucine degradation |
| MCCC2 | Valine, leucine and isoleucine degradation |
| MCEE | Glyoxylate and dicarboxylate metabolism; Propanoate metabolism; Valine, leucine and isoleucine degradation |
| MDH1 | Cysteine and methionine metabolism; Glyoxylate and dicarboxylate metabolism; Pyruvate metabolism; TCA cycle |
| MDH2 | Cysteine and methionine metabolism; Gluconeogenesis; Glyoxylate and dicarboxylate metabolism; Pyruvate metabolism; TCA cycle |
| ME1 | Pyruvate metabolism |
| ME2 | Pyruvate metabolism |
| ME3 | Pyruvate metabolism |
| MECR | Fatty acid elongation |
| METTL2B | Histidine metabolism；Selenoamino acid metabolism；Tyrosine metabolism |
| METTL6 | Histidine metabolism；Selenoamino acid metabolism；Tyrosine metabolism |
| MFNG | Other types of O-glycan biosynthesis |
| MGAM | Galactose metabolism; Starch and sucrose metabolism |
| MGAM2 | Galactose metabolism; Starch and sucrose metabolism |
| MGAT1 | Various types of N-glycan biosynthesis |
| MGAT2 | Various types of N-glycan biosynthesis |
| MGAT3 | N-Glycan biosynthesis |
| MGAT4A | Various types of N-glycan biosynthesis |
| MGAT4B | Various types of N-glycan biosynthesis |
| MGAT4C | Various types of N-glycan biosynthesis |
| MGAT4D | Various types of N-glycan biosynthesis |
| MGAT5 | N-Glycan biosynthesis |
| MGAT5B | Mannose type O-glycan biosynthesis; N-Glycan biosynthesis |
| MGLL | Glycerolipid metabolism; Glycerolipid metabolism |
| MGST1 | Drug metabolism-cytochrome P450; Drug metabolism-other enzymes; Glutathione metabolism; Metabolism of xenobiotics by cytochrome P450 |
| MGST2 | Drug metabolism-cytochrome P450; Drug metabolism-other enzymes; Glutathione metabolism; Metabolism of xenobiotics by cytochrome P450 |
| MGST3 | Drug metabolism-cytochrome P450; Drug metabolism-other enzymes; Glutathione metabolism; Metabolism of xenobiotics by cytochrome P450 |
| MIF | Phenylalanine metabolism; Tyrosine metabolism |
| MINPP1 | Inositol phosphate metabolism |
| MIOX | Ascorbate and aldarate metabolism; Inositol phosphate metabolism |
| MLL | Lysine degradation |
| MLL2 | Lysine degradation |
| MLL3 | Lysine degradation |
| MLL5 | Lysine degradation |
| MLYCD | beta-Alanine metabolism; Propanoate metabolism |
| MMAB | Porphyrin and chlorophyll metabolism |
| MMUT | Propanoate metabolism；Valine leucine and isoleucine degradation |
| MOCOS | Folate biosynthesis |
| MOCS1 | Folate biosynthesis |
| MOCS2 | Folate biosynthesis |
| MOGAT1 | Glycerolipid metabolism; Glycerolipid metabolism |
| MOGAT2 | Glycerolipid metabolism; Glycerolipid metabolism |
| MOGAT3 | Glycerolipid metabolism; Glycerolipid metabolism |
| MOGS | N-Glycan biosynthesis |
| MPI | Amino sugar and nucleotide sugar metabolism; Fructose and mannose metabolism |
| MPO | Drug metabolism-other enzymes |
| MPST | Cysteine and methionine metabolism; Sulfur metabolism |
| MRI1 | Cysteine and methionine metabolism |
| MTAP | Cysteine and methionine metabolism |
| MT-ATP6 | Oxidative phosphorylation |
| MT-ATP8 | Oxidative phosphorylation |
| MT-CO1 | Oxidative phosphorylation |
| MT-CO2 | Oxidative phosphorylation |
| MT-CO3 | Oxidative phosphorylation |
| MT-CYB | Oxidative phosphorylation |
| MTFMT | One carbon pool by folate |
| MTHFD1 | One carbon pool by folate |
| MTHFD1L | One carbon pool by folate |
| MTHFD2 | One carbon pool by folate |
| MTHFD2L | One carbon pool by folate |
| MTHFR | One carbon pool by folate |
| MTHFS | One carbon pool by folate |
| MTM1 | Inositol phosphate metabolism |
| MTMR1 | Inositol phosphate metabolism |
| MTMR14 | Inositol phosphate metabolism |
| MTMR2 | Inositol phosphate metabolism |
| MTMR3 | Inositol phosphate metabolism |
| MTMR4 | Inositol phosphate metabolism |
| MTMR6 | Inositol phosphate metabolism |
| MTMR7 | Inositol phosphate metabolism |
| MTMR8 | Inositol phosphate metabolism |
| MT-ND1 | Oxidative phosphorylation |
| MT-ND2 | Oxidative phosphorylation |
| MT-ND3 | Oxidative phosphorylation |
| MT-ND4 | Oxidative phosphorylation |
| MT-ND4L | Oxidative phosphorylation |
| MT-ND5 | Oxidative phosphorylation |
| MT-ND6 | Oxidative phosphorylation |
| MTR | Cysteine and methionine metabolism; One carbon pool by folate; Selenocompound metabolism |
| MUT | Glyoxylate and dicarboxylate metabolism; Propanoate metabolism; Valine, leucine and isoleucine degradation |
| MVD | Terpenoid backbone biosynthesis |
| MVK | Terpenoid backbone biosynthesis |
| NAA80 | Limonene and pinene degradation；Phenylalanine metabolism；Tyrosine metabolism |
| NADK | Nicotinate and nicotinamide metabolism |
| NADSYN1 | Nicotinate and nicotinamide metabolism |
| NAGA | Glycosphingolipid biosynthesis-globo and isoglobo series |
| NAGK | Amino sugar and nucleotide sugar metabolism |
| NAGLU | Glycosaminoglycan degradation |
| NAGS | Arginine biosynthesis |
| NAMPT | Nicotinate and nicotinamide metabolism |
| NANP | Amino sugar and nucleotide sugar metabolism |
| NANS | Amino sugar and nucleotide sugar metabolism |
| NAPRT | Nicotinate and nicotinamide metabolism |
| NAT1 | Drug metabolism-other enzymes |
| NAT2 | Drug metabolism-other enzymes |
| NAT8 | Glutathione metabolism |
| NAT8B | Glutathione metabolism |
| NAT8L | Alanine, aspartate and glutamate metabolism |
| NDST1 | Glycosaminoglycan biosynthesis-heparan sulfate / heparin |
| NDST2 | Glycosaminoglycan biosynthesis-heparan sulfate / heparin |
| NDST3 | Glycosaminoglycan biosynthesis-heparan sulfate / heparin |
| NDST4 | Glycosaminoglycan biosynthesis-heparan sulfate / heparin |
| NDUFA1 | Oxidative phosphorylation |
| NDUFA10 | Oxidative phosphorylation |
| NDUFA11 | Oxidative phosphorylation |
| NDUFA12 | Oxidative phosphorylation |
| NDUFA13 | Oxidative phosphorylation |
| NDUFA2 | Oxidative phosphorylation |
| NDUFA3 | Oxidative phosphorylation |
| NDUFA4 | Oxidative phosphorylation |
| NDUFA4L2 | Oxidative phosphorylation |
| NDUFA5 | Oxidative phosphorylation |
| NDUFA6 | Oxidative phosphorylation |
| NDUFA7 | Oxidative phosphorylation |
| NDUFA8 | Oxidative phosphorylation |
| NDUFA9 | Oxidative phosphorylation |
| NDUFAB1 | Oxidative phosphorylation |
| NDUFB1 | Oxidative phosphorylation |
| NDUFB10 | Oxidative phosphorylation |
| NDUFB11 | Oxidative phosphorylation |
| NDUFB2 | Oxidative phosphorylation |
| NDUFB3 | Oxidative phosphorylation |
| NDUFB4 | Oxidative phosphorylation |
| NDUFB5 | Oxidative phosphorylation |
| NDUFB6 | Oxidative phosphorylation |
| NDUFB7 | Oxidative phosphorylation |
| NDUFB8 | Oxidative phosphorylation |
| NDUFB9 | Oxidative phosphorylation |
| NDUFC1 | Oxidative phosphorylation |
| NDUFC2 | Oxidative phosphorylation |
| NDUFS1 | Oxidative phosphorylation |
| NDUFS2 | Oxidative phosphorylation |
| NDUFS3 | Oxidative phosphorylation |
| NDUFS4 | Oxidative phosphorylation |
| NDUFS5 | Oxidative phosphorylation |
| NDUFS6 | Oxidative phosphorylation |
| NDUFS7 | Oxidative phosphorylation |
| NDUFS8 | Oxidative phosphorylation |
| NDUFV1 | Oxidative phosphorylation |
| NDUFV2 | Oxidative phosphorylation |
| NDUFV3 | Oxidative phosphorylation |
| NEU1 | Other glycan degradation; Sphingolipid metabolism |
| NEU2 | Other glycan degradation; Sphingolipid metabolism |
| NEU3 | Other glycan degradation; Sphingolipid metabolism |
| NEU4 | Other glycan degradation; Sphingolipid metabolism |
| NFS1 | Thiamine metabolism |
| NIT2 | Alanine, aspartate and glutamate metabolism |
| NME1 | Drug metabolism-other enzymes; Purine metabolism; Pyrimidine metabolism |
| NME1-NME2 | Purine metabolism；Pyrimidine metabolism |
| NME2 | Drug metabolism-other enzymes; Purine metabolism; Pyrimidine metabolism |
| NME3 | Drug metabolism-other enzymes; Purine metabolism; Pyrimidine metabolism |
| NME4 | Drug metabolism-other enzymes; Purine metabolism; Pyrimidine metabolism |
| NME5 | Purine metabolism；Pyrimidine metabolism |
| NME6 | Drug metabolism-other enzymes; Purine metabolism; Pyrimidine metabolism |
| NME7 | Drug metabolism-other enzymes; Purine metabolism; Pyrimidine metabolism |
| NMNAT1 | Nicotinate and nicotinamide metabolism |
| NMNAT2 | Nicotinate and nicotinamide metabolism |
| NMNAT3 | Nicotinate and nicotinamide metabolism |
| NMRK1 | Nicotinate and nicotinamide metabolism |
| NNMT | Nicotinate and nicotinamide metabolism |
| NNT | Nicotinate and nicotinamide metabolism |
| NOS1 | Arginine and proline metabolism; Arginine biosynthesis |
| NOS2 | Arginine and proline metabolism; Arginine biosynthesis |
| NOS3 | Arginine and proline metabolism; Arginine biosynthesis |
| NPL | Amino sugar and nucleotide sugar metabolism |
| NPR1 | Purine metabolism |
| NPR2 | Purine metabolism |
| NQO1 | Ubiquinone and other terpenoid-quinone biosynthesis; Ubiquinone and other terpenoid-quinone biosynthesis |
| NSD1 | Lysine degradation |
| NSD2 | Lysine degradation |
| NSD3 | Lysine degradation |
| NT5C | Nicotinate and nicotinamide metabolism; Purine metabolism; Pyrimidine metabolism |
| NT5C1A | Nicotinate and nicotinamide metabolism; Purine metabolism; Pyrimidine metabolism |
| NT5C1B | Nicotinate and nicotinamide metabolism; Purine metabolism; Pyrimidine metabolism |
| NT5C2 | Nicotinate and nicotinamide metabolism; Purine metabolism; Pyrimidine metabolism |
| NT5C3 | Nicotinate and nicotinamide metabolism; Purine metabolism; Pyrimidine metabolism |
| NT5C3A | Nicotinate and nicotinamide metabolism；Purine metabolism；Pyrimidine metabolism |
| NT5C3L | Nicotinate and nicotinamide metabolism; Purine metabolism; Pyrimidine metabolism |
| NT5E | Nicotinate and nicotinamide metabolism; Purine metabolism; Pyrimidine metabolism |
| NT5M | Nicotinate and nicotinamide metabolism; Purine metabolism; Pyrimidine metabolism |
| NUDT12 | Nicotinate and nicotinamide metabolism |
| NUDT16 | Purine metabolism |
| NUDT2 | Purine metabolism; Pyrimidine metabolism |
| NUDT5 | Purine metabolism |
| NUDT9 | Purine metabolism |
| NUS1 | Terpenoid backbone biosynthesis |
| OAT | Arginine and proline metabolism |
| OCRL | Inositol phosphate metabolism |
| ODC1 | Arginine and proline metabolism; Glutathione metabolism |
| OGDH | Lysine degradation; TCA cycle; Tryptophan metabolism |
| OGDHL | Lysine degradation; TCA cycle; Tryptophan metabolism |
| OGT | Other types of O-glycan biosynthesis |
| OLAH | Fatty acid biosynthesis |
| OPLAH | Glutathione metabolism |
| OSTC | Various types of N-glycan biosynthesis |
| OTC | Arginine biosynthesis |
| OXCT1 | Butanoate metabolism; Valine, leucine and isoleucine degradation |
| OXCT2 | Butanoate metabolism; Valine, leucine and isoleucine degradation |
| OXSM | Biotin metabolism; Fatty acid biosynthesis |
| P4HA1 | Arginine and proline metabolism |
| P4HA2 | Arginine and proline metabolism |
| P4HA3 | Arginine and proline metabolism |
| PAFAH1B1 | Ether lipid metabolism |
| PAFAH1B2 | Ether lipid metabolism |
| PAFAH1B3 | Ether lipid metabolism |
| PAFAH2 | Ether lipid metabolism |
| PAH | Folate biosynthesis; Phenylalanine metabolism; Phenylalanine, tyrosine and tryptophan biosynthesis |
| PAICS | Purine metabolism |
| PANK1 | Gluconeogenesis; Glycolysis; Gluconeogenesis; Pantothenate and CoA biosynthesis |
| PANK2 | Pantothenate and CoA biosynthesis |
| PANK3 | Pantothenate and CoA biosynthesis |
| PANK4 | Pantothenate and CoA biosynthesis |
| PAPSS1 | Purine metabolism; Selenocompound metabolism; Sulfur metabolism |
| PAPSS2 | Purine metabolism; Selenocompound metabolism; Sulfur metabolism |
| PC | Gluconeogenesis; Pyruvate metabolism; TCA cycle |
| PCBD1 | Folate biosynthesis |
| PCBD2 | Folate biosynthesis |
| PCCA | Glyoxylate and dicarboxylate metabolism; Propanoate metabolism; Valine, leucine and isoleucine degradation |
| PCCB | Glyoxylate and dicarboxylate metabolism; Propanoate metabolism; Valine, leucine and isoleucine degradation |
| PCK1 | Glycolysis; Gluconeogenesis; Pyruvate metabolism; TCA cycle |
| PCK2 | Glycolysis; Gluconeogenesis; Pyruvate metabolism; TCA cycle |
| PCYOX1 | Terpenoid backbone biosynthesis |
| PCYT1A | Glycerophospholipid metabolism; Phosphonate and phosphinate metabolism |
| PCYT1B | Glycerophospholipid metabolism; Phosphonate and phosphinate metabolism |
| PCYT2 | Glycerophospholipid metabolism; Phosphonate and phosphinate metabolism |
| PDE10A | Purine metabolism |
| PDE11A | Purine metabolism |
| PDE1A | Purine metabolism |
| PDE1B | Purine metabolism |
| PDE1C | Purine metabolism |
| PDE2A | Purine metabolism |
| PDE3A | Purine metabolism |
| PDE3B | Purine metabolism |
| PDE4A | Purine metabolism |
| PDE4B | Purine metabolism |
| PDE4C | Purine metabolism |
| PDE4D | Purine metabolism |
| PDE5A | Purine metabolism |
| PDE6A | Purine metabolism |
| PDE6B | Purine metabolism |
| PDE6C | Purine metabolism |
| PDE6D | Purine metabolism |
| PDE6G | Purine metabolism |
| PDE6H | Purine metabolism |
| PDE7A | Purine metabolism |
| PDE7B | Purine metabolism |
| PDE8A | Purine metabolism |
| PDE8B | Purine metabolism |
| PDE9A | Purine metabolism |
| PDHA1 | Glycolysis; Gluconeogenesis; Pyruvate metabolism; TCA cycle |
| PDHA2 | Glycolysis; Gluconeogenesis; Pyruvate metabolism; TCA cycle |
| PDHB | Glycolysis; Gluconeogenesis; Pyruvate metabolism; TCA cycle |
| PDSS1 | Terpenoid backbone biosynthesis |
| PDSS2 | Terpenoid backbone biosynthesis |
| PDXK | Vitamin B6 metabolism |
| PDXP | Vitamin B6 metabolism |
| PECI | Fatty acid degradation |
| PECR | Biosynthesis of unsaturated fatty acids |
| PEMT | Glycerophospholipid metabolism |
| PFAS | Purine metabolism |
| PFKFB1 | Fructose and mannose metabolism; Gluconeogenesis; Glycolysis |
| PFKFB2 | Fructose and mannose metabolism; Glycolysis |
| PFKFB3 | Fructose and mannose metabolism; Glycolysis |
| PFKFB4 | Fructose and mannose metabolism; Glycolysis |
| PFKL | Fructose and mannose metabolism; Galactose metabolism; Glycolysis; Gluconeogenesis; Pentose phosphate pathway |
| PFKM | Fructose and mannose metabolism; Galactose metabolism; Glycolysis; Gluconeogenesis; Pentose phosphate pathway |
| PFKP | Fructose and mannose metabolism; Galactose metabolism; Glycolysis; Gluconeogenesis; Pentose phosphate pathway |
| PGAM1 | Glycine, serine and threonine metabolism; Glycolysis; Gluconeogenesis |
| PGAM2 | Glycine, serine and threonine metabolism; Glycolysis; Gluconeogenesis |
| PGAM4 | Glycine, serine and threonine metabolism; Glycolysis; Gluconeogenesis |
| PGAP1 | Glycosylphosphatidylinositol (GPI)-anchor biosynthesis |
| PGD | Glutathione metabolism; Pentose phosphate pathway |
| PGK1 | Glycolysis; Gluconeogenesis |
| PGK2 | Glycolysis; Gluconeogenesis |
| PGLS | Pentose phosphate pathway |
| PGM1 | Amino sugar and nucleotide sugar metabolism; Galactose metabolism; Glycolysis; Gluconeogenesis; Pentose phosphate pathway; Purine metabolism; Starch and sucrose metabolism |
| PGM2 | Amino sugar and nucleotide sugar metabolism; Galactose metabolism; Glycolysis; Gluconeogenesis; Pentose phosphate pathway; Purine metabolism; Starch and sucrose metabolism |
| PGM2L1 | Starch and sucrose metabolism |
| PGM3 | Amino sugar and nucleotide sugar metabolism |
| PGP | Glyoxylate and dicarboxylate metabolism |
| PGS1 | Glycerophospholipid metabolism |
| PHGDH | Glycine, serine and threonine metabolism |
| PHOSPHO1 | Glycerophospholipid metabolism |
| PHOSPHO2 | Vitamin B6 metabolism |
| PHPT1 | Fructose and mannose metabolism；Amino sugar and nucleotide sugar metabolism |
| PI4K2A | Inositol phosphate metabolism |
| PI4K2B | Inositol phosphate metabolism |
| PI4KA | Inositol phosphate metabolism |
| PI4KB | Inositol phosphate metabolism |
| PIGA | Glycosylphosphatidylinositol (GPI)-anchor biosynthesis |
| PIGB | Glycosylphosphatidylinositol (GPI)-anchor biosynthesis |
| PIGC | Glycosylphosphatidylinositol (GPI)-anchor biosynthesis |
| PIGF | Glycosylphosphatidylinositol (GPI)-anchor biosynthesis |
| PIGG | Glycosylphosphatidylinositol (GPI)-anchor biosynthesis |
| PIGH | Glycosylphosphatidylinositol (GPI)-anchor biosynthesis |
| PIGK | Glycosylphosphatidylinositol (GPI)-anchor biosynthesis |
| PIGL | Glycosylphosphatidylinositol (GPI)-anchor biosynthesis |
| PIGM | Glycosylphosphatidylinositol (GPI)-anchor biosynthesis |
| PIGN | Glycosylphosphatidylinositol (GPI)-anchor biosynthesis |
| PIGO | Glycosylphosphatidylinositol (GPI)-anchor biosynthesis |
| PIGP | Glycosylphosphatidylinositol (GPI)-anchor biosynthesis |
| PIGQ | Glycosylphosphatidylinositol (GPI)-anchor biosynthesis |
| PIGS | Glycosylphosphatidylinositol (GPI)-anchor biosynthesis |
| PIGT | Glycosylphosphatidylinositol (GPI)-anchor biosynthesis |
| PIGU | Glycosylphosphatidylinositol (GPI)-anchor biosynthesis |
| PIGV | Glycosylphosphatidylinositol (GPI)-anchor biosynthesis |
| PIGW | Glycosylphosphatidylinositol (GPI)-anchor biosynthesis |
| PIGX | Glycosylphosphatidylinositol (GPI)-anchor biosynthesis |
| PIGY | Glycosylphosphatidylinositol (GPI)-anchor biosynthesis |
| PIGZ | Glycosylphosphatidylinositol (GPI)-anchor biosynthesis |
| PIK3C2A | Inositol phosphate metabolism |
| PIK3C2B | Inositol phosphate metabolism |
| PIK3C2G | Inositol phosphate metabolism |
| PIK3C3 | Inositol phosphate metabolism |
| PIK3CA | Inositol phosphate metabolism |
| PIK3CB | Inositol phosphate metabolism |
| PIK3CD | Inositol phosphate metabolism |
| PIK3CG | Inositol phosphate metabolism |
| PIKFYVE | Inositol phosphate metabolism |
| PIP4K2A | Inositol phosphate metabolism |
| PIP4K2B | Inositol phosphate metabolism |
| PIP4K2C | Inositol phosphate metabolism |
| PIP5K1A | Inositol phosphate metabolism |
| PIP5K1B | Inositol phosphate metabolism |
| PIP5K1C | Inositol phosphate metabolism |
| PIP5K2A | Inositol phosphate metabolism |
| PIP5K2B | Inositol phosphate metabolism |
| PIP5KL1 | Inositol phosphate metabolism |
| PIPOX | Glycine, serine and threonine metabolism; Lysine degradation |
| PISD | Glycerophospholipid metabolism |
| PKLR | Glycolysis; Gluconeogenesis; Purine metabolism; Pyruvate metabolism |
| PKM | Glycolysis; Purine metabolism；Pyruvate metabolism |
| PKM2 | Glycolysis; Purine metabolism; Pyruvate metabolism |
| PLA2G10 | alpha-Linolenic acid metabolism; Arachidonic acid metabolism; Ether lipid metabolism; Glycerophospholipid metabolism; Linoleic acid metabolism |
| PLA2G12A | alpha-Linolenic acid metabolism; Arachidonic acid metabolism; Ether lipid metabolism; Glycerophospholipid metabolism; Linoleic acid metabolism |
| PLA2G12B | alpha-Linolenic acid metabolism; Arachidonic acid metabolism; Ether lipid metabolism; Glycerophospholipid metabolism; Linoleic acid metabolism |
| PLA2G15 | Glycerophospholipid metabolism |
| PLA2G16 | alpha-Linolenic acid metabolism; Arachidonic acid metabolism; Ether lipid metabolism; Glycerophospholipid metabolism; Linoleic acid metabolism |
| PLA2G1B | alpha-Linolenic acid metabolism; Arachidonic acid metabolism; Ether lipid metabolism; Glycerophospholipid metabolism; Linoleic acid metabolism |
| PLA2G2A | alpha-Linolenic acid metabolism; Arachidonic acid metabolism; Ether lipid metabolism; Glycerophospholipid metabolism; Linoleic acid metabolism |
| PLA2G2C | alpha-Linolenic acid metabolism; Arachidonic acid metabolism; Ether lipid metabolism; Glycerophospholipid metabolism; Linoleic acid metabolism |
| PLA2G2D | alpha-Linolenic acid metabolism; Arachidonic acid metabolism; Ether lipid metabolism; Glycerophospholipid metabolism; Linoleic acid metabolism |
| PLA2G2E | alpha-Linolenic acid metabolism; Arachidonic acid metabolism; Ether lipid metabolism; Glycerophospholipid metabolism; Linoleic acid metabolism |
| PLA2G2F | alpha-Linolenic acid metabolism; Arachidonic acid metabolism; Ether lipid metabolism; Glycerophospholipid metabolism; Linoleic acid metabolism |
| PLA2G3 | alpha-Linolenic acid metabolism; Arachidonic acid metabolism; Ether lipid metabolism; Glycerophospholipid metabolism; Linoleic acid metabolism |
| PLA2G4A | alpha-Linolenic acid metabolism; Arachidonic acid metabolism; Ether lipid metabolism; Glycerophospholipid metabolism; Linoleic acid metabolism |
| PLA2G4B | alpha-Linolenic acid metabolism; Arachidonic acid metabolism; Ether lipid metabolism; Glycerophospholipid metabolism; Linoleic acid metabolism |
| PLA2G4C | alpha-Linolenic acid metabolism; Arachidonic acid metabolism; Ether lipid metabolism; Glycerophospholipid metabolism; Linoleic acid metabolism |
| PLA2G4D | alpha-Linolenic acid metabolism; Arachidonic acid metabolism; Ether lipid metabolism; Glycerophospholipid metabolism; Linoleic acid metabolism |
| PLA2G4E | alpha-Linolenic acid metabolism; Arachidonic acid metabolism; Ether lipid metabolism; Glycerophospholipid metabolism; Linoleic acid metabolism |
| PLA2G4F | alpha-Linolenic acid metabolism; Arachidonic acid metabolism; Ether lipid metabolism; Glycerophospholipid metabolism; Linoleic acid metabolism |
| PLA2G5 | alpha-Linolenic acid metabolism; Arachidonic acid metabolism; Ether lipid metabolism; Glycerophospholipid metabolism; Linoleic acid metabolism |
| PLA2G6 | alpha-Linolenic acid metabolism; Arachidonic acid metabolism; Ether lipid metabolism; Glycerophospholipid metabolism; Linoleic acid metabolism |
| PLA2G7 | Ether lipid metabolism |
| PLB1 | alpha-Linolenic acid metabolism; Arachidonic acid metabolism; Ether lipid metabolism; Glycerophospholipid metabolism; Linoleic acid metabolism |
| PLCB1 | Inositol phosphate metabolism |
| PLCB2 | Inositol phosphate metabolism |
| PLCB3 | Inositol phosphate metabolism |
| PLCB4 | Inositol phosphate metabolism |
| PLCD1 | Inositol phosphate metabolism |
| PLCD3 | Inositol phosphate metabolism |
| PLCD4 | Inositol phosphate metabolism |
| PLCE1 | Inositol phosphate metabolism |
| PLCG1 | Inositol phosphate metabolism |
| PLCG2 | Inositol phosphate metabolism |
| PLCH1 | Inositol phosphate metabolism |
| PLCH2 | Inositol phosphate metabolism |
| PLCZ1 | Inositol phosphate metabolism |
| PLD1 | Ether lipid metabolism; Glycerophospholipid metabolism |
| PLD2 | Ether lipid metabolism; Glycerophospholipid metabolism |
| PLD3 | Ether lipid metabolism; Glycerophospholipid metabolism |
| PLD4 | Ether lipid metabolism; Glycerophospholipid metabolism |
| PLOD1 | Lysine degradation |
| PLOD2 | Lysine degradation |
| PLOD3 | Lysine degradation; Other types of O-glycan biosynthesis |
| PLPP1 | Ether lipid metabolism；Glycerolipid metabolism；Glycerophospholipid metabolism；Sphingolipid metabolism |
| PLPP2 | Ether lipid metabolism；Glycerolipid metabolism；Glycerophospholipid metabolism；Sphingolipid metabolism |
| PLPP3 | Ether lipid metabolism；Glycerolipid metabolism；Glycerophospholipid metabolism；Sphingolipid metabolism |
| PMM1 | Amino sugar and nucleotide sugar metabolism; Fructose and mannose metabolism |
| PMM2 | Amino sugar and nucleotide sugar metabolism; Fructose and mannose metabolism |
| PMVK | Terpenoid backbone biosynthesis |
| PNLIP | Glycerolipid metabolism; Glycerolipid metabolism |
| PNLIPRP1 | Glycerolipid metabolism; Glycerolipid metabolism |
| PNLIPRP2 | Glycerolipid metabolism; Glycerolipid metabolism |
| PNLIPRP3 | Glycerolipid metabolism; Glycerolipid metabolism |
| PNMT | Tyrosine metabolism |
| PNP | Nicotinate and nicotinamide metabolism; Purine metabolism; Pyrimidine metabolism |
| PNPLA2 | Glycerolipid metabolism; Glycerolipid metabolism |
| PNPLA3 | Glycerolipid metabolism; Glycerolipid metabolism |
| PNPLA4 | Retinol metabolism |
| PNPLA6 | Glycerophospholipid metabolism |
| PNPLA7 | Glycerophospholipid metabolism |
| PNPO | Vitamin B6 metabolism |
| PNPT1 | Purine metabolism; Pyrimidine metabolism |
| POFUT1 | Other types of O-glycan biosynthesis |
| POFUT2 | Other types of O-glycan biosynthesis |
| POLA1 | Purine metabolism; Pyrimidine metabolism |
| POLA2 | Purine metabolism; Pyrimidine metabolism |
| POLD1 | Purine metabolism; Pyrimidine metabolism |
| POLD2 | Purine metabolism; Pyrimidine metabolism |
| POLD3 | Purine metabolism; Pyrimidine metabolism |
| POLD4 | Purine metabolism; Pyrimidine metabolism |
| POLE | Purine metabolism; Pyrimidine metabolism |
| POLE2 | Purine metabolism; Pyrimidine metabolism |
| POLE3 | Purine metabolism; Pyrimidine metabolism |
| POLE4 | Purine metabolism; Pyrimidine metabolism |
| POLR1A | Purine metabolism; Pyrimidine metabolism |
| POLR1B | Purine metabolism; Pyrimidine metabolism |
| POLR1C | Purine metabolism; Pyrimidine metabolism |
| POLR1D | Purine metabolism; Pyrimidine metabolism |
| POLR1E | Purine metabolism; Pyrimidine metabolism |
| POLR1H | Purine metabolism；Pyrimidine metabolism |
| POLR2A | Purine metabolism; Pyrimidine metabolism |
| POLR2B | Purine metabolism; Pyrimidine metabolism |
| POLR2C | Purine metabolism; Pyrimidine metabolism |
| POLR2D | Purine metabolism; Pyrimidine metabolism |
| POLR2E | Purine metabolism; Pyrimidine metabolism |
| POLR2F | Purine metabolism; Pyrimidine metabolism |
| POLR2G | Purine metabolism; Pyrimidine metabolism |
| POLR2H | Purine metabolism; Pyrimidine metabolism |
| POLR2I | Purine metabolism; Pyrimidine metabolism |
| POLR2J | Purine metabolism; Pyrimidine metabolism |
| POLR2J2 | Purine metabolism; Pyrimidine metabolism |
| POLR2J3 | Purine metabolism; Pyrimidine metabolism |
| POLR2K | Purine metabolism; Pyrimidine metabolism |
| POLR2L | Purine metabolism; Pyrimidine metabolism |
| POLR3A | Purine metabolism; Pyrimidine metabolism |
| POLR3B | Purine metabolism; Pyrimidine metabolism |
| POLR3C | Purine metabolism; Pyrimidine metabolism |
| POLR3D | Purine metabolism; Pyrimidine metabolism |
| POLR3E | Purine metabolism; Pyrimidine metabolism |
| POLR3F | Purine metabolism; Pyrimidine metabolism |
| POLR3G | Purine metabolism; Pyrimidine metabolism |
| POLR3GL | Purine metabolism; Pyrimidine metabolism |
| POLR3H | Purine metabolism; Pyrimidine metabolism |
| POLR3K | Purine metabolism; Pyrimidine metabolism |
| POMGNT1 | Mannose type O-glycan biosynthesis |
| POMT1 | Mannose type O-glycan biosynthesis; Other types of O-glycan biosynthesis |
| POMT2 | Mannose type O-glycan biosynthesis; Other types of O-glycan biosynthesis |
| PPA1 | Oxidative phosphorylation |
| PPA2 | Oxidative phosphorylation |
| PPAP2A | Ether lipid metabolism; Glycerolipid metabolism; Glycerolipid metabolism; Glycerophospholipid metabolism; Sphingolipid metabolism |
| PPAP2B | Ether lipid metabolism; Glycerolipid metabolism; Glycerolipid metabolism; Glycerophospholipid metabolism; Sphingolipid metabolism |
| PPAP2C | Ether lipid metabolism; Glycerolipid metabolism; Glycerolipid metabolism; Glycerophospholipid metabolism; Sphingolipid metabolism |
| PPAPDC1A | Glycerolipid metabolism; Glycerolipid metabolism; Glycerophospholipid metabolism |
| PPAPDC1B | Glycerolipid metabolism; Glycerolipid metabolism; Glycerophospholipid metabolism |
| PPAT | Alanine, aspartate and glutamate metabolism; Purine metabolism |
| PPCDC | Pantothenate and CoA biosynthesis |
| PPCS | Pantothenate and CoA biosynthesis |
| PPOX | Porphyrin and chlorophyll metabolism |
| PPT1 | Fatty acid elongation |
| PPT2 | Fatty acid elongation |
| PRDM2 | Lysine degradation |
| PRDM6 | Lysine degradation |
| PRDM7 | Lysine degradation |
| PRDM9 | Lysine degradation |
| PRDX6 | Phenylalanine metabolism |
| PRHOXNB | Purine metabolism |
| PRIM1 | Purine metabolism; Pyrimidine metabolism |
| PRIM2 | Purine metabolism; Pyrimidine metabolism |
| PRODH | Arginine and proline metabolism |
| PRODH2 | Arginine and proline metabolism |
| PRPS1 | Pentose phosphate pathway; Purine metabolism |
| PRPS1L1 | Pentose phosphate pathway; Purine metabolism |
| PRPS2 | Pentose phosphate pathway; Purine metabolism |
| PRUNE | Purine metabolism |
| PRUNE1 | Purine metabolism |
| PSAT1 | Glycine, serine and threonine metabolism; Vitamin B6 metabolism |
| PSPH | Glycine, serine and threonine metabolism |
| PSTK | Selenocompound metabolism |
| PTDSS1 | Glycerophospholipid metabolism |
| PTDSS2 | Glycerophospholipid metabolism |
| PTEN | Inositol phosphate metabolism |
| PTGDS | Arachidonic acid metabolism |
| PTGES | Arachidonic acid metabolism |
| PTGES2 | Arachidonic acid metabolism |
| PTGES3 | Arachidonic acid metabolism |
| PTGIS | Arachidonic acid metabolism |
| PTGS1 | Arachidonic acid metabolism |
| PTGS2 | Arachidonic acid metabolism |
| PTPLA | Biosynthesis of unsaturated fatty acids; Fatty acid elongation |
| PTPLAD1 | Biosynthesis of unsaturated fatty acids; Fatty acid elongation |
| PTPLAD2 | Biosynthesis of unsaturated fatty acids; Fatty acid elongation |
| PTPLB | Biosynthesis of unsaturated fatty acids; Fatty acid elongation |
| PTS | Folate biosynthesis |
| PYCR1 | Arginine and proline metabolism |
| PYCR2 | Arginine and proline metabolism |
| PYCR3 | Arginine and proline metabolism |
| PYCRL | Arginine and proline metabolism |
| PYGB | Starch and sucrose metabolism |
| PYGL | Starch and sucrose metabolism |
| PYGM | Starch and sucrose metabolism |
| QDPR | Folate biosynthesis |
| QPRT | Nicotinate and nicotinamide metabolism |
| RBKS | Pentose phosphate pathway |
| RCE1 | Terpenoid backbone biosynthesis |
| RDH10 | Retinol metabolism |
| RDH11 | Retinol metabolism |
| RDH12 | Retinol metabolism |
| RDH16 | Retinol metabolism |
| RDH5 | Retinol metabolism |
| RDH8 | Retinol metabolism |
| RENBP | Amino sugar and nucleotide sugar metabolism |
| RETSAT | Retinol metabolism |
| RFK | Riboflavin metabolism |
| RFNG | Other types of O-glycan biosynthesis |
| RFT1 | N-Glycan biosynthesis |
| RGN | Ascorbate and aldarate metabolism; Pentose phosphate pathway |
| RIMKLA | Alanine, aspartate and glutamate metabolism |
| RIMKLB | Alanine, aspartate and glutamate metabolism |
| RPE | Pentose and glucuronate interconversions; Pentose phosphate pathway |
| RPE65 | Retinol metabolism |
| RPEL1 | Pentose and glucuronate interconversions; Pentose phosphate pathway |
| RPIA | Pentose phosphate pathway |
| RPN1 | Various types of N-glycan biosynthesis |
| RPN2 | Various types of N-glycan biosynthesis |
| RRM1 | Drug metabolism-other enzymes; Glutathione metabolism; Purine metabolism; Pyrimidine metabolism |
| RRM2 | Drug metabolism-other enzymes; Glutathione metabolism; Purine metabolism; Pyrimidine metabolism |
| RRM2B | Drug metabolism-other enzymes; Glutathione metabolism; Purine metabolism; Pyrimidine metabolism |
| SACM1L | Inositol phosphate metabolism |
| SARDH | Glycine, serine and threonine metabolism |
| SAT1 | Arginine and proline metabolism |
| SAT2 | Arginine and proline metabolism |
| SCD | Biosynthesis of unsaturated fatty acids |
| SCD5 | Biosynthesis of unsaturated fatty acids |
| SCLY | Selenocompound metabolism |
| SDHA | Oxidative phosphorylation; TCA cycle |
| SDHB | Oxidative phosphorylation; TCA cycle |
| SDHC | Oxidative phosphorylation; TCA cycle |
| SDHD | Oxidative phosphorylation; TCA cycle |
| SDR16C5 | Retinol metabolism |
| SDS | Cysteine and methionine metabolism; Glycine, serine and threonine metabolism; Valine, leucine and isoleucine biosynthesis |
| SDSL | Cysteine and methionine metabolism; Glycine, serine and threonine metabolism; Valine, leucine and isoleucine biosynthesis |
| SEPHS1 | Selenocompound metabolism |
| SEPHS2 | Selenocompound metabolism |
| SEPSECS | Selenocompound metabolism |
| SETD1A | Lysine degradation |
| SETD1B | Lysine degradation |
| SETD2 | Lysine degradation |
| SETD3 | Lysine degradation |
| SETD7 | Lysine degradation |
| SETD8 | Lysine degradation |
| SETDB1 | Lysine degradation |
| SETDB2 | Lysine degradation |
| SETMAR | Lysine degradation |
| SGK196 | Mannose type O-glycan biosynthesis |
| SGMS1 | Sphingolipid metabolism |
| SGMS2 | Sphingolipid metabolism |
| SGPL1 | Sphingolipid metabolism |
| SGPP1 | Sphingolipid metabolism |
| SGPP2 | Sphingolipid metabolism |
| SGSH | Glycosaminoglycan degradation |
| SHMT1 | Glycine, serine and threonine metabolism; Glyoxylate and dicarboxylate metabolism; One carbon pool by folate |
| SHMT2 | Glycine, serine and threonine metabolism; Glyoxylate and dicarboxylate metabolism; One carbon pool by folate |
| SI | Galactose metabolism; Starch and sucrose metabolism |
| SLC25A11 | Gluconeogenesis |
| SLC2A2 | Glycolysis; Gluconeogenesis |
| SLC33A1 | Glycosphingolipid biosynthesis-ganglio series |
| SLC37A4 | Gluconeogenesis |
| SMOX | Arginine and proline metabolism; beta-Alanine metabolism |
| SMPD1 | Sphingolipid metabolism |
| SMPD2 | Sphingolipid metabolism |
| SMPD3 | Sphingolipid metabolism |
| SMPD4 | Sphingolipid metabolism |
| SMS | Arginine and proline metabolism; beta-Alanine metabolism; Cysteine and methionine metabolism; Glutathione metabolism |
| SORD | Fructose and mannose metabolism; Pentose and glucuronate interconversions |
| SPAM1 | Glycosaminoglycan degradation |
| SPHK1 | Sphingolipid metabolism |
| SPHK2 | Sphingolipid metabolism |
| SPR | Folate biosynthesis |
| SPTLC1 | Sphingolipid metabolism |
| SPTLC2 | Sphingolipid metabolism |
| SPTLC3 | Sphingolipid metabolism |
| SQRDL | Sulfur metabolism |
| SRM | Arginine and proline metabolism; beta-Alanine metabolism; Cysteine and methionine metabolism; Glutathione metabolism |
| SRR | Glycine, serine and threonine metabolism; D-Amino acid metabolism |
| ST3GAL1 | Glycosaminoglycan biosynthesis-keratan sulfate; Glycosphingolipid biosynthesis-ganglio series; Glycosphingolipid biosynthesis-globo and isoglobo series; Mucin type O-glycan biosynthesis |
| ST3GAL2 | Glycosaminoglycan biosynthesis-keratan sulfate; Glycosphingolipid biosynthesis-ganglio series; Glycosphingolipid biosynthesis-globo and isoglobo series; Mucin type O-glycan biosynthesis |
| ST3GAL3 | Glycosaminoglycan biosynthesis-keratan sulfate; Glycosphingolipid biosynthesis-lacto and neolacto series; Mannose type O-glycan biosynthesis; Other types of O-glycan biosynthesis; Various types of N-glycan biosynthesis |
| ST3GAL4 | Glycosphingolipid biosynthesis-lacto and neolacto series |
| ST3GAL5 | Glycosphingolipid biosynthesis-ganglio series |
| ST3GAL6 | Glycosphingolipid biosynthesis-lacto and neolacto series |
| ST6GAL1 | N-Glycan biosynthesis; Other types of O-glycan biosynthesis |
| ST6GAL2 | N-Glycan biosynthesis; Other types of O-glycan biosynthesis |
| ST6GALNAC1 | Mucin type O-glycan biosynthesis |
| ST6GALNAC3 | Glycosphingolipid biosynthesis-ganglio series |
| ST6GALNAC4 | Glycosphingolipid biosynthesis-ganglio series |
| ST6GALNAC5 | Glycosphingolipid biosynthesis-ganglio series |
| ST6GALNAC6 | Glycosphingolipid biosynthesis-ganglio series |
| ST8SIA1 | Glycosphingolipid biosynthesis-ganglio series; Glycosphingolipid biosynthesis-globo and isoglobo series; Glycosphingolipid biosynthesis-lacto and neolacto series |
| ST8SIA5 | Glycosphingolipid biosynthesis-ganglio series |
| STT3A | Various types of N-glycan biosynthesis |
| STT3B | Various types of N-glycan biosynthesis |
| SUCLA2 | Propanoate metabolism; TCA cycle |
| SUCLG1 | Propanoate metabolism; TCA cycle |
| SUCLG2 | Propanoate metabolism; TCA cycle |
| SUCLG2P2 | Citrate cycle tca cycle；Propanoate metabolism |
| SULT1A1 | Sulfur metabolism |
| SULT1A2 | Sulfur metabolism |
| SULT1A3 | Sulfur metabolism |
| SULT1A4 | Sulfur metabolism |
| SULT2A1 | Metabolism of xenobiotics by cytochrome P450 |
| SUOX | Sulfur metabolism |
| SUV39H1 | Lysine degradation |
| SUV39H2 | Lysine degradation |
| SUV420H1 | Lysine degradation |
| SUV420H2 | Lysine degradation |
| SYNJ1 | Inositol phosphate metabolism |
| SYNJ2 | Inositol phosphate metabolism |
| TAFAZZIN | Glycerophospholipid metabolism |
| TALDO1 | Pentose phosphate pathway |
| TAT | Cysteine and methionine metabolism; Phenylalanine metabolism; Phenylalanine, tyrosine and tryptophan biosynthesis; Tyrosine metabolism; Ubiquinone and other terpenoid-quinone biosynthesis; Ubiquinone and other terpenoid-quinone biosynthesis |
| TAZ | Glycerophospholipid metabolism |
| TBXAS1 | Arachidonic acid metabolism |
| TCIRG1 | Oxidative phosphorylation |
| TDO2 | Tryptophan metabolism |
| TECR | Biosynthesis of unsaturated fatty acids; Fatty acid elongation |
| TH | Folate biosynthesis; Tyrosine metabolism |
| THEM4 | Fatty acid elongation |
| THEM5 | Fatty acid elongation |
| THTPA | Thiamine metabolism |
| TK1 | Drug metabolism-other enzymes; Pyrimidine metabolism |
| TK2 | Drug metabolism-other enzymes; Pyrimidine metabolism |
| TKFC | Glycerolipid metabolism |
| TKT | Pentose phosphate pathway |
| TKTL1 | Pentose phosphate pathway |
| TKTL2 | Pentose phosphate pathway |
| TMEM258 | Various types of N-glycan biosynthesis |
| TMEM5 | Mannose type O-glycan biosynthesis |
| TMEM86B | Ether lipid metabolism |
| TMLHE | Lysine degradation |
| TPH1 | Folate biosynthesis; Tryptophan metabolism |
| TPH2 | Folate biosynthesis; Tryptophan metabolism |
| TPI1 | Fructose and mannose metabolism; Glycolysis; Gluconeogenesis; Inositol phosphate metabolism |
| TPK1 | Thiamine metabolism |
| TPMT | Drug metabolism-other enzymes |
| TPO | Tyrosine metabolism |
| TRDMT1 | Cysteine and methionine metabolism |
| TREH | Starch and sucrose metabolism |
| TRMT11 | Histidine metabolism；Selenoamino acid metabolism；Tyrosine metabolism |
| TST | Cysteine and methionine metabolism; Sulfur metabolism |
| TSTA3 | Amino sugar and nucleotide sugar metabolism; Fructose and mannose metabolism |
| TUSC3 | Various types of N-glycan biosynthesis |
| TWISTNB | Purine metabolism; Pyrimidine metabolism |
| TXNDC12 | Glutathione metabolism |
| TXNRD1 | Selenocompound metabolism |
| TXNRD2 | Selenocompound metabolism |
| TXNRD3 | Selenocompound metabolism |
| TYMP | Drug metabolism-other enzymes; Pyrimidine metabolism |
| TYMS | One carbon pool by folate; Pyrimidine metabolism |
| TYR | Tyrosine metabolism |
| TYRP1 | Tyrosine metabolism |
| UAP1 | Amino sugar and nucleotide sugar metabolism |
| UAP1L1 | Amino sugar and nucleotide sugar metabolism |
| UCK1 | Drug metabolism-other enzymes; Pyrimidine metabolism |
| UCK2 | Drug metabolism-other enzymes; Pyrimidine metabolism |
| UCKL1 | Drug metabolism-other enzymes; Pyrimidine metabolism |
| UGCG | Sphingolipid metabolism |
| UGDH | Amino sugar and nucleotide sugar metabolism; Ascorbate and aldarate metabolism; Pentose and glucuronate interconversions |
| UGP2 | Amino sugar and nucleotide sugar metabolism; Galactose metabolism; Pentose and glucuronate interconversions; Starch and sucrose metabolism |
| UGT1A1 | Ascorbate and aldarate metabolism; Drug metabolism-cytochrome P450; Drug metabolism-other enzymes; Metabolism of xenobiotics by cytochrome P450; Pentose and glucuronate interconversions; Porphyrin and chlorophyll metabolism; Retinol metabolism |
| UGT1A10 | Ascorbate and aldarate metabolism; Drug metabolism-cytochrome P450; Drug metabolism-other enzymes; Metabolism of xenobiotics by cytochrome P450; Pentose and glucuronate interconversions; Porphyrin and chlorophyll metabolism; Retinol metabolism |
| UGT1A3 | Ascorbate and aldarate metabolism; Drug metabolism-cytochrome P450; Drug metabolism-other enzymes; Metabolism of xenobiotics by cytochrome P450; Pentose and glucuronate interconversions; Porphyrin and chlorophyll metabolism; Retinol metabolism |
| UGT1A4 | Ascorbate and aldarate metabolism; Drug metabolism-cytochrome P450; Drug metabolism-other enzymes; Metabolism of xenobiotics by cytochrome P450; Pentose and glucuronate interconversions; Porphyrin and chlorophyll metabolism; Retinol metabolism |
| UGT1A5 | Ascorbate and aldarate metabolism; Drug metabolism-cytochrome P450; Drug metabolism-other enzymes; Metabolism of xenobiotics by cytochrome P450; Pentose and glucuronate interconversions; Porphyrin and chlorophyll metabolism; Retinol metabolism |
| UGT1A6 | Ascorbate and aldarate metabolism; Drug metabolism-cytochrome P450; Drug metabolism-other enzymes; Metabolism of xenobiotics by cytochrome P450; Pentose and glucuronate interconversions; Porphyrin and chlorophyll metabolism; Retinol metabolism |
| UGT1A7 | Ascorbate and aldarate metabolism; Drug metabolism-cytochrome P450; Drug metabolism-other enzymes; Metabolism of xenobiotics by cytochrome P450; Pentose and glucuronate interconversions; Porphyrin and chlorophyll metabolism; Retinol metabolism |
| UGT1A8 | Ascorbate and aldarate metabolism; Drug metabolism-cytochrome P450; Drug metabolism-other enzymes; Metabolism of xenobiotics by cytochrome P450; Pentose and glucuronate interconversions; Porphyrin and chlorophyll metabolism; Retinol metabolism |
| UGT1A9 | Ascorbate and aldarate metabolism; Drug metabolism-cytochrome P450; Drug metabolism-other enzymes; Metabolism of xenobiotics by cytochrome P450; Pentose and glucuronate interconversions; Porphyrin and chlorophyll metabolism; Retinol metabolism |
| UGT2A1 | Ascorbate and aldarate metabolism; Drug metabolism-cytochrome P450; Drug metabolism-other enzymes; Metabolism of xenobiotics by cytochrome P450; Pentose and glucuronate interconversions; Porphyrin and chlorophyll metabolism; Retinol metabolism |
| UGT2A2 | Ascorbate and aldarate metabolism; Drug metabolism-cytochrome P450; Drug metabolism-other enzymes; Metabolism of xenobiotics by cytochrome P450; Pentose and glucuronate interconversions; Porphyrin and chlorophyll metabolism; Retinol metabolism |
| UGT2A3 | Ascorbate and aldarate metabolism; Drug metabolism-cytochrome P450; Drug metabolism-other enzymes; Metabolism of xenobiotics by cytochrome P450; Pentose and glucuronate interconversions; Porphyrin and chlorophyll metabolism; Retinol metabolism |
| UGT2B10 | Ascorbate and aldarate metabolism; Drug metabolism-cytochrome P450; Drug metabolism-other enzymes; Metabolism of xenobiotics by cytochrome P450; Pentose and glucuronate interconversions; Porphyrin and chlorophyll metabolism; Retinol metabolism |
| UGT2B11 | Ascorbate and aldarate metabolism; Drug metabolism-cytochrome P450; Drug metabolism-other enzymes; Metabolism of xenobiotics by cytochrome P450; Pentose and glucuronate interconversions; Porphyrin and chlorophyll metabolism; Retinol metabolism |
| UGT2B15 | Ascorbate and aldarate metabolism; Drug metabolism-cytochrome P450; Drug metabolism-other enzymes; Metabolism of xenobiotics by cytochrome P450; Pentose and glucuronate interconversions; Porphyrin and chlorophyll metabolism; Retinol metabolism |
| UGT2B17 | Ascorbate and aldarate metabolism; Drug metabolism-cytochrome P450; Drug metabolism-other enzymes; Metabolism of xenobiotics by cytochrome P450; Pentose and glucuronate interconversions; Porphyrin and chlorophyll metabolism; Retinol metabolism |
| UGT2B28 | Ascorbate and aldarate metabolism; Drug metabolism-cytochrome P450; Drug metabolism-other enzymes; Metabolism of xenobiotics by cytochrome P450; Pentose and glucuronate interconversions; Porphyrin and chlorophyll metabolism; Retinol metabolism |
| UGT2B4 | Ascorbate and aldarate metabolism; Drug metabolism-cytochrome P450; Drug metabolism-other enzymes; Metabolism of xenobiotics by cytochrome P450; Pentose and glucuronate interconversions; Porphyrin and chlorophyll metabolism; Retinol metabolism |
| UGT2B7 | Ascorbate and aldarate metabolism; Drug metabolism-cytochrome P450; Drug metabolism-other enzymes; Metabolism of xenobiotics by cytochrome P450; Pentose and glucuronate interconversions; Porphyrin and chlorophyll metabolism; Retinol metabolism |
| UGT8 | Ether lipid metabolism; Sphingolipid metabolism |
| UMPS | Drug metabolism-other enzymes; Pyrimidine metabolism |
| UPB1 | beta-Alanine metabolism; Drug metabolism-other enzymes; Pantothenate and CoA biosynthesis; Pyrimidine metabolism |
| UPP1 | Drug metabolism-other enzymes; Pyrimidine metabolism |
| UPP2 | Drug metabolism-other enzymes; Pyrimidine metabolism |
| UPRT | Pyrimidine metabolism |
| UQCR10 | Oxidative phosphorylation |
| UQCR10P1 | Oxidative phosphorylation |
| UQCR11 | Oxidative phosphorylation |
| UQCRB | Oxidative phosphorylation |
| UQCRC1 | Oxidative phosphorylation |
| UQCRC2 | Oxidative phosphorylation |
| UQCRFS1 | Oxidative phosphorylation |
| UQCRH | Oxidative phosphorylation |
| UQCRHL | Oxidative phosphorylation |
| UQCRQ | Oxidative phosphorylation |
| URAD | Purine metabolism |
| UROC1 | Histidine metabolism |
| UROD | Porphyrin and chlorophyll metabolism |
| UROS | Porphyrin and chlorophyll metabolism |
| UST | Glycosaminoglycan biosynthesis-chondroitin sulfate / dermatan sulfate |
| UXS1 | Amino sugar and nucleotide sugar metabolism |
| VKORC1 | Ubiquinone and other terpenoid-quinone biosynthesis; Ubiquinone and other terpenoid-quinone biosynthesis |
| VKORC1L1 | Ubiquinone and other terpenoid-quinone biosynthesis; Ubiquinone and other terpenoid-quinone biosynthesis |
| VNN1 | Pantothenate and CoA biosynthesis |
| VNN2 | Pantothenate and CoA biosynthesis |
| VNN3 | Pantothenate and CoA biosynthesis |
| WBSCR16 | Mucin type O-glycan biosynthesis |
| WBSCR17 | Mucin type O-glycan biosynthesis |
| WHSC1 | Lysine degradation |
| WHSC1L1 | Lysine degradation |
| XDH | Drug metabolism-other enzymes; Purine metabolism |
| XYLB | Pentose and glucuronate interconversions |
| XYLT1 | Glycosaminoglycan biosynthesis-chondroitin sulfate / dermatan sulfate; Glycosaminoglycan biosynthesis-heparan sulfate / heparin |
| XYLT2 | Glycosaminoglycan biosynthesis-chondroitin sulfate / dermatan sulfate; Glycosaminoglycan biosynthesis-heparan sulfate / heparin |
| YOD1 | Biosynthesis of unsaturated fatty acids；Limonene and pinene degradation |
| ZMPSTE24 | Terpenoid backbone biosynthesis |
| ZNRD1 | Purine metabolism; Pyrimidine metabolism |
