# Supplementary material for: Metabolic-pathway-based classification of tendinopathy reveals a precise therapeutic strategy targeting chondro-modulatory tenocytes
Source: Bone Res. 2026 Aug 27;14:91. doi: 10.1038/s41413-026-00574-w (PMC13522621; doi:10.1038/s41413-026-00574-w)
Supplement: Supplementary file 4 [file 41413_2026_574_MOESM21_ESM.docx]

Table S3. Related to Figure 1. Reactome human metabolic pathway gene list.

| Gene symbol | Metabolic pathways involved |
| --- | --- |
| AACS | Ketone body metabolism; Synthesis of Ketone Bodies |
| AADAT | Lysine catabolism;Tryptophan catabolism |
| AASDHPPT | Metabolism of water-soluble vitamins and cofactors;Vitamin B5 pantothenate metabolism |
| AASS | Lysine catabolism |
| ABCC1 | Fatty acid metabolism; Metabolism of water-soluble vitamins and cofactors; Cobalamin (Cbl, vitamin B12) transport and metabolism |
| ABCC5 | Glycosaminoglycan metabolism |
| ABCD1 | Fatty acid metabolism; Peroxisomal lipid metabolism; β-oxidation of very long chain fatty acids |
| ABCD4 | Metabolism of water-soluble vitamins and cofactors; Cobalamin (Cbl, vitamin B12) transport and metabolism |
| ABHD10 | Glucuronidation |
| ABHD14B | Cytosolic sulfonation of small molecules |
| ABHD3 | Phospholipid metabolism; Glycerophospholipid biosynthesis |
| ABHD4 | Phospholipid metabolism; Glycerophospholipid biosynthesis |
| ABHD5 | Triglyceride metabolism; Triglyceride catabolism |
| ACAA1 | Fatty acid metabolism; Peroxisomal lipid metabolism;β-oxidation of very long chain fatty acids |
| ACAA2 | Fatty acid metabolism;Mitochondrial fatty acid β-oxidation |
| ACACA | Fatty acid metabolism;Fatty acyl-CoA biosynthesis;Carnitine metabolism;Metabolism of water-soluble vitamins and cofactors |
| ACACB | Fatty acid metabolism;Carnitine metabolism;Metabolism of water-soluble vitamins and cofactors |
| ACAD10 | Fatty acid metabolism; Mitochondrial fatty acid β-oxidation |
| ACAD11 | Fatty acid metabolism; Mitochondrial fatty acid β-oxidation |
| ACAD8 | Branched-chain amino acid catabolism |
| ACADL | Fatty acid metabolism;Mitochondrial fatty acid β-oxidation;Mitochondrial fatty acid β-oxidation of saturated fatty acids;Mitochondrial fatty acid β-oxidation of unsaturated fatty acids;β-oxidation of lauroyl CoA to decanoyl-CoA-CoA |
| ACADM | Fatty acid metabolism;Mitochondrial fatty acid β-oxidation;Mitochondrial fatty acid β-oxidation of saturated fatty acids;Mitochondrial fatty acid β-oxidation of unsaturated fatty acids;β-oxidation of decanoyl-CoA to octanoyl-CoA-CoA;β-oxidation of octanoyl-CoA to hexanoyl-CoA |
| ACADS | Fatty acid metabolism;Mitochondrial fatty acid β-oxidation;Mitochondrial fatty acid β-oxidation of saturated fatty acids;β-oxidation of butanoyl-CoA to acetyl-CoA;β-oxidation of hexanoyl-CoA to Butanoyl-CoA |
| ACADSB | Branched-chain amino acid catabolism |
| ACADVL | Fatty acid metabolism; Mitochondrial fatty acid β-oxidation; Mitochondrial fatty acid β-oxidation of saturated fatty acids |
| ACAN | Glycosaminoglycan metabolism |
| ACAT1 | Branched-chain amino acid catabolism; Ketone body metabolism; Synthesis of Ketone Bodies; Utilization of Ketone Bodies |
| ACBD4 | Fatty acid metabolism; Peroxisomal lipid metabolism |
| ACBD5 | Fatty acid metabolism; Peroxisomal lipid metabolism |
| ACBD6 | Fatty acid metabolism; Mitochondrial fatty acid β-oxidation |
| ACBD7 | Fatty acid metabolism; Mitochondrial fatty acid β-oxidation |
| ACHE | Phospholipid metabolism; Glycerophospholipid biosynthesis |
| ACLY | Fatty acid metabolism; Fatty acyl-CoA biosynthesis |
| ACMSD | Tryptophan catabolism |
| ACOT1 | Fatty acid metabolism; Mitochondrial fatty acid β-oxidation |
| ACOT11 | Fatty acid metabolism; Mitochondrial fatty acid β-oxidation |
| ACOT12 | Fatty acid metabolism; Mitochondrial fatty acid β-oxidation |
| ACOT13 | Fatty acid metabolism; Mitochondrial fatty acid β-oxidation |
| ACOT2 | Fatty acid metabolism; Mitochondrial fatty acid β-oxidation |
| ACOT4 | Fatty acid metabolism; Peroxisomal lipid metabolism; β-oxidation of very long chain fatty acids |
| ACOT6 | Fatty acid metabolism; Peroxisomal lipid metabolism; β-oxidation of very long chain fatty acids |
| ACOT7 | Fatty acid metabolism; Mitochondrial fatty acid β-oxidation |
| ACOT8 | Fatty acid metabolism; Peroxisomal lipid metabolism; β-oxidation of pristanoyl-CoA; β-oxidation of very long chain fatty acids |
| ACOT9 | Fatty acid metabolism; Mitochondrial fatty acid β-oxidation |
| ACOX1 | Fatty acid metabolism; Peroxisomal lipid metabolism; β-oxidation of very long chain fatty acids |
| ACOX2 | Fatty acid metabolism; Peroxisomal lipid metabolism; β-oxidation of pristanoyl-CoA |
| ACOX3 | Fatty acid metabolism; Peroxisomal lipid metabolism; β-oxidation of pristanoyl-CoA |
| ACOXL | Fatty acid metabolism; Peroxisomal lipid metabolism; β-oxidation of pristanoyl-CoA |
| ACP5 | Metabolism of water-soluble vitamins and cofactors |
| ACP6 | Phospholipid metabolism; Glycerophospholipid biosynthesis |
| ACSBG1 | Fatty acid metabolism; Fatty acyl-CoA biosynthesis |
| ACSBG2 | Fatty acid metabolism; Fatty acyl-CoA biosynthesis |
| ACSF2 | Fatty acid metabolism; Mitochondrial fatty acid β-oxidation |
| ACSF3 | Fatty acid metabolism; Fatty acyl-CoA biosynthesis |
| ACSL1 | Fatty acid metabolism; Fatty acyl-CoA biosynthesis |
| ACSL3 | Fatty acid metabolism; Fatty acyl-CoA biosynthesis |
| ACSL4 | Fatty acid metabolism; Fatty acyl-CoA biosynthesis |
| ACSL5 | Fatty acid metabolism; Fatty acyl-CoA biosynthesis |
| ACSL6 | Fatty acid metabolism; Fatty acyl-CoA biosynthesis |
| ACSM1 | Amino Acid conjugation |
| ACSM2A | Amino Acid conjugation |
| ACSM2B | Amino Acid conjugation |
| ACSM3 | Fatty acid metabolism; Mitochondrial fatty acid β-oxidation; Mitochondrial fatty acid β-oxidation of saturated fatty acids; β-oxidation of butanoyl-CoA to acetyl-CoA |
| ACSM4 | Amino Acid conjugation |
| ACSM5 | Amino Acid conjugation |
| ACSM6 | Fatty acid metabolism; Mitochondrial fatty acid β-oxidation; Mitochondrial fatty acid β-oxidation of saturated fatty acids; β-oxidation of butanoyl-CoA to acetyl-CoA |
| ACSS1 | Ethanol oxidation |
| ACSS2 | Ethanol oxidation |
| ACSS3 | Ketone body metabolism; Synthesis of Ketone Bodies |
| ADA | Purine salvage |
| ADAL | Purine salvage |
| ADH1A | Ethanol oxidation |
| ADH1B | Ethanol oxidation |
| ADH1C | Ethanol oxidation |
| ADH4 | Ethanol oxidation |
| ADH5 | Ethanol oxidation |
| ADH6 | Ethanol oxidation |
| ADH7 | Ethanol oxidation |
| ADI1 | Sulfur amino acid metabolism |
| ADK | Purine salvage |
| ADO | Sulfur amino acid metabolism |
| ADPRM | Purine catabolism |
| ADSL | Purine ribonucleoside monophosphate biosynthesis |
| ADSS1 | Purine ribonucleoside monophosphate biosynthesis |
| ADSS2 | Purine ribonucleoside monophosphate biosynthesis |
| AFMID | Tryptophan catabolism |
| AGK | Phospholipid metabolism; Glycerophospholipid biosynthesis |
| AGMAT | Metabolism of polyamines |
| AGMO | Triglyceride metabolism; Triglyceride biosynthesis |
| AGPAT1 | Phospholipid metabolism; Glycerophospholipid biosynthesis |
| AGPAT2 | Phospholipid metabolism; Glycerophospholipid biosynthesis |
| AGPAT3 | Phospholipid metabolism; Glycerophospholipid biosynthesis |
| AGPAT4 | Phospholipid metabolism; Glycerophospholipid biosynthesis |
| AGPAT5 | Phospholipid metabolism; Glycerophospholipid biosynthesis |
| AGRN | Glycosaminoglycan metabolism; Metabolism of fat-soluble vitamins |
| AGXT | Glyoxylate metabolism and glycine degradation |
| AGXT2 | Glyoxylate metabolism and glycine degradation; Pyrimidine catabolism |
| AHCY | Sulfur amino acid metabolism; Selenoamino acid metabolism; Methylation |
| AIMP1 | Selenoamino acid metabolism |
| AIMP2 | Selenoamino acid metabolism |
| AKR1A1 | Formation of xylulose-5-phosphate; Glutathione conjugation |
| AKR1B10 | Metabolism of fat-soluble vitamins |
| AKR1C1 | Metabolism of fat-soluble vitamins |
| AKR1C3 | Fatty acid metabolism; Metabolism of fat-soluble vitamins |
| AKR1C4 | Metabolism of fat-soluble vitamins |
| AKT1 | Metabolism of cofactors; Tetrahydrobiopterin (BH4) synthesis, recycling, salvage and regulation |
| ALDH1A1 | Ethanol oxidation |
| ALDH1B1 | Ethanol oxidation |
| ALDH1L1 | Metabolism of water-soluble vitamins and cofactors |
| ALDH1L2 | Metabolism of water-soluble vitamins and cofactors |
| ALDH2 | Ethanol oxidation |
| ALDH3A2 | Fatty acid metabolism; Peroxisomal lipid metabolism |
| ALDH4A1 | Proline catabolism; Glyoxylate metabolism and glycine degradation |
| ALDH6A1 | Branched-chain amino acid catabolism |
| ALDH7A1 | Lysine catabolism; Choline catabolism |
| ALDH9A1 | Carnitine synthesis |
| ALOX12 | Fatty acid metabolism |
| ALOX12B | Fatty acid metabolism |
| ALOX15 | Fatty acid metabolism |
| ALOX15B | Fatty acid metabolism |
| ALOX5 | Fatty acid metabolism |
| ALOX5AP | Fatty acid metabolism |
| ALOXE3 | Fatty acid metabolism |
| ALPI | Phospholipid metabolism; Glycerophospholipid biosynthesis |
| AMACR | Fatty acid metabolism; Peroxisomal lipid metabolism; β-oxidation of pristanoyl-CoA |
| AMD1 | Metabolism of polyamines |
| AMDHD1 | Histidine catabolism |
| AMN | Metabolism of water-soluble vitamins and cofactors; Cobalamin (Cbl, vitamin B12) transport and metabolism |
| AMPD1 | Purine salvage |
| AMPD2 | Purine salvage |
| AMPD3 | Purine salvage |
| AMT | Glyoxylate metabolism and glycine degradation |
| AOX1 | Metabolism of water-soluble vitamins and cofactors |
| APIP | Sulfur amino acid metabolism |
| APOA1 | Metabolism of fat-soluble vitamins |
| APOA2 | Metabolism of fat-soluble vitamins |
| APOA4 | Metabolism of fat-soluble vitamins |
| APOB | Metabolism of fat-soluble vitamins |
| APOC2 | Metabolism of fat-soluble vitamins |
| APOC3 | Metabolism of fat-soluble vitamins |
| APOE | Metabolism of fat-soluble vitamins |
| APOM | Metabolism of fat-soluble vitamins |
| APRT | Purine salvage |
| ARF1 | Phospholipid metabolism |
| ARF3 | Phospholipid metabolism |
| ARG1 | Urea cycle |
| ARG2 | Urea cycle |
| ARSB | Glycosaminoglycan metabolism |
| AS3MT | Methylation |
| ASL | Urea cycle |
| ASS1 | Urea cycle |
| ATIC | Purine ribonucleoside monophosphate biosynthesis |
| AUH | Branched-chain amino acid catabolism |
| AWAT1 | Fatty acid metabolism |
| AWAT2 | Phospholipid metabolism; Glycerophospholipid biosynthesis |
| AZIN1 | Metabolism of polyamines |
| AZIN2 | Metabolism of polyamines |
| B3GALT6 | Glycosaminoglycan metabolism |
| B3GAT1 | Glycosaminoglycan metabolism |
| B3GAT2 | Glycosaminoglycan metabolism |
| B3GAT3 | Glycosaminoglycan metabolism |
| B3GNT2 | Glycosaminoglycan metabolism |
| B3GNT3 | Glycosaminoglycan metabolism |
| B3GNT4 | Glycosaminoglycan metabolism |
| B3GNT7 | Glycosaminoglycan metabolism |
| B4GALT1 | Lactose synthesis; Glycosaminoglycan metabolism |
| B4GALT2 | Glycosaminoglycan metabolism |
| B4GALT3 | Glycosaminoglycan metabolism |
| B4GALT4 | Glycosaminoglycan metabolism |
| B4GALT5 | Glycosaminoglycan metabolism |
| B4GALT6 | Glycosaminoglycan metabolism |
| B4GALT7 | Glycosaminoglycan metabolism |
| B4GAT1 | Glycosaminoglycan metabolism |
| BBOX1 | Carnitine synthesis |
| BCAN | Glycosaminoglycan metabolism |
| BCAT1 | Branched-chain amino acid catabolism |
| BCAT2 | Branched-chain amino acid catabolism |
| BCHE | Phospholipid metabolism; Glycerophospholipid biosynthesis |
| BCKDHA | Branched-chain amino acid catabolism; Glyoxylate metabolism and glycine degradation |
| BCKDHB | Branched-chain amino acid catabolism; Glyoxylate metabolism and glycine degradation |
| BCKDK | Branched-chain amino acid catabolism |
| BCO1 | Metabolism of fat-soluble vitamins |
| BCO2 | Metabolism of fat-soluble vitamins |
| BDH1 | Ketone body metabolism; Synthesis of Ketone Bodies; Utilization of Ketone Bodies |
| BDH2 | Ketone body metabolism; Synthesis of Ketone Bodies |
| BGN | Glycosaminoglycan metabolism |
| BHMT | Sulfur amino acid metabolism; Choline catabolism |
| BHMT2 | Sulfur amino acid metabolism |
| BMX | Phospholipid metabolism |
| BPNT1 | Cytosolic sulfonation of small molecules |
| BPNT2 | Cytosolic sulfonation of small molecules |
| BST1 | Metabolism of water-soluble vitamins and cofactors |
| BTD | Metabolism of water-soluble vitamins and cofactors |
| CAD | Pyrimidine biosynthesis |
| CALM1 | Metabolism of cofactors; Tetrahydrobiopterin (BH4) synthesis, recycling, salvage and regulation |
| CALM2 | Metabolism of cofactors; Tetrahydrobiopterin (BH4) synthesis, recycling, salvage and regulation |
| CALM3 | Metabolism of cofactors; Tetrahydrobiopterin (BH4) synthesis, recycling, salvage and regulation |
| CARNMT1 | Histidine catabolism |
| CARNS1 | Histidine catabolism |
| CAV1 | Triglyceride metabolism; Triglyceride catabolism |
| CBLIF | Metabolism of water-soluble vitamins and cofactors; Cobalamin (Cbl, vitamin B12) transport and metabolism |
| CBR1 | Fatty acid metabolism |
| CBR4 | Fatty acid metabolism; Fatty acyl-CoA biosynthesis |
| CBS | Sulfur amino acid metabolism; Selenoamino acid metabolism |
| CD320 | Metabolism of water-soluble vitamins and cofactors; Cobalamin (Cbl, vitamin B12) transport and metabolism |
| CD38 | Metabolism of water-soluble vitamins and cofactors |
| CD44 | Glycosaminoglycan metabolism |
| CDA | Pyrimidine salvage |
| CDIPT | Phospholipid metabolism; Glycerophospholipid biosynthesis |
| CDO1 | Sulfur amino acid metabolism |
| CDS1 | Phospholipid metabolism; Glycerophospholipid biosynthesis |
| CDS2 | Phospholipid metabolism; Glycerophospholipid biosynthesis |
| CEMIP | Glycosaminoglycan metabolism |
| CEPT1 | Phospholipid metabolism; Glycerophospholipid biosynthesis |
| CHAC1 | Glutathione conjugation |
| CHAC2 | Glutathione conjugation |
| CHAT | Phospholipid metabolism; Glycerophospholipid biosynthesis |
| CHDH | Choline catabolism |
| CHKA | Phospholipid metabolism; Glycerophospholipid biosynthesis |
| CHKB | Phospholipid metabolism; Glycerophospholipid biosynthesis |
| CHP1 | Glycosaminoglycan metabolism |
| CHPF | Glycosaminoglycan metabolism |
| CHPF2 | Glycosaminoglycan metabolism |
| CHPT1 | Phospholipid metabolism; Glycerophospholipid biosynthesis |
| CHST1 | Glycosaminoglycan metabolism |
| CHST11 | Glycosaminoglycan metabolism |
| CHST12 | Glycosaminoglycan metabolism |
| CHST13 | Glycosaminoglycan metabolism |
| CHST14 | Glycosaminoglycan metabolism |
| CHST15 | Glycosaminoglycan metabolism |
| CHST2 | Glycosaminoglycan metabolism |
| CHST3 | Glycosaminoglycan metabolism |
| CHST5 | Glycosaminoglycan metabolism |
| CHST6 | Glycosaminoglycan metabolism |
| CHST7 | Glycosaminoglycan metabolism |
| CHST9 | Glycosaminoglycan metabolism |
| CHSY1 | Glycosaminoglycan metabolism |
| CHSY3 | Glycosaminoglycan metabolism |
| CKB | Creatine metabolism |
| CKM | Creatine metabolism |
| CKMT1A | Creatine metabolism |
| CKMT1B | Creatine metabolism |
| CKMT2 | Creatine metabolism |
| CLPS | Metabolism of fat-soluble vitamins |
| CNDP2 | Glutathione conjugation |
| COASY | Metabolism of water-soluble vitamins and cofactors; Vitamin B5 pantothenate metabolism |
| COMT | Methylation |
| COQ3 | Metabolism of cofactors; Ubiquinol biosynthesis |
| COQ5 | Metabolism of cofactors; Ubiquinol biosynthesis |
| COQ6 | Metabolism of cofactors; Ubiquinol biosynthesis |
| COQ7 | Metabolism of cofactors; Ubiquinol biosynthesis |
| COQ9 | Metabolism of cofactors; Ubiquinol biosynthesis |
| CPNE1 | Phospholipid metabolism; Glycerophospholipid biosynthesis |
| CPNE3 | Phospholipid metabolism; Glycerophospholipid biosynthesis |
| CPNE6 | Phospholipid metabolism; Glycerophospholipid biosynthesis |
| CPNE7 | Phospholipid metabolism; Glycerophospholipid biosynthesis |
| CPS1 | Urea cycle |
| CPT1A | Fatty acid metabolism; Carnitine metabolism |
| CPT1B | Fatty acid metabolism; Carnitine metabolism |
| CPT2 | Fatty acid metabolism; Carnitine metabolism |
| CRAT | Fatty acid metabolism; Peroxisomal lipid metabolism; β-oxidation of pristanoyl-CoA |
| CRLS1 | Phospholipid metabolism; Glycerophospholipid biosynthesis |
| CROT | Fatty acid metabolism; Peroxisomal lipid metabolism; β-oxidation of pristanoyl-CoA |
| CRYL1 | Formation of xylulose-5-phosphate |
| CRYM | Lysine catabolism |
| CSAD | Sulfur amino acid metabolism |
| CSGALNACT1 | Glycosaminoglycan metabolism |
| CSGALNACT2 | Glycosaminoglycan metabolism |
| CSNK2A1 | Phospholipid metabolism; Glycerophospholipid biosynthesis |
| CSNK2A2 | Phospholipid metabolism; Glycerophospholipid biosynthesis |
| CSNK2B | Phospholipid metabolism; Glycerophospholipid biosynthesis |
| CSPG4 | Glycosaminoglycan metabolism |
| CSPG5 | Glycosaminoglycan metabolism |
| CTH | Sulfur amino acid metabolism; Selenoamino acid metabolism |
| CTRB1 | Metabolism of water-soluble vitamins and cofactors; Cobalamin (Cbl, vitamin B12) transport and metabolism |
| CTRB2 | Metabolism of water-soluble vitamins and cofactors; Cobalamin (Cbl, vitamin B12) transport and metabolism |
| CUBN | Metabolism of water-soluble vitamins and cofactors; Cobalamin (Cbl, vitamin B12) transport and metabolism |
| CYB5A | Metabolism of water-soluble vitamins and cofactors; Vitamin C ascorbate metabolism |
| CYB5R3 | Metabolism of water-soluble vitamins and cofactors; Vitamin C ascorbate metabolism |
| CYP1A1 | Fatty acid metabolism |
| CYP1A2 | Fatty acid metabolism |
| CYP1B1 | Fatty acid metabolism |
| CYP2C19 | Fatty acid metabolism |
| CYP2C8 | Fatty acid metabolism |
| CYP2C9 | Fatty acid metabolism |
| CYP2J2 | Fatty acid metabolism |
| CYP2U1 | Fatty acid metabolism |
| CYP4A11 | Fatty acid metabolism |
| CYP4A22 | Fatty acid metabolism |
| CYP4B1 | Fatty acid metabolism |
| CYP4F11 | Fatty acid metabolism |
| CYP4F2 | Fatty acid metabolism |
| CYP4F22 | Fatty acid metabolism |
| CYP4F3 | Fatty acid metabolism |
| CYP4F8 | Fatty acid metabolism |
| CYP8B1 | Fatty acid metabolism; Metabolism of water-soluble vitamins and cofactors |
| DAO | Glyoxylate metabolism and glycine degradation |
| DARS1 | Selenoamino acid metabolism |
| DBI | Fatty acid metabolism; Mitochondrial fatty acid β-oxidation |
| DBT | Branched-chain amino acid catabolism; Glyoxylate metabolism and glycine degradation |
| DCK | Purine salvage; Pyrimidine salvage |
| DCN | Glycosaminoglycan metabolism |
| DCXR | Formation of xylulose-5-phosphate |
| DDHD1 | Phospholipid metabolism; Glycerophospholipid biosynthesis |
| DDHD2 | Phospholipid metabolism; Glycerophospholipid biosynthesis |
| DDO | Glyoxylate metabolism and glycine degradation |
| DECR1 | Fatty acid metabolism; Mitochondrial fatty acid β-oxidation; Mitochondrial fatty acid β-oxidation |
| DECR2 | Fatty acid metabolism; Peroxisomal lipid metabolism; β-oxidation of very long chain fatty acids |
| DGAT1 | Triglyceride metabolism; Triglyceride biosynthesis; Phospholipid metabolism; Glycerophospholipid biosynthesis |
| DGAT2 | Triglyceride metabolism; Triglyceride biosynthesis; Phospholipid metabolism; Glycerophospholipid biosynthesis |
| DGAT2L6 | Phospholipid metabolism; Glycerophospholipid biosynthesis |
| DGUOK | Purine salvage |
| DHFR | Metabolism of water-soluble vitamins and cofactors; Metabolism of cofactors; Tetrahydrobiopterin (BH4) synthesis, recycling, salvage and regulation |
| DHFR2 | Metabolism of water-soluble vitamins and cofactors |
| DHODH | Pyrimidine biosynthesis |
| DHTKD1 | Glyoxylate metabolism and glycine degradation |
| DLAT | Glyoxylate metabolism and glycine degradation |
| DLD | Branched-chain amino acid catabolism; Lysine catabolism; Glyoxylate metabolism and glycine degradation |
| DLST | Lysine catabolism; Glyoxylate metabolism and glycine degradation |
| DMGDH | Choline catabolism |
| DNPH1 | Purine catabolism |
| DPEP1 | Fatty acid metabolism |
| DPEP2 | Fatty acid metabolism |
| DPEP3 | Fatty acid metabolism |
| DPYD | Pyrimidine catabolism |
| DPYS | Pyrimidine catabolism |
| DSE | Glycosaminoglycan metabolism |
| DSEL | Glycosaminoglycan metabolism |
| ECHS1 | Branched-chain amino acid catabolism; Fatty acid metabolism; Mitochondrial fatty acid β-oxidation; Mitochondrial fatty acid β-oxidation of saturated fatty acids; β-oxidation of butanoyl-CoA to acetyl-CoA; β-oxidation of decanoyl-CoA to octanoyl-CoA-CoA; β-oxidation of hexanoyl-CoA to Butanoyl-CoA; β-oxidation of lauroyl CoA to decanoyl-CoA-CoA; β-oxidation of octanoyl-CoA to hexanoyl-CoA |
| ECI1 | Fatty acid metabolism; Mitochondrial fatty acid β-oxidation; Mitochondrial fatty acid β-oxidation of unsaturated fatty acids |
| ECI2 | Fatty acid metabolism; Peroxisomal lipid metabolism; β-oxidation of very long chain fatty acids |
| EEF1E1 | Selenoamino acid metabolism |
| EEFSEC | Selenoamino acid metabolism |
| EHHADH | Fatty acid metabolism; Peroxisomal lipid metabolism; β-oxidation of very long chain fatty acids |
| ELOVL1 | Fatty acid metabolism; Fatty acyl-CoA biosynthesis |
| ELOVL2 | Fatty acid metabolism; Fatty acyl-CoA biosynthesis |
| ELOVL3 | Fatty acid metabolism; Fatty acyl-CoA biosynthesis |
| ELOVL4 | Fatty acid metabolism; Fatty acyl-CoA biosynthesis |
| ELOVL5 | Fatty acid metabolism; Fatty acyl-CoA biosynthesis |
| ELOVL6 | Fatty acid metabolism; Fatty acyl-CoA biosynthesis |
| ELOVL7 | Fatty acid metabolism; Fatty acyl-CoA biosynthesis |
| ENOPH1 | Sulfur amino acid metabolism |
| ENPP1 | Metabolism of water-soluble vitamins and cofactors; Vitamin B5 pantothenate metabolism |
| ENPP2 | Metabolism of water-soluble vitamins and cofactors; Vitamin B5 pantothenate metabolism |
| ENPP3 | Metabolism of water-soluble vitamins and cofactors; Vitamin B5 pantothenate metabolism |
| ENPP6 | Phospholipid metabolism; Glycerophospholipid catabolism |
| EPHX2 | Fatty acid metabolism |
| EPRS1 | Selenoamino acid metabolism |
| ESD | Glutathione conjugation |
| ETHE1 | Sulfur amino acid metabolism |
| ETNK1 | Phospholipid metabolism; Glycerophospholipid biosynthesis |
| ETNK2 | Phospholipid metabolism; Glycerophospholipid biosynthesis |
| ETNPPL | Phospholipid metabolism; Glycerophospholipid biosynthesis |
| EXT1 | Glycosaminoglycan metabolism |
| EXT2 | Glycosaminoglycan metabolism |
| FAAH | Fatty acid metabolism |
| FAAH2 | Fatty acid metabolism |
| FABP1 | Triglyceride metabolism; Triglyceride catabolism |
| FABP12 | Triglyceride metabolism; Triglyceride catabolism |
| FABP2 | Triglyceride metabolism; Triglyceride catabolism |
| FABP3 | Triglyceride metabolism; Triglyceride catabolism |
| FABP4 | Triglyceride metabolism; Triglyceride catabolism |
| FABP5 | Triglyceride metabolism; Triglyceride catabolism |
| FABP6 | Triglyceride metabolism; Triglyceride catabolism |
| FABP7 | Triglyceride metabolism; Triglyceride catabolism |
| FABP9 | Triglyceride metabolism; Triglyceride catabolism |
| FADS1 | Fatty acid metabolism |
| FADS2 | Fatty acid metabolism |
| FASN | Fatty acid metabolism; Fatty acyl-CoA biosynthesis; Metabolism of water-soluble vitamins and cofactors; Vitamin B5 pantothenate metabolism |
| FIG4 | Phospholipid metabolism |
| FLAD1 | Metabolism of water-soluble vitamins and cofactors |
| FMO1 | FMO oxidises nucleophiles |
| FMO2 | FMO oxidises nucleophiles |
| FMO3 | FMO oxidises nucleophiles |
| FMOD | Glycosaminoglycan metabolism |
| FOLR2 | Metabolism of water-soluble vitamins and cofactors |
| FPGS | Metabolism of water-soluble vitamins and cofactors |
| FTCD | Histidine catabolism |
| GADL1 | Sulfur amino acid metabolism |
| GALNS | Glycosaminoglycan metabolism |
| GAMT | Creatine metabolism |
| GART | Purine ribonucleoside monophosphate biosynthesis |
| GATM | Creatine metabolism |
| GCAT | Threonine catabolism |
| GCDH | Lysine catabolism |
| GCH1 | Metabolism of cofactors; Tetrahydrobiopterin (BH4) synthesis, recycling, salvage and regulation |
| GCHFR | Metabolism of cofactors; Tetrahydrobiopterin (BH4) synthesis, recycling, salvage and regulation |
| GCLC | Glutathione conjugation |
| GCLM | Glutathione conjugation |
| GCSH | Glyoxylate metabolism and glycine degradation |
| GDA | Purine catabolism |
| GDE1 | Phospholipid metabolism; Glycerophospholipid catabolism |
| GDPD1 | Phospholipid metabolism; Glycerophospholipid catabolism |
| GDPD3 | Phospholipid metabolism; Glycerophospholipid catabolism |
| GDPD5 | Phospholipid metabolism; Glycerophospholipid catabolism |
| GGCT | Glutathione conjugation |
| GGT1 | Fatty acid metabolism; Glutathione conjugation |
| GGT5 | Fatty acid metabolism; Glutathione conjugation |
| GGT6 | Glutathione conjugation |
| GGT7 | Glutathione conjugation |
| GK | Triglyceride metabolism; Triglyceride biosynthesis |
| GK2 | Triglyceride metabolism; Triglyceride biosynthesis |
| GLB1 | Glycosaminoglycan metabolism |
| GLB1L | Glycosaminoglycan metabolism |
| GLCE | Glycosaminoglycan metabolism |
| GLDC | Glycosaminoglycan metabolism |
| GLYAT | Glyoxylate metabolism and glycine degradation |
| GLYATL1 | Amino Acid conjugation |
| GLYATL2 | Amino Acid conjugation |
| GLYATL3 | Amino Acid conjugation |
| GMPR | Purine salvage |
| GMPR2 | Purine salvage |
| GMPS | Purine ribonucleoside monophosphate biosynthesis |
| GNMT | Selenoamino acid metabolism; Glyoxylate metabolism and glycine degradation |
| GNPAT | Phospholipid metabolism; Glycerophospholipid biosynthesis |
| GNS | Glycosaminoglycan metabolism |
| GOT1 | Sulfur amino acid metabolism |
| GOT2 | Sulfur amino acid metabolism; Glyoxylate metabolism and glycine degradation |
| GPAM | Triglyceride metabolism; Triglyceride biosynthesis; Phospholipid metabolism; Glycerophospholipid biosynthesis |
| GPAT2 | Triglyceride metabolism; Triglyceride biosynthesis; Phospholipid metabolism; Glycerophospholipid biosynthesis |
| GPAT3 | Phospholipid metabolism; Glycerophospholipid biosynthesis |
| GPAT4 | Phospholipid metabolism; Glycerophospholipid biosynthesis |
| GPC1 | Glycosaminoglycan metabolism; Metabolism of fat-soluble vitamins |
| GPC2 | Glycosaminoglycan metabolism; Metabolism of fat-soluble vitamins |
| GPC3 | Glycosaminoglycan metabolism; Metabolism of fat-soluble vitamins |
| GPC4 | Glycosaminoglycan metabolism; Metabolism of fat-soluble vitamins |
| GPC5 | Glycosaminoglycan metabolism; Metabolism of fat-soluble vitamins |
| GPC6 | Glycosaminoglycan metabolism; Metabolism of fat-soluble vitamins |
| GPCPD1 | Phospholipid metabolism; Glycerophospholipid biosynthesis |
| GPD1 | Phospholipid metabolism; Glycerophospholipid biosynthesis |
| GPD1L | Phospholipid metabolism; Glycerophospholipid biosynthesis |
| GPD2 | Triglyceride metabolism; Triglyceride catabolism; Phospholipid metabolism; Glycerophospholipid biosynthesis |
| GPHN | Metabolism of water-soluble vitamins and cofactors |
| GPIHBP1 | Metabolism of fat-soluble vitamins |
| GPX1 | Fatty acid metabolism |
| GPX2 | Fatty acid metabolism |
| GPX4 | Fatty acid metabolism |
| GRHPR | Glyoxylate metabolism and glycine degradation |
| GSR | Selenoamino acid metabolism |
| GSS | Glutathione conjugation |
| GSTA1 | Glutathione conjugation |
| GSTA2 | Glutathione conjugation |
| GSTA3 | Glutathione conjugation |
| GSTA4 | Glutathione conjugation |
| GSTA5 | Glutathione conjugation |
| GSTK1 | Glutathione conjugation |
| GSTM1 | Glutathione conjugation |
| GSTM2 | Glutathione conjugation |
| GSTM3 | Glutathione conjugation |
| GSTM4 | Glutathione conjugation |
| GSTM5 | Glutathione conjugation |
| GSTO1 | Metabolism of water-soluble vitamins and cofactors; Vitamin C ascorbate metabolism; Methylation; Glutathione conjugation |
| GSTO2 | Metabolism of water-soluble vitamins and cofactors; Vitamin C ascorbate metabolism; Glutathione conjugation |
| GSTP1 | Glutathione conjugation |
| GSTT1 | Glutathione conjugation |
| GSTT2 | Glutathione conjugation |
| GSTT2B | Glutathione conjugation |
| GSTZ1 | Glutathione conjugation |
| GUSB | Glycosaminoglycan metabolism |
| HAAO | Tryptophan catabolism |
| HACD1 | Fatty acid metabolism; Fatty acyl-CoA biosynthesis |
| HACD2 | Fatty acid metabolism; Fatty acyl-CoA biosynthesis |
| HACD3 | Fatty acid metabolism; Fatty acyl-CoA biosynthesis |
| HACD4 | Fatty acid metabolism; Fatty acyl-CoA biosynthesis |
| HACL1 | Fatty acid metabolism; Peroxisomal lipid metabolism |
| HADH | Fatty acid metabolism; Mitochondrial fatty acid β-oxidation; Mitochondrial fatty acid β-oxidation of saturated fatty acids; β-oxidation of butanoyl-CoA to acetyl-CoA; β-oxidation of decanoyl-CoA to octanoyl-CoA-CoA; β-oxidation of hexanoyl-CoA to Butanoyl-CoA; β-oxidation of lauroyl CoA to decanoyl-CoA-CoA; β-oxidation of octanoyl-CoA to hexanoyl-CoA |
| HADHA | Fatty acid metabolism; Mitochondrial fatty acid β-oxidation; Mitochondrial fatty acid β-oxidation of saturated fatty acids; Mitochondrial fatty acid β-oxidation of unsaturated fatty acids; β-oxidation of decanoyl-CoA to octanoyl-CoA-CoA; β-oxidation of hexanoyl-CoA to Butanoyl-CoA; β-oxidation of lauroyl CoA to decanoyl-CoA-CoA; β-oxidation of octanoyl-CoA to hexanoyl-CoA; Phospholipid metabolism; Glycerophospholipid biosynthesis |
| HADHB | Fatty acid metabolism; Mitochondrial fatty acid β-oxidation; Mitochondrial fatty acid β-oxidation of saturated fatty acids; Mitochondrial fatty acid β-oxidation of unsaturated fatty acids; β-oxidation of decanoyl-CoA to octanoyl-CoA-CoA; β-oxidation of hexanoyl-CoA to Butanoyl-CoA; β-oxidation of lauroyl CoA to decanoyl-CoA-CoA; β-oxidation of octanoyl-CoA to hexanoyl-CoA; Phospholipid metabolism; Glycerophospholipid biosynthesis |
| HAL | Histidine catabolism |
| HAO1 | Glyoxylate metabolism and glycine degradation |
| HAO2 | Fatty acid metabolism;Peroxisomal lipid metabolism |
| HAS1 | Glycosaminoglycan metabolism |
| HAS2 | Glycosaminoglycan metabolism |
| HAS3 | Glycosaminoglycan metabolism |
| HDC | Histidine catabolism |
| HEXA | Glycosaminoglycan metabolism |
| HEXB | Glycosaminoglycan metabolism |
| HGSNAT | Glycosaminoglycan metabolism |
| HIBADH | Branched-chain amino acid catabolism |
| HIBCH | Branched-chain amino acid catabolism |
| HLCS | Metabolism of water-soluble vitamins and cofactors |
| HMGCL | Ketone body metabolism; Synthesis of Ketone Bodies |
| HMGCLL1 | Ketone body metabolism; Synthesis of Ketone Bodies |
| HMGCS2 | Ketone body metabolism; Synthesis of Ketone Bodies |
| HMMR | Glycosaminoglycan metabolism |
| HNMT | Histidine catabolism; Selenoamino acid metabolism |
| HOGA1 | Glyoxylate metabolism and glycine degradation |
| HPGD | Fatty acid metabolism |
| HPGDS | Fatty acid metabolism; Glutathione conjugation |
| HPRT1 | Purine salvage |
| HPSE | Glycosaminoglycan metabolism |
| HPSE2 | Glycosaminoglycan metabolism |
| HS2ST1 | Glycosaminoglycan metabolism |
| HS3ST1 | Glycosaminoglycan metabolism |
| HS3ST2 | Glycosaminoglycan metabolism |
| HS3ST3A1 | Glycosaminoglycan metabolism |
| HS3ST3B1 | Glycosaminoglycan metabolism |
| HS3ST4 | Glycosaminoglycan metabolism |
| HS3ST5 | Glycosaminoglycan metabolism |
| HS3ST6 | Glycosaminoglycan metabolism |
| HS6ST1 | Glycosaminoglycan metabolism |
| HS6ST2 | Glycosaminoglycan metabolism |
| HS6ST3 | Glycosaminoglycan metabolism |
| HSD17B10 | Branched-chain amino acid catabolism |
| HSD17B12 | Fatty acid metabolism; Fatty acyl-CoA biosynthesis |
| HSD17B3 | Fatty acid metabolism; Fatty acyl-CoA biosynthesis |
| HSD17B4 | Fatty acid metabolism; Peroxisomal lipid metabolism; β-oxidation of pristanoyl-CoA; β-oxidation of very long chain fatty acids |
| HSD17B8 | Fatty acid metabolism; Fatty acyl-CoA biosynthesis |
| HSP90AA1 | Metabolism of cofactors; Tetrahydrobiopterin (BH4) synthesis, recycling, salvage and regulation |
| HSPG2 | Glycosaminoglycan metabolism; Metabolism of fat-soluble vitamins |
| HTD2 | Fatty acid metabolism; Fatty acyl-CoA biosynthesis |
| HYAL1 | Glycosaminoglycan metabolism |
| HYAL2 | Glycosaminoglycan metabolism |
| HYAL3 | Glycosaminoglycan metabolism |
| HYKK | Lysine catabolism |
| IARS1 | Selenoamino acid metabolism |
| IDH1 | Metabolism of cofactors; NADPH regeneration |
| IDO1 | Tryptophan catabolism |
| IDO2 | Tryptophan catabolism |
| IDS | Glycosaminoglycan metabolism |
| IDUA | Glycosaminoglycan metabolism |
| IMPDH1 | Purine ribonucleoside monophosphate biosynthesis |
| IMPDH2 | Purine ribonucleoside monophosphate biosynthesis |
| INMT | Selenoamino acid metabolism |
| INPP4A | Phospholipid metabolism |
| INPP4B | Phospholipid metabolism |
| INPP5D | Phospholipid metabolism |
| INPP5E | Phospholipid metabolism |
| INPP5F | Phospholipid metabolism |
| INPP5J | Phospholipid metabolism |
| INPP5K | Phospholipid metabolism |
| INPPL1 | Phospholipid metabolism |
| ITPA | Purine catabolism |
| IVD | Branched-chain amino acid catabolism |
| JMJD7-PLA2G4B | Phospholipid metabolism; Glycerophospholipid biosynthesis |
| KARS1 | Selenoamino acid metabolism |
| KERA | Glycosaminoglycan metabolism |
| KMO | Tryptophan catabolism |
| KYAT1 | Tryptophan catabolism |
| KYAT3 | Tryptophan catabolism |
| KYNU | Tryptophan catabolism |
| LALBA | Lactose synthesis |
| LARS1 | Selenoamino acid metabolism |
| LCLAT1 | Phospholipid metabolism; Glycerophospholipid biosynthesis |
| LDLR | Metabolism of fat-soluble vitamins |
| LDLRAP1 | Metabolism of water-soluble vitamins and cofactors; Cobalamin (Cbl, vitamin B12) transport and metabolism |
| LIAS | Glyoxylate metabolism and glycine degradation |
| LIPE | Triglyceride metabolism; Triglyceride catabolism |
| LIPH | Phospholipid metabolism; Glycerophospholipid biosynthesis |
| LIPI | Phospholipid metabolism; Glycerophospholipid biosynthesis |
| LIPT1 | Glyoxylate metabolism and glycine degradation |
| LIPT2 | Glyoxylate metabolism and glycine degradation |
| LMBRD1 | Metabolism of water-soluble vitamins and cofactors; Cobalamin (Cbl, vitamin B12) transport and metabolism |
| LOC102724560 | Sulfur amino acid metabolism; Selenoamino acid metabolism |
| LPCAT1 | Phospholipid metabolism; Glycerophospholipid biosynthesis |
| LPCAT2 | Phospholipid metabolism; Glycerophospholipid biosynthesis |
| LPCAT3 | Phospholipid metabolism; Glycerophospholipid biosynthesis |
| LPCAT4 | Phospholipid metabolism; Glycerophospholipid biosynthesis |
| LPGAT1 | Phospholipid metabolism; Glycerophospholipid biosynthesis |
| LPIN1 | Triglyceride metabolism; Triglyceride biosynthesis; Phospholipid metabolism; Glycerophospholipid biosynthesis |
| LPIN2 | Triglyceride metabolism; Triglyceride biosynthesis; Phospholipid metabolism; Glycerophospholipid biosynthesis |
| LPIN3 | Triglyceride metabolism; Triglyceride biosynthesis; Phospholipid metabolism; Glycerophospholipid biosynthesis |
| LPL | Metabolism of fat-soluble vitamins |
| LRAT | Metabolism of fat-soluble vitamins |
| LRP1 | Metabolism of fat-soluble vitamins |
| LRP10 | Metabolism of fat-soluble vitamins |
| LRP12 | Metabolism of fat-soluble vitamins |
| LRP2 | Metabolism of fat-soluble vitamins |
| LRP8 | Metabolism of fat-soluble vitamins |
| LTA4H | Fatty acid metabolism |
| LTC4S | Fatty acid metabolism |
| LUM | Glycosaminoglycan metabolism |
| LYVE1 | Glycosaminoglycan metabolism |
| MAN2B1 | Lysosomal oligosaccharide catabolism |
| MAN2B2 | Lysosomal oligosaccharide catabolism |
| MAN2C1 | Lysosomal oligosaccharide catabolism |
| MANBA | Lysosomal oligosaccharide catabolism |
| MAOA | Amine Oxidase reactions |
| MAOB | Amine Oxidase reactions |
| MAPKAPK2 | Fatty acid metabolism |
| MARS1 | Selenoamino acid metabolism |
| MAT1A | Sulfur amino acid metabolism; Selenoamino acid metabolism; Methylation |
| MAT2A | Methylation |
| MAT2B | Methylation |
| MBOAT1 | Phospholipid metabolism; Glycerophospholipid biosynthesis |
| MBOAT2 | Phospholipid metabolism; Glycerophospholipid biosynthesis |
| MBOAT7 | Phospholipid metabolism; Glycerophospholipid biosynthesis |
| MCAT | Fatty acid metabolism; Mitochondrial fatty acid β-oxidation |
| MCCC1 | Branched-chain amino acid catabolism; Metabolism of water-soluble vitamins and cofactors |
| MCCC2 | Branched-chain amino acid catabolism; Metabolism of water-soluble vitamins and cofactors |
| MCEE | Fatty acid metabolism; Mitochondrial fatty acid β-oxidation |
| MECR | Fatty acid metabolism; Mitochondrial fatty acid β-oxidation; Mitochondrial fatty acid β-oxidation of saturated fatty acids; β-oxidation of decanoyl-CoA to octanoyl-CoA-CoA |
| MFSD2A | Phospholipid metabolism; Glycerophospholipid biosynthesis |
| MGLL | Triglyceride catabolism; Phospholipid metabolism; Glycerophospholipid biosynthesis |
| MGST1 | Glutathione conjugation |
| MGST2 | Glutathione conjugation |
| MGST3 | Glutathione conjugation |
| MID1IP1 | Fatty acid metabolism; Carnitine metabolism |
| MIGA1 | Phospholipid metabolism; Glycerophospholipid biosynthesis |
| MIGA2 | Phospholipid metabolism; Glycerophospholipid biosynthesis |
| MLYCD | Fatty acid metabolism; Peroxisomal lipid metabolism; β-oxidation of very long chain fatty acids |
| MMAA | Fatty acid metabolism; Mitochondrial fatty acid β-oxidation; Metabolism of water-soluble vitamins and cofactors; Cobalamin (Cbl, vitamin B12) transport and metabolism |
| MMAB | Metabolism of water-soluble vitamins and cofactors; Cobalamin (Cbl, vitamin B12) transport and metabolism |
| MMACHC | Metabolism of water-soluble vitamins and cofactors; Cobalamin (Cbl, vitamin B12) transport and metabolism |
| MMADHC | Metabolism of water-soluble vitamins and cofactors; Cobalamin (Cbl, vitamin B12) transport and metabolism |
| MMUT | Fatty acid metabolism; Mitochondrial fatty acid β-oxidation; Metabolism of water-soluble vitamins and cofactors; Cobalamin (Cbl, vitamin B12) transport and metabolism |
| MOCOS | Metabolism of water-soluble vitamins and cofactors |
| MOCS1 | Metabolism of water-soluble vitamins and cofactors |
| MOCS2 | Metabolism of water-soluble vitamins and cofactors |
| MOCS3 | Metabolism of water-soluble vitamins and cofactors |
| MOGAT1 | Triglyceride biosynthesis |
| MOGAT2 | Triglyceride metabolism; Triglyceride biosynthesis |
| MOGAT3 | Triglyceride metabolism; Triglyceride biosynthesis |
| MORC2 | Fatty acid metabolism; Fatty acyl-CoA biosynthesis |
| MPST | Sulfur amino acid metabolism |
| MRI1 | Sulfur amino acid metabolism |
| MTAP | Sulfur amino acid metabolism |
| MTHFD1 | Metabolism of water-soluble vitamins and cofactors |
| MTHFD1L | Metabolism of water-soluble vitamins and cofactors |
| MTHFD2 | Metabolism of water-soluble vitamins and cofactors |
| MTHFD2L | Metabolism of water-soluble vitamins and cofactors |
| MTHFR | Metabolism of water-soluble vitamins and cofactors |
| MTHFS | Metabolism of water-soluble vitamins and cofactors |
| MTM1 | Phospholipid metabolism |
| MTMR1 | Phospholipid metabolism |
| MTMR10 | Phospholipid metabolism |
| MTMR12 | Phospholipid metabolism |
| MTMR14 | Phospholipid metabolism |
| MTMR2 | Phospholipid metabolism |
| MTMR3 | Phospholipid metabolism |
| MTMR4 | Phospholipid metabolism |
| MTMR6 | Phospholipid metabolism |
| MTMR7 | Phospholipid metabolism |
| MTMR8 | Phospholipid metabolism |
| MTMR9 | Phospholipid metabolism |
| MTR | Sulfur amino acid metabolism; Metabolism of water-soluble vitamins and cofactors; Cobalamin (Cbl, vitamin B12) transport and metabolism; Methylation |
| MTRR | Sulfur amino acid metabolism; Metabolism of water-soluble vitamins and cofactors; Cobalamin (Cbl, vitamin B12) transport and metabolism; Methylation |
| N6AMT1 | Methylation |
| NADK | Metabolism of water-soluble vitamins and cofactors |
| NADK2 | Metabolism of water-soluble vitamins and cofactors |
| NADSYN1 | Metabolism of water-soluble vitamins and cofactors |
| NAGLU | Glycosaminoglycan metabolism |
| NAGS | Urea cycle |
| NAMPT | Metabolism of water-soluble vitamins and cofactors |
| NAPRT | Metabolism of water-soluble vitamins and cofactors |
| NAT1 | Acetylation |
| NAT2 | Acetylation |
| NAXD | Metabolism of water-soluble vitamins and cofactors |
| NAXE | Metabolism of water-soluble vitamins and cofactors |
| NCAN | Glycosaminoglycan metabolism |
| NDST1 | Glycosaminoglycan metabolism |
| NDST2 | Glycosaminoglycan metabolism |
| NDST3 | Glycosaminoglycan metabolism |
| NDST4 | Glycosaminoglycan metabolism |
| NDUFAB1 | Glyoxylate metabolism and glycine degradation; Fatty acid metabolism; Mitochondrial fatty acid β-oxidation |
| NFS1 | Metabolism of water-soluble vitamins and cofactors |
| NMNAT1 | Metabolism of water-soluble vitamins and cofactors |
| NMNAT2 | Metabolism of water-soluble vitamins and cofactors |
| NMNAT3 | Metabolism of water-soluble vitamins and cofactors |
| NMRAL1 | Urea cycle |
| NMRK1 | Metabolism of water-soluble vitamins and cofactors |
| NMRK2 | Metabolism of water-soluble vitamins and cofactors |
| NNMT | Selenoamino acid metabolism; Metabolism of water-soluble vitamins and cofactors; Methylation |
| NOS3 | Metabolism of cofactors; Tetrahydrobiopterin (BH4) synthesis, recycling, salvage and regulation |
| NQO1 | Metabolism of polyamines |
| NT5C | Purine catabolism; Pyrimidine catabolism |
| NT5C1A | Purine catabolism; Pyrimidine catabolism |
| NT5C1B | Purine catabolism |
| NT5C1B-RDH14 | Purine catabolism |
| NT5C2 | Purine catabolism |
| NT5C3A | Pyrimidine catabolism |
| NT5E | Purine catabolism; Pyrimidine catabolism; Metabolism of water-soluble vitamins and cofactors |
| NT5M | Pyrimidine catabolism |
| NUDT1 | Purine catabolism |
| NUDT12 | Metabolism of water-soluble vitamins and cofactors |
| NUDT15 | Purine catabolism |
| NUDT16 | Purine catabolism |
| NUDT18 | Purine catabolism |
| NUDT19 | Fatty acid metabolism; Peroxisomal lipid metabolism |
| NUDT5 | Purine catabolism |
| NUDT7 | Fatty acid metabolism; Peroxisomal lipid metabolism |
| NUDT9 | Purine catabolism |
| OAZ1 | Metabolism of polyamines |
| OAZ2 | Metabolism of polyamines |
| OAZ3 | Metabolism of polyamines |
| OCRL | Phospholipid metabolism |
| ODC1 | Metabolism of polyamines |
| OGDH | Lysine catabolism; Glyoxylate metabolism and glycine degradation |
| OGN | Glycosaminoglycan metabolism |
| OLAH | Fatty acid metabolism; Fatty acyl-CoA biosynthesis |
| OMD | Glycosaminoglycan metabolism |
| OPLAH | Glutathione conjugation |
| OSBPL10 | Phospholipid metabolism; Glycerophospholipid biosynthesis |
| OSBPL5 | Phospholipid metabolism; Glycerophospholipid biosynthesis |
| OSBPL8 | Phospholipid metabolism; Glycerophospholipid biosynthesis |
| OTC | Urea cycle |
| OXCT1 | Ketone body metabolism; Utilization of Ketone Bodies |
| OXCT2 | Ketone body metabolism; Utilization of Ketone Bodies |
| PAICS | Purine ribonucleoside monophosphate biosynthesis |
| PANK1 | Metabolism of water-soluble vitamins and cofactors; Vitamin B5 pantothenate metabolism |
| PANK2 | Metabolism of water-soluble vitamins and cofactors; Vitamin B5 pantothenate metabolism |
| PANK3 | Metabolism of water-soluble vitamins and cofactors; Vitamin B5 pantothenate metabolism |
| PANK4 | Metabolism of water-soluble vitamins and cofactors; Vitamin B5 pantothenate metabolism |
| PAOX | Metabolism of polyamines; Amine Oxidase reactions |
| PAPSS1 | Selenoamino acid metabolism; Glycosaminoglycan metabolism; Cytosolic sulfonation of small molecules |
| PAPSS2 | Selenoamino acid metabolism; Glycosaminoglycan metabolism; Cytosolic sulfonation of small molecules |
| PARP10 | Metabolism of water-soluble vitamins and cofactors |
| PARP14 | Metabolism of water-soluble vitamins and cofactors |
| PARP16 | Metabolism of water-soluble vitamins and cofactors |
| PARP4 | Metabolism of water-soluble vitamins and cofactors |
| PARP6 | Metabolism of water-soluble vitamins and cofactors |
| PARP8 | Metabolism of water-soluble vitamins and cofactors |
| PARP9 | Metabolism of water-soluble vitamins and cofactors |
| PC | Metabolism of water-soluble vitamins and cofactors |
| PCCA | Fatty acid metabolism; Mitochondrial fatty acid β-oxidation; Metabolism of water-soluble vitamins and cofactors |
| PCCB | Fatty acid metabolism; Mitochondrial fatty acid β-oxidation; Metabolism of water-soluble vitamins and cofactors |
| PCTP | Fatty acid metabolism; Mitochondrial fatty acid β-oxidation; Phospholipid metabolism; Glycerophospholipid biosynthesis |
| PCYT1A | Phospholipid metabolism; Glycerophospholipid biosynthesis |
| PCYT1B | Phospholipid metabolism; Glycerophospholipid biosynthesis |
| PCYT2 | Phospholipid metabolism; Glycerophospholipid biosynthesis |
| PDHA1 | Glyoxylate metabolism and glycine degradation |
| PDHA2 | Glyoxylate metabolism and glycine degradation |
| PDHB | Glyoxylate metabolism and glycine degradation |
| PDHX | Glyoxylate metabolism and glycine degradation |
| PDSS1 | Metabolism of cofactors; Ubiquinol biosynthesis |
| PDSS2 | Metabolism of cofactors; Ubiquinol biosynthesis |
| PDXK | Metabolism of water-soluble vitamins and cofactors |
| PDZD11 | Metabolism of water-soluble vitamins and cofactors; Vitamin B5 pantothenate metabolism |
| PECR | Fatty acid metabolism; Peroxisomal lipid metabolism |
| PEMT | Phospholipid metabolism; Glycerophospholipid biosynthesis |
| PFAS | Purine ribonucleoside monophosphate biosynthesis |
| PGS1 | Phospholipid metabolism; Glycerophospholipid biosynthesis |
| PHOSPHO1 | Phospholipid metabolism; Glycerophospholipid biosynthesis |
| PHYH | Fatty acid metabolism; Peroxisomal lipid metabolism |
| PHYKPL | Lysine catabolism |
| PI4K2A | Phospholipid metabolism |
| PI4K2B | Phospholipid metabolism |
| PI4KA | Phospholipid metabolism |
| PI4KB | Phospholipid metabolism |
| PIK3C2A | Phospholipid metabolism |
| PIK3C2B | Phospholipid metabolism |
| PIK3C2G | Phospholipid metabolism |
| PIK3C3 | Phospholipid metabolism |
| PIK3CA | Phospholipid metabolism |
| PIK3CB | Phospholipid metabolism |
| PIK3CD | Phospholipid metabolism |
| PIK3CG | Phospholipid metabolism |
| PIK3R1 | Phospholipid metabolism |
| PIK3R2 | Phospholipid metabolism |
| PIK3R3 | Phospholipid metabolism |
| PIK3R4 | Phospholipid metabolism |
| PIK3R5 | Phospholipid metabolism |
| PIK3R6 | Phospholipid metabolism |
| PIKFYVE | Phospholipid metabolism |
| PIP4K2A | Phospholipid metabolism |
| PIP4K2B | Phospholipid metabolism |
| PIP4K2C | Phospholipid metabolism |
| PIP4P1 | Phospholipid metabolism |
| PIP5K1A | Phospholipid metabolism |
| PIP5K1B | Phospholipid metabolism |
| PIP5K1C | Phospholipid metabolism |
| PIPOX | Lysine catabolism |
| PISD | Phospholipid metabolism;Glycerophospholipid biosynthesis |
| PITPNB | Phospholipid metabolism;Glycerophospholipid biosynthesis |
| PITPNM1 | Phospholipid metabolism;Glycerophospholipid biosynthesis |
| PITPNM2 | Phospholipid metabolism;Glycerophospholipid biosynthesis |
| PITPNM3 | Phospholipid metabolism;Glycerophospholipid biosynthesis |
| PLA1A | Phospholipid metabolism;Glycerophospholipid biosynthesis |
| PLA2G10 | Phospholipid metabolism;Glycerophospholipid biosynthesis |
| PLA2G12A | Phospholipid metabolism;Glycerophospholipid biosynthesis |
| PLA2G15 | Phospholipid metabolism;Glycerophospholipid biosynthesis |
| PLA2G1B | Phospholipid metabolism;Glycerophospholipid biosynthesis |
| PLA2G2A | Phospholipid metabolism;Glycerophospholipid biosynthesis |
| PLA2G2D | Phospholipid metabolism;Glycerophospholipid biosynthesis |
| PLA2G2E | Phospholipid metabolism;Glycerophospholipid biosynthesis |
| PLA2G2F | Phospholipid metabolism;Glycerophospholipid biosynthesis |
| PLA2G3 | Phospholipid metabolism;Glycerophospholipid biosynthesis |
| PLA2G4A | Phospholipid metabolism;Glycerophospholipid biosynthesis |
| PLA2G4B | Phospholipid metabolism;Glycerophospholipid biosynthesis |
| PLA2G4C | Phospholipid metabolism;Glycerophospholipid biosynthesis |
| PLA2G4D | Phospholipid metabolism;Glycerophospholipid biosynthesis |
| PLA2G4E | Phospholipid metabolism;Glycerophospholipid biosynthesis |
| PLA2G4F | Phospholipid metabolism;Glycerophospholipid biosynthesis |
| PLA2G5 | Phospholipid metabolism;Glycerophospholipid biosynthesis |
| PLA2G6 | Phospholipid metabolism;Glycerophospholipid biosynthesis |
| PLA2R1 | Phospholipid metabolism;Glycerophospholipid biosynthesis |
| PLAAT1 | Phospholipid metabolism;Glycerophospholipid biosynthesis |
| PLAAT2 | Phospholipid metabolism;Glycerophospholipid biosynthesis |
| PLAAT3 | Phospholipid metabolism;Glycerophospholipid biosynthesis |
| PLAAT4 | Phospholipid metabolism;Glycerophospholipid biosynthesis |
| PLAAT5 | Phospholipid metabolism;Glycerophospholipid biosynthesis |
| PLB1 | Phospholipid metabolism;Glycerophospholipid biosynthesis;Metabolism of fat-soluble vitamins |
| PLBD1 | Phospholipid metabolism;Glycerophospholipid biosynthesis |
| PLD1 | Phospholipid metabolism;Glycerophospholipid biosynthesis |
| PLD2 | Phospholipid metabolism;Glycerophospholipid biosynthesis |
| PLD3 | Phospholipid metabolism;Glycerophospholipid biosynthesis |
| PLD4 | Phospholipid metabolism;Glycerophospholipid biosynthesis |
| PLD6 | Phospholipid metabolism;Glycerophospholipid biosynthesis |
| PLEKHA1 | Phospholipid metabolism |
| PLEKHA2 | Phospholipid metabolism |
| PLEKHA3 | Phospholipid metabolism |
| PLEKHA4 | Phospholipid metabolism |
| PLEKHA5 | Phospholipid metabolism |
| PLEKHA6 | Phospholipid metabolism |
| PLEKHA8 | Phospholipid metabolism |
| PLIN1 | Triglyceride metabolism;Triglyceride catabolism |
| PLIN3 | Triglyceride catabolism |
| PNLIP | Metabolism of fat-soluble vitamins |
| PNP | Purine salvage;Purine catabolism |
| PNPLA2 | Phospholipid metabolism;Glycerophospholipid biosynthesis |
| PNPLA3 | Phospholipid metabolism;Glycerophospholipid biosynthesis |
| PNPLA4 | Triglyceride metabolism;Triglyceride catabolism |
| PNPLA5 | Triglyceride metabolism;Triglyceride catabolism |
| PNPLA6 | Phospholipid metabolism;Glycerophospholipid catabolism |
| PNPLA7 | Phospholipid metabolism;Glycerophospholipid catabolism |
| PNPLA8 | Phospholipid metabolism;Glycerophospholipid biosynthesis |
| PNPO | Metabolism of water-soluble vitamins and cofactors |
| PODXL2 | Cytosolic sulfonation of small molecules |
| PON1 | Fatty acid metabolism |
| PON2 | Fatty acid metabolism |
| PON3 | Fatty acid metabolism |
| PPARD | Fatty acid metabolism;Carnitine metabolism |
| PPAT | Purine ribonucleoside monophosphate biosynthesis |
| PPCDC | Metabolism of water-soluble vitamins and cofactors;Vitamin B5 pantothenate metabolism |
| PPCS | Metabolism of water-soluble vitamins and cofactors;Vitamin B6 pantothenate metabolism |
| PPM1K | Branched-chain amino acid catabolism |
| PPP1CA | Triglyceride metabolism;Triglyceride catabolism |
| PPP1CB | Triglyceride metabolism;Triglyceride catabolism |
| PPP1CC | Triglyceride metabolism;Triglyceride catabolism |
| PPT1 | Fatty acid metabolism;Fatty acyl-CoA biosynthesis |
| PPT2 | Fatty acid metabolism;Fatty acyl-CoA biosynthesis |
| PRELP | Glycosaminoglycan metabolism |
| PRKAA2 | Fatty acid metabolism;Carnitine metabolism |
| PRKAB2 | Fatty acid metabolism;Carnitine metabolism |
| PRKACA | Triglyceride metabolism;Triglyceride catabolism |
| PRKACB | Triglyceride metabolism;Triglyceride catabolism |
| PRKACG | Triglyceride metabolism;Triglyceride catabolism |
| PRKAG2 | Fatty acid metabolism;Carnitine metabolism |
| PRKG2 | Metabolism of cofactors;Tetrahydrobiopterin (BH4) synthesis, recycling, salvage and regulation |
| PRODH | Proline catabolism |
| PRODH2 | Proline catabolism;Glyoxylate metabolism and glycine degradation |
| PRSS1 | Metabolism of water-soluble vitamins and cofactors;Cobalamin (Cbl, vitamin B12) transport and metabolism |
| PRSS3 | Metabolism of water-soluble vitamins and cofactors;Cobalamin (Cbl, vitamin B13) transport and metabolism |
| PRXL2B | Fatty acid metabolism |
| PSMA1 | Metabolism of polyamines |
| PSMA2 | Metabolism of polyamines |
| PSMA3 | Metabolism of polyamines |
| PSMA4 | Metabolism of polyamines |
| PSMA5 | Metabolism of polyamines |
| PSMA6 | Metabolism of polyamines |
| PSMA7 | Metabolism of polyamines |
| PSMA8 | Metabolism of polyamines |
| PSMB1 | Metabolism of polyamines |
| PSMB10 | Metabolism of polyamines |
| PSMB11 | Metabolism of polyamines |
| PSMB2 | Metabolism of polyamines |
| PSMB3 | Metabolism of polyamines |
| PSMB4 | Metabolism of polyamines |
| PSMB5 | Metabolism of polyamines |
| PSMB6 | Metabolism of polyamines |
| PSMB7 | Metabolism of polyamines |
| PSMB8 | Metabolism of polyamines |
| PSMB9 | Metabolism of polyamines |
| PSMC1 | Metabolism of polyamines |
| PSMC2 | Metabolism of polyamines |
| PSMC3 | Metabolism of polyamines |
| PSMC4 | Metabolism of polyamines |
| PSMC5 | Metabolism of polyamines |
| PSMC6 | Metabolism of polyamines |
| PSMD1 | Metabolism of polyamines |
| PSMD10 | Metabolism of polyamines |
| PSMD11 | Metabolism of polyamines |
| PSMD12 | Metabolism of polyamines |
| PSMD13 | Metabolism of polyamines |
| PSMD14 | Metabolism of polyamines |
| PSMD2 | Metabolism of polyamines |
| PSMD3 | Metabolism of polyamines |
| PSMD4 | Metabolism of polyamines |
| PSMD5 | Metabolism of polyamines |
| PSMD6 | Metabolism of polyamines |
| PSMD7 | Metabolism of polyamines |
| PSMD8 | Metabolism of polyamines |
| PSMD9 | Metabolism of polyamines |
| PSME1 | Metabolism of polyamines |
| PSME2 | Metabolism of polyamines |
| PSME3 | Metabolism of polyamines |
| PSME4 | Metabolism of polyamines |
| PSMF1 | Metabolism of polyamines |
| PSTK | Selenoamino acid metabolism |
| PTDSS1 | Phospholipid metabolism;Glycerophospholipid biosynthesis |
| PTDSS2 | Phospholipid metabolism;Glycerophospholipid biosynthesis |
| PTEN | Phospholipid metabolism |
| PTGDS | Fatty acid metabolism |
| PTGES | Fatty acid metabolism |
| PTGES2 | Fatty acid metabolism |
| PTGES3 | Fatty acid metabolism |
| PTGIS | Fatty acid metabolism;Metabolism of water-soluble vitamins and cofactors |
| PTGR1 | Fatty acid metabolism |
| PTGR2 | Fatty acid metabolism |
| PTGS1 | Fatty acid metabolism;COX reactions |
| PTGS2 | Fatty acid metabolism;Metabolism of water-soluble vitamins and cofactors |
| PTPMT1 | Phospholipid metabolism;Glycerophospholipid biosynthesis |
| PTPN13 | Phospholipid metabolism |
| PTS | Metabolism of cofactors;Tetrahydrobiopterin (BH4) synthesis, recycling, salvage and regulation |
| PUDP | Pyrimidine salvage |
| PXMP2 | Glyoxylate metabolism and glycine degradation |
| QARS1 | Selenoamino acid metabolism |
| QPRT | Metabolism of water-soluble vitamins and cofactors |
| RAB14 | Phospholipid metabolism |
| RAB4A | Phospholipid metabolism |
| RAB5A | Phospholipid metabolism |
| RARS1 | Selenoamino acid metabolism |
| RBP1 | Metabolism of fat-soluble vitamins |
| RBP2 | Metabolism of fat-soluble vitamins |
| RBP4 | Metabolism of fat-soluble vitamins |
| RDH11 | Metabolism of fat-soluble vitamins |
| RETSAT | Metabolism of fat-soluble vitamins |
| RFK | Metabolism of water-soluble vitamins and cofactors |
| RIDA | Threonine catabolism |
| RNLS | Metabolism of water-soluble vitamins and cofactors |
| RPL10 | Selenoamino acid metabolism |
| RPL10A | Selenoamino acid metabolism |
| RPL10L | Selenoamino acid metabolism |
| RPL11 | Selenoamino acid metabolism |
| RPL12 | Selenoamino acid metabolism |
| RPL13 | Selenoamino acid metabolism |
| RPL13A | Selenoamino acid metabolism |
| RPL14 | Selenoamino acid metabolism |
| RPL15 | Selenoamino acid metabolism |
| RPL17 | Selenoamino acid metabolism |
| RPL18 | Selenoamino acid metabolism |
| RPL18A | Selenoamino acid metabolism |
| RPL19 | Selenoamino acid metabolism |
| RPL21 | Selenoamino acid metabolism |
| RPL22 | Selenoamino acid metabolism |
| RPL22L1 | Selenoamino acid metabolism |
| RPL23 | Selenoamino acid metabolism |
| RPL23A | Selenoamino acid metabolism |
| RPL24 | Selenoamino acid metabolism |
| RPL26 | Selenoamino acid metabolism |
| RPL26L1 | Selenoamino acid metabolism |
| RPL27 | Selenoamino acid metabolism |
| RPL27A | Selenoamino acid metabolism |
| RPL28 | Selenoamino acid metabolism |
| RPL29 | Selenoamino acid metabolism |
| RPL3 | Selenoamino acid metabolism |
| RPL30 | Selenoamino acid metabolism |
| RPL31 | Selenoamino acid metabolism |
| RPL32 | Selenoamino acid metabolism |
| RPL34 | Selenoamino acid metabolism |
| RPL35 | Selenoamino acid metabolism |
| RPL35A | Selenoamino acid metabolism |
| RPL36 | Selenoamino acid metabolism |
| RPL36A | Selenoamino acid metabolism |
| RPL36AL | Selenoamino acid metabolism |
| RPL37 | Selenoamino acid metabolism |
| RPL37A | Selenoamino acid metabolism |
| RPL38 | Selenoamino acid metabolism |
| RPL39 | Selenoamino acid metabolism |
| RPL39L | Selenoamino acid metabolism |
| RPL3L | Selenoamino acid metabolism |
| RPL4 | Selenoamino acid metabolism |
| RPL41 | Selenoamino acid metabolism |
| RPL5 | Selenoamino acid metabolism |
| RPL6 | Selenoamino acid metabolism |
| RPL7 | Selenoamino acid metabolism |
| RPL7A | Selenoamino acid metabolism |
| RPL8 | Selenoamino acid metabolism |
| RPL9 | Selenoamino acid metabolism |
| RPLP0 | Selenoamino acid metabolism |
| RPLP1 | Selenoamino acid metabolism |
| RPLP2 | Selenoamino acid metabolism |
| RPS10 | Selenoamino acid metabolism |
| RPS11 | Selenoamino acid metabolism |
| RPS12 | Selenoamino acid metabolism |
| RPS13 | Selenoamino acid metabolism |
| RPS14 | Selenoamino acid metabolism |
| RPS15 | Selenoamino acid metabolism |
| RPS15A | Selenoamino acid metabolism |
| RPS16 | Selenoamino acid metabolism |
| RPS17 | Selenoamino acid metabolism |
| RPS18 | Selenoamino acid metabolism |
| RPS19 | Selenoamino acid metabolism |
| RPS2 | Selenoamino acid metabolism |
| RPS20 | Selenoamino acid metabolism |
| RPS21 | Selenoamino acid metabolism |
| RPS23 | Selenoamino acid metabolism |
| RPS24 | Selenoamino acid metabolism |
| RPS25 | Selenoamino acid metabolism |
| RPS26 | Selenoamino acid metabolism |
| RPS27 | Selenoamino acid metabolism |
| RPS27A | Selenoamino acid metabolism |
| RPS27L | Selenoamino acid metabolism |
| RPS28 | Selenoamino acid metabolism |
| RPS29 | Selenoamino acid metabolism |
| RPS3 | Selenoamino acid metabolism |
| RPS3A | Selenoamino acid metabolism |
| RPS4X | Selenoamino acid metabolism |
| RPS4Y1 | Selenoamino acid metabolism |
| RPS4Y2 | Selenoamino acid metabolism |
| RPS5 | Selenoamino acid metabolism |
| RPS6 | Selenoamino acid metabolism |
| RPS7 | Selenoamino acid metabolism |
| RPS8 | Selenoamino acid metabolism |
| RPS9 | Selenoamino acid metabolism |
| RPSA | Selenoamino acid metabolism |
| RUFY1 | Phospholipid metabolism |
| RXRA | Fatty acid metabolism;Carnitine metabolism |
| SACM1L | Phospholipid metabolism |
| SARDH | Choline catabolism |
| SARS1 | Selenoamino acid metabolism |
| SAT1 | Metabolism of polyamines |
| SBF1 | Phospholipid metabolism |
| SBF2 | Phospholipid metabolism |
| SCD | Fatty acid metabolism;Fatty acyl-CoA biosynthesis |
| SCD5 | Fatty acid metabolism;Fatty acyl-CoA biosynthesis |
| SCLY | Selenoamino acid metabolism |
| SCP2 | Fatty acid metabolism;Peroxisomal lipid metabolism;β-oxidation of pristanoyl-CoA |
| SDC1 | Glycosaminoglycan metabolism;Metabolism of fat-soluble vitamins |
| SDC2 | Glycosaminoglycan metabolism;Metabolism of fat-soluble vitamins |
| SDC3 | Glycosaminoglycan metabolism;Metabolism of fat-soluble vitamins |
| SDC4 | Glycosaminoglycan metabolism;Metabolism of fat-soluble vitamins |
| SDS | Threonine catabolism |
| SDSL | Threonine catabolism |
| SECISBP2 | Selenoamino acid metabolism |
| SELENOI | Phospholipid metabolism;Glycerophospholipid biosynthesis |
| SEM1 | Metabolism of polyamines |
| SEPHS2 | Selenoamino acid metabolism |
| SEPSECS | Selenoamino acid metabolism |
| SGSH | Glycosaminoglycan metabolism |
| SHMT1 | Carnitine synthesis;Metabolism of water-soluble vitamins and cofactors |
| SHMT2 | Metabolism of water-soluble vitamins and cofactors |
| SLC19A1 | Metabolism of water-soluble vitamins and cofactors |
| SLC19A2 | Metabolism of water-soluble vitamins and cofactors |
| SLC19A3 | Metabolism of water-soluble vitamins and cofactors |
| SLC22A13 | Metabolism of water-soluble vitamins and cofactors |
| SLC22A5 | Fatty acid metabolism;Carnitine metabolism |
| SLC23A1 | Metabolism of water-soluble vitamins and cofactors;Vitamin C ascorbate metabolism |
| SLC23A2 | Metabolism of water-soluble vitamins and cofactors;Vitamin C ascorbate metabolism |
| SLC25A1 | Fatty acid metabolism;Fatty acyl-CoA biosynthesis |
| SLC25A10 | Sulfur amino acid metabolism |
| SLC25A15 | Urea cycle |
| SLC25A16 | Metabolism of water-soluble vitamins and cofactors;Vitamin B5 pantothenate metabolism |
| SLC25A17 | Fatty acid metabolism;Peroxisomal lipid metabolism |
| SLC25A19 | Metabolism of water-soluble vitamins and cofactors |
| SLC25A2 | Urea cycle |
| SLC25A20 | Fatty acid metabolism;Carnitine metabolism |
| SLC25A21 | Lysine catabolism |
| SLC25A32 | Metabolism of water-soluble vitamins and cofactors |
| SLC25A44 | Branched-chain amino acid catabolism |
| SLC26A1 | Glycosaminoglycan metabolism;Cytosolic sulfonation of small molecules |
| SLC26A2 | Glycosaminoglycan metabolism;Cytosolic sulfonation of small molecules |
| SLC27A2 | Fatty acid metabolism;Fatty acyl-CoA biosynthesis;Peroxisomal lipid metabolism |
| SLC27A3 | Fatty acid metabolism;Fatty acyl-CoA biosynthesis |
| SLC2A1 | Lactose synthesis;Metabolism of water-soluble vitamins and cofactors;Vitamin C ascorbate metabolism |
| SLC2A3 | Metabolism of water-soluble vitamins and cofactors;Vitamin C ascorbate metabolism |
| SLC35B2 | Glycosaminoglycan metabolism;Cytosolic sulfonation of small molecules |
| SLC35B3 | Glycosaminoglycan metabolism;Cytosolic sulfonation of small molecules |
| SLC35D1 | Glucuronidation |
| SLC35D2 | Glycosaminoglycan metabolism |
| SLC36A4 | Tryptophan catabolism |
| SLC3A2 | Tryptophan catabolism |
| SLC44A1 | Choline catabolism;Phospholipid metabolism;Glycerophospholipid biosynthesis |
| SLC44A2 | Phospholipid metabolism;Glycerophospholipid biosynthesis |
| SLC44A3 | Phospholipid metabolism;Glycerophospholipid biosynthesis |
| SLC44A4 | Phospholipid metabolism;Glycerophospholipid biosynthesis |
| SLC44A5 | Phospholipid metabolism;Glycerophospholipid biosynthesis |
| SLC46A1 | Metabolism of water-soluble vitamins and cofactors |
| SLC52A1 | Metabolism of water-soluble vitamins and cofactors |
| SLC52A2 | Metabolism of water-soluble vitamins and cofactors |
| SLC52A3 | Metabolism of water-soluble vitamins and cofactors |
| SLC5A6 | Metabolism of water-soluble vitamins and cofactors;Vitamin B5 pantothenate metabolism |
| SLC5A8 | Metabolism of water-soluble vitamins and cofactors |
| SLC6A11 | Creatine metabolism |
| SLC6A12 | Creatine metabolism |
| SLC6A7 | Creatine metabolism |
| SLC6A8 | Creatine metabolism |
| SLC7A5 | Tryptophan catabolism |
| SLC9A1 | Glycosaminoglycan metabolism |
| SMOX | Metabolism of polyamines;Amine Oxidase reactions |
| SMS | Metabolism of polyamines |
| SORD | Formation of xylulose-5-phosphate |
| SPR | Metabolism of cofactors;Tetrahydrobiopterin (BH4) synthesis, recycling, salvage and regulation |
| SQOR | Sulfur amino acid metabolism |
| SRM | Metabolism of polyamines |
| ST3GAL1 | Glycosaminoglycan metabolism |
| ST3GAL2 | Glycosaminoglycan metabolism |
| ST3GAL3 | Glycosaminoglycan metabolism |
| ST3GAL4 | Glycosaminoglycan metabolism |
| ST3GAL6 | Glycosaminoglycan metabolism |
| STAB2 | Glycosaminoglycan metabolism |
| STARD10 | Phospholipid metabolism;Glycerophospholipid biosynthesis |
| STARD7 | Phospholipid metabolism;Glycerophospholipid biosynthesis |
| SULT1A1 | Cytosolic sulfonation of small molecules |
| SULT1A2 | Cytosolic sulfonation of small molecules |
| SULT1A3 | Cytosolic sulfonation of small molecules |
| SULT1A4 | Cytosolic sulfonation of small molecules |
| SULT1B1 | Cytosolic sulfonation of small molecules |
| SULT1C2 | Cytosolic sulfonation of small molecules |
| SULT1C4 | Cytosolic sulfonation of small molecules |
| SULT1E1 | Cytosolic sulfonation of small molecules |
| SULT2A1 | Cytosolic sulfonation of small molecules |
| SULT2B1 | Cytosolic sulfonation of small molecules |
| SULT4A1 | Cytosolic sulfonation of small molecules |
| SULT6B1 | Cytosolic sulfonation of small molecules |
| SUOX | Sulfur amino acid metabolism |
| SYNJ1 | Phospholipid metabolism |
| SYNJ2 | Phospholipid metabolism |
| TAFAZZIN | Phospholipid metabolism;Glycerophospholipid biosynthesis |
| TBXAS1 | Fatty acid metabolism |
| TCN1 | Metabolism of water-soluble vitamins and cofactors;Cobalamin (Cbl, vitamin B12) transport and metabolism |
| TCN2 | Metabolism of water-soluble vitamins and cofactors;Cobalamin (Cbl, vitamin B13) transport and metabolism |
| TDO2 | Tryptophan catabolism |
| TECR | Fatty acid metabolism;Fatty acyl-CoA biosynthesis |
| TECRL | Fatty acid metabolism;Fatty acyl-CoA biosynthesis |
| THEM4 | Fatty acid metabolism;Mitochondrial fatty acid β-oxidation |
| THEM5 | Fatty acid metabolism;Mitochondrial fatty acid β-oxidation |
| THRSP | Fatty acid metabolism;Carnitine metabolism |
| THTPA | Metabolism of water-soluble vitamins and cofactors |
| TK1 | Pyrimidine salvage |
| TK2 | Pyrimidine salvage |
| TMEM86B | Phospholipid metabolism;Glycerophospholipid biosynthesis |
| TMLHE | Carnitine synthesis |
| TNFAIP8 | Phospholipid metabolism |
| TNFAIP8L1 | Phospholipid metabolism |
| TNFAIP8L2 | Phospholipid metabolism |
| TNFAIP8L3 | Phospholipid metabolism |
| TPK1 | Metabolism of water-soluble vitamins and cofactors |
| TPMT | Methylation |
| TPST1 | Cytosolic sulfonation of small molecules |
| TPST2 | Cytosolic sulfonation of small molecules |
| TPTE | Phospholipid metabolism |
| TPTE2 | Phospholipid metabolism |
| TRMT112 | Methylation |
| TST | Sulfur amino acid metabolism |
| TSTD1 | Sulfur amino acid metabolism |
| TTPA | Metabolism of fat-soluble vitamins;Vitamin E |
| TTR | Metabolism of fat-soluble vitamins |
| TXN2 | Sulfur amino acid metabolism |
| TXNRD1 | Selenoamino acid metabolism |
| TYMP | Pyrimidine salvage;Pyrimidine catabolism |
| UBA52 | Selenoamino acid metabolism |
| UBIAD1 | Metabolism of fat-soluble vitamins;Metabolism of vitamin K |
| UCK1 | Pyrimidine salvage |
| UCK2 | Pyrimidine salvage |
| UCKL1 | Pyrimidine salvage |
| UGDH | Glucuronidation |
| UGP2 | Glucuronidation |
| UGT1A1 | Glucuronidation |
| UGT1A10 | Glucuronidation |
| UGT1A3 | Glucuronidation |
| UGT1A4 | Glucuronidation |
| UGT1A5 | Glucuronidation |
| UGT1A6 | Glucuronidation |
| UGT1A7 | Glucuronidation |
| UGT1A8 | Glucuronidation |
| UGT1A9 | Glucuronidation |
| UGT2A1 | Glucuronidation |
| UGT2A2 | Glucuronidation |
| UGT2A3 | Glucuronidation |
| UGT2B10 | Glucuronidation |
| UGT2B11 | Glucuronidation |
| UGT2B15 | Glucuronidation |
| UGT2B17 | Glucuronidation |
| UGT2B28 | Glucuronidation |
| UGT2B4 | Glucuronidation |
| UGT2B7 | Glucuronidation |
| UGT3A1 | Glucuronidation |
| UGT3A2 | Glucuronidation |
| UMPS | Pyrimidine biosynthesis |
| UPB1 | Pyrimidine catabolism |
| UPP1 | Pyrimidine salvage;Pyrimidine catabolism |
| UPP2 | Pyrimidine salvage;Pyrimidine catabolism |
| UROC1 | Histidine catabolism |
| UST | Glycosaminoglycan metabolism |
| VAC14 | Phospholipid metabolism |
| VCAN | Glycosaminoglycan metabolism |
| VKORC1 | Metabolism of fat-soluble vitamins;Metabolism of vitamin K |
| VKORC1L1 | Metabolism of fat-soluble vitamins;Metabolism of vitamin K |
| VNN1 | Metabolism of water-soluble vitamins and cofactors;Vitamin B5 pantothenate metabolism |
| VNN2 | Metabolism of water-soluble vitamins and cofactors;Vitamin B6 pantothenate metabolism |
| XDH | Purine catabolism |
| XYLB | Formation of xylulose-5-phosphate |
| XYLT1 | Glycosaminoglycan metabolism |
| XYLT2 | Glycosaminoglycan metabolism |
