# Supplementary material for: Metabolic-pathway-based classification of tendinopathy reveals a precise therapeutic strategy targeting chondro-modulatory tenocytes
Source: Bone Res. 2026 Aug 27;14:91. doi: 10.1038/s41413-026-00574-w (PMC13522621; doi:10.1038/s41413-026-00574-w)
Supplement: Supplementary file 5 [file 41413_2026_574_MOESM22_ESM.docx]

Table S4. Related to Figure 2. General clinical characteristics among different metabolic-pathway-based classifications of tendinopathy.

|  | | Total | MPC-G | MPC-F | MPC-I | p-value |
| --- | --- | --- | --- | --- | --- | --- |
|  |  | (n=192) | (n=192) | (n=27) | (n=75) |  |
| Age, year |  |  |  |  |  | 0.637 |
|  | <50 | 13  (6.8%) | 7  (7.8%) | 0  (0.0%) | 6  (8.0%) |  |
|  | 50~60 | 56  (29.2%) | 27  (30.0%) | 9  (33.3%) | 20  (26.7%) |  |
|  | 60~70 | 91  (47.4%) | 46  (51.1%) | 11  (40.7%) | 34  (45.3%) |  |
|  | ≥70 | 32  (16.7%) | 10  (11.1%) | 7  (25.9%) | 15  (20.0%) |  |
| Sex, (%) |  |  |  |  |  | 0.607 |
|  | Male | 134 (69.8%) | 61  (67.8%) | 21  (77.8%) | 21  (77.8%) |  |
|  | Female | 58  (30.2%) | 29  (32.2%) | 6  (22.2%) | 6  (22.2%) |  |
| BMI, kg/m^2^ |  |  |  |  |  | <0.001 |
|  | <18.5 | 8  (4.2%) | 3  (3.3%) | 1  (3.7%) | 4  (5.3%) |  |
|  | 18.5~25 | 127  (66.1%) | 58  (64.4%) | 22  (81.5%) | 47  (62.7%) |  |
|  | 25~30 | 49  (25.5%) | 24  (26.7%) | 4  (14.8%) | 21  (28.0%) |  |
|  | ≥30 | 8  (4.2%) | 5  (5.6%) | 0  (0.0%) | 3  (4.0%) |  |
| Affected side, (%) |  |  |  |  |  | 0.343 |
|  | Left | 57  (29.7%) | 31  (34.4%) | 8  (29.6%) | 18  (24.0%) |  |
|  | Right | 135  (70.3%) | 59  (65.6%) | 19  (70.4%) | 57  (76.0%) |  |
| Hypertension, (%) |  |  |  |  |  |  |
|  | Yes | 57  (29.7%) | 26  (28.9%) | 7  (25.9%) | 24  (32.0%) | 0.818 |
|  | No | 135  (70.3%) | 64  (71.1%) | 20  (74.1%) | 51  (68.0%) |  |
| Diabetes mellitus, (%) |  |  |  |  |  | 0.151 |
|  | Yes | 15  (7.8%) | 10  (11.1%) | 0  (0.0%) | 5  (6.7%) |  |
|  | No | 177  (92.2%) | 80  (89.9%) | 27  (100.0%) | 70  (93.3%) |  |
| History of trauma, (%) |  |  |  |  |  | 0.372 |
|  | Yes | 42  (21.9%) | 18  (20.0%) | 4  (14.8%) | 20  (26.7%) |  |
|  | No | 150  (78.1%) | 72  (80.0%) | 23  (85.2%) | 55  (73.3%) |  |
| Duration pain, mo |  | 13.8 ± 30.9 | 12.9 ± 32.6 | 17.5 ± 26.8 | 13.6 ± 30.6 | 0.793 |
| Strength of abduction, lb(N) |  |  |  |  |  |  |
|  | Healthy side | 78.6 ± 33.1 | 79.2 ± 27.3 | 82.7 ± 41.4 | 76.4 ± 34.9 | 0.793 |
|  | Affected side | 40.7 ± 23.6 | 38.3 ± 18.8 | 43.0 ± 33.8 | 42.0 ± 23.0 | 0.704 |
| Strength of enternal rotation, lb(N) |  |  |  |  |  |  |
|  | Healthy side | 75.6 ± 33.0 | 75.3 ± 27.4 | 71.5 ± 34.0 | 77.5 ± 37.8 | 0.817 |
|  | Affected side | 41.1 ± 22.6 | 39.0 ± 17.1 | 44.9 ± 25.0 | 41.5 ± 26.3 | 0.651 |
| Strength of internal rotation, lb(N) |  |  |  |  |  |  |
|  | Healthy side | 81.7 ± 39.2 | 83.3 ± 29.9 | 77.8 ± 37.4 | 90.1 ± 39.2 | 0.432 |
|  | Affected side | 55.6 ± 27.3 | 53.6 ± 24.7 | 62.3 ± 30.1 | 58.4 ± 29.9 | 0.194 |
